# Building a Bird: Musculoskeletal Modeling and Simulation of Wing-Assisted Incline Running During Avian Ontogeny

**DOI:** 10.3389/fbioe.2018.00140

**Published:** 2018-10-23

**Authors:** Ashley M. Heers, Jeffery W. Rankin, John R. Hutchinson

**Affiliations:** ^1^Department of Biological Sciences, California State University Los Angeles, Los Angeles, CA, United States; ^2^Structure and Motion Laboratory, Department of Comparative Biomedical Sciences, The Royal Veterinary College, Hatfield, United Kingdom; ^3^Pathokinesiology Laboratory, Rancho Los Amigos National Rehabilitation Hospital, Downey, CA, United States

**Keywords:** bird, avian, locomotion, ontogeny, development, musculoskeletal modeling, wing-assisted incline running, flight

## Abstract

Flapping flight is the most power-demanding mode of locomotion, associated with a suite of anatomical specializations in extant adult birds. In contrast, many developing birds use their forelimbs to negotiate environments long before acquiring “flight adaptations,” recruiting their developing wings to continuously enhance leg performance and, in some cases, fly. How does anatomical development influence these locomotor behaviors? Isolating morphological contributions to wing performance is extremely challenging using purely empirical approaches. However, musculoskeletal modeling and simulation techniques can incorporate empirical data to explicitly examine the functional consequences of changing morphology by manipulating anatomical parameters individually and estimating their effects on locomotion. To assess how ontogenetic changes in anatomy affect locomotor capacity, we combined existing empirical data on muscle morphology, skeletal kinematics, and aerodynamic force production with advanced biomechanical modeling and simulation techniques to analyze the ontogeny of pectoral limb function in a precocial ground bird (*Alectoris chukar*). Simulations of wing-assisted incline running (WAIR) using these newly developed musculoskeletal models collectively suggest that immature birds have excess muscle capacity and are limited more by feather morphology, possibly because feathers grow more quickly and have a different style of growth than bones and muscles. These results provide critical information about the ontogeny and evolution of avian locomotion by (i) establishing how muscular and aerodynamic forces interface with the skeletal system to generate movement in morphing juvenile birds, and (ii) providing a benchmark to inform biomechanical modeling and simulation of other locomotor behaviors, both across extant species and among extinct theropod dinosaurs.

## Introduction

Darwin described “survival of the fittest,” illustrating how organisms with certain forms might better perform certain functions and have greater fitness, such that form and function are closely linked (Darwin, 1859). Geneticists are exploring “arrival of the fittest”: how novel phenotypes are added to the pool of individuals that encounter selective forces (Gilbert and Epel, 2009). What happens in between these two modes of fitness? We can identify genetic events involved in producing some spectacular adaptations in adults—Darwin's “organs of extreme perfection,” and we have examined how adults that possess such features survive and reproduce. Yet the intermediate stages that bridge embryonic genotypes and adult phenotypes (i.e., post-hatching/postnatal ontogeny), or extinct and extant *bauplans* (i.e., evolution), are often an enigma in functional morphology. What is the advantage of half of a wing or only part of an eye? These types of questions have long fascinated evolutionary biologists (Mivart, 1871), and are also very relevant—though less studied—in developing organisms (Heers and Dial, 2012). This “dilemma of incipient stages” (Gould, 1985) is particularly striking among birds and their theropod dinosaur ancestors.

Flapping flight is the most power-demanding mode of locomotion (Alexander, 2002), and its origin among theropod dinosaurs marks one of the great anatomical transformations in vertebrate history. Whereas early-diverging theropods had no wings and more generalized musculoskeletal anatomies (e.g., *Coelophysis*), extant adult birds have large wings, hypertrophied pectoral muscles, and robust, channelized skeletons that presumably represent adaptations or exaptations for meeting aerial requirements (Gill, 1994; Evans and Heiser, 2004). Between these two extremes lies a succession of animals characterized by increasingly bird-like traits, including “protowings” of various sizes and “transitional” musculoskeletal morphologies [e.g., *Caudipteryx* (Qiang et al., 1998), *Anchiornis* (Hu et al., 2009; Xu et al., 2009)]. This flightless to flight-capable progression is well known but difficult to interpret, because functional attributes of transitional features are challenging to reconstruct.

Though less renowned, an equally dramatic flightless to flight-capable transformation occurs in extant developing birds. Most birds hatch without any semblance of a wing, and the first wing feathers acquired result in small protowings that are less aerodynamically effective than the wings of adults (Dial et al., 2006, 2012; Heers et al., 2011). Compared to adults, immature birds also have small wing muscles (Heers and Dial, 2015), and less channelized skeletons with smaller bony processes for muscle attachment (Heers and Dial, 2012; Heers et al., 2016). These extensive morphological changes rival and in many ways mirror those that occurred during the evolution of flight in extinct dinosaurs (Heers and Dial, 2012). How do such changes influence wing-based locomotion?

Growing evidence demonstrates that precocial—and sometimes altricial—birds use their wings to negotiate environments early in ontogeny, long before acquiring the anatomical hallmarks of advanced flight capacity (Dial et al., 2006; Heers and Dial, 2012). These juveniles recruit their developing wings to enhance leg performance and eventually fly. For example, fledglings flap their rudimentary wings to (i) increase foot traction and ascend steep inclines [wing-assisted incline running, i.e., WAIR (Dial, 2003)], (ii) control aerial descents (Dial et al., 2008; Evangelista et al., 2014), (iii) swim (Thomas, 1996) or “steam” across water (Dial and Carrier, 2012), and/or (iv) increase jump height (Heers and Dial, 2015). In some species, such as the Chukar Partridge (*Alectoris chukar*), precocial fledglings can even fly, and display adult kinematics months before acquiring mature wings and musculoskeletal apparatuses (~18–20 vs. ~60–100 days) (Heers and Dial, 2015). Thus, on both ontogenetic and evolutionary time scales, there is a gradient of flight capacity and the degree of anatomical specialization necessary for adult-like, “avian” locomotion is not clear.

Much of this uncertainty stems from the difficulty in experimentally determining how specific morphological features contribute to locomotion. Across species, extant adult birds may not offer enough variation in anatomy and aerial performance to clearly reveal form-function relationships, because most volant adults share a similar array of anatomical specializations. Morphology varies much more through ontogeny but wings, muscles, and skeletons develop simultaneously and can only be altered to certain extents (e.g., trimming feathers, muscle denervation). Consequently, using empirical approaches to isolate morphological contributions to flight capacity and to extrapolate these form-function relationships to extinct animals is extremely challenging.

**Figure 1:**
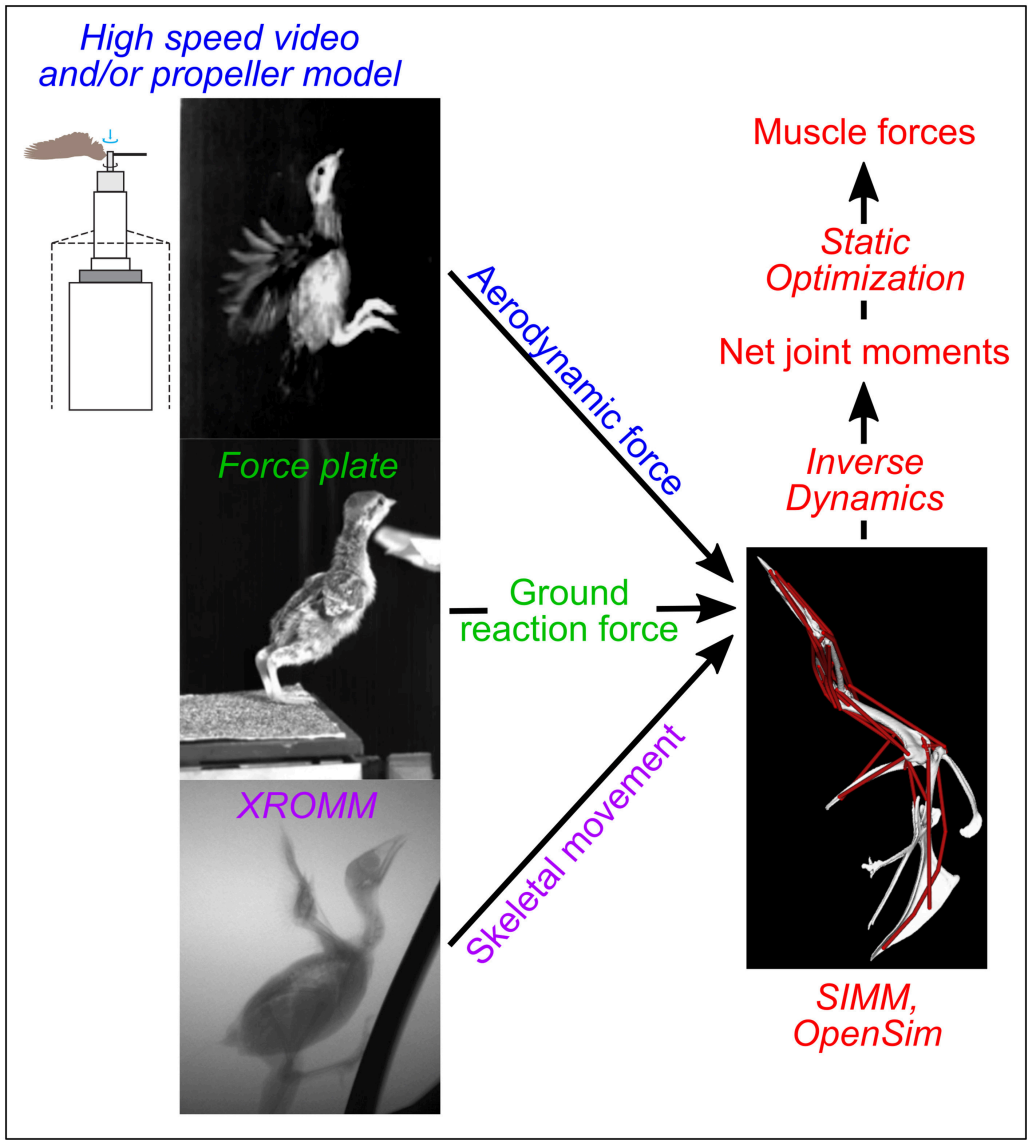
Modeling overview. Colors indicate different types of data used as simulation inputs or outputs; techniques used to collect or simulate each type of data are italicized. SIMM, Software for Interactive Musculoskeletal Modeling; XROMM, X-ray Reconstruction of Moving Morphology. The drawing of a propeller apparatus is modified from Crandell and Tobalske (2011).

In contrast, modeling and simulation approaches can augment empirical studies by isolating and elucidating form-function relationships in ways that are not possible working with live subjects. These approaches build on empirical work by using computed tomographic (CT) scans and dissections to construct digital musculoskeletal models that are paired with kinematic, kinetic, and physiological data to simulate locomotion. Once the validity of the framework is assessed (e.g., by comparing final computer simulation outputs with *in vivo* data), models and simulations can identify how changes in each input (e.g., anatomy, kinematics) independently affect function (e.g., locomotor performance). For instance, Holzbaur et al. (2005) constructed a musculoskeletal model of the human forelimb and simulated muscular force development for muscle-tendon configurations representing pre- and post-surgery conditions to predict the effects of tendon transfer surgery, with good success. Similarly, O'Neill et al. (2013) adjusted muscle origins in a chimpanzee (*Pan troglodytes*) model to assess relationships between muscle attachment sites and moment arms. More sophisticated predictive simulation studies have also been conducted, such as estimating how a *Tyrannosaurus rex* might have moved using forward dynamics (Sellers et al., 2017). A theoretical modeling and simulation framework to investigate avian wing biomechanics can likewise be established by constructing musculoskeletal models and simulating locomotion using different anatomical and/or biomechanical inputs (Figure 1).

To better understand the independent contributions of different morphological specializations to avian locomotor performance, here we combined existing data on muscle morphology, skeletal kinematics, and aerodynamic force production to construct musculoskeletal models of an avian wing at different ontogenetic stages. Simulations of WAIR were then used to analyze pectoral limb function in models of “baby” (7–8 days post-hatch), “juvenile” (18–20 days post-hatch), and adult (>100 days post-hatch) Chukar Partridges. These precocial ground birds are a model species for locomotor ontogeny. Although adult chukars use their wings for a variety of locomotor behaviors, WAIR is one of the few flapping behaviors used at all stages of ontogeny and represents a challenging and particularly important behavior for developing birds in many species (Dial et al., 2015). Behaviors like WAIR allow incipiently volant juveniles to reach otherwise inaccessible elevated refuges, and seamlessly transition from leg-based terrestrial locomotion to wing-based aerial locomotion by recruiting their wings and legs cooperatively. Similar behaviors may have played an important role during the evolution of avian flight, because immature birds and pennaraptoran theropods have many features in common and are (or were) both in the process of acquiring “flight adaptations” and flight capacity (Heers and Dial, 2012).

We used our musculoskeletal models and simulations to examine how anatomical specialization influences the mechanics of flap-running on 65° inclines by testing the following hypotheses concerning relationships between wing capacity and skeletal, muscle, and feather morphology:

*H*_0_*: musculoskeletal and feather morphology equally limit locomotor capacity (the ability to perform a specific behavior, i.e., WAIR) in developing chukars*. If this null hypothesis is not rejected, then the pectoralis (main downstroke muscle) and supracoracoideus (main upstroke muscle) should be maximally, or close to maximally, activated during simulations of WAIR. High activations are expected because immature chukars would be recruiting their muscles to maximal levels to flap their wings as fast as possible, to produce as much aerodynamic force as possible. This prediction assumes that (i) WAIR is a challenging behavior for developing chukars, and that (ii) bigger or better muscles would not allow developing chukars to flap more quickly and produce more aerodynamic force (i.e., muscles are not more limiting than feathers). Previous work demonstrates that WAIR is indeed a difficult behavior for immature chukars [65–70° = maximum angle of ascent in 6–8 day chukars (Dial, 2003; Heers et al., 2016)]. Assumption (ii) is justified because even though 6–8 day old chukars use higher wingbeat frequencies during controlled aerial descents (Jackson et al., 2009), they do not appear to increase wingbeat frequency to increase aerodynamic force output and ascend steeper inclines.

*H*_1_*: feather morphology limits locomotor capacity more than muscle morphology in developing chukars*. In this case, relatively low activations of the pectoralis and supracoracoideus during simulations of WAIR would suggest that feathers, and potentially other factors such as skeletal anatomy or neurological development, limit performance more than muscle morphology. Feather limitation would be supported if the pectoralis and supracoracoideus are less-than-maximally activated when aerodynamic force requirements are increased to adult levels during simulations of WAIR. Skeletal limitations could be checked by adjusting muscle attachment sites to reflect the development of bony processes and by simulating different degrees of skeletal channelization (e.g., limits to joint ranges of motion), whereas EMG recordings on live birds are required to assess neurological development (see Tobalske et al., 2017).

In conjunction with previous work, testing these hypotheses offers insight into ontogenetic and potentially evolutionary construction of the avian body plan, by establishing how muscles, skeletal tissues, and feathers interact with each other and the environment to accomplish locomotor tasks during flightless to flight-capable transitions.

## Materials and methods

### Model development

We built three detailed musculoskeletal models of the chukar forelimb using CT scans, Maya (Autodesk; http://autodesk.com/), SIMM (Musculographics, Inc, CA; http://www.musculographics.com/), and custom scripts in Matlab software (Mathworks; https://www.mathworks.com/). Model building consisted of three basic steps: (i) using CT scan data to construct digital skeletal models comprised of rigid segments (bones) articulated by joints, (ii) adding muscles based on dissections and skeletal landmarks, and (iii) determining segment mass, center of mass, and inertial properties. This general workflow followed prior studies (e.g., Hutchinson et al., 2005; Charles et al., 2016; Otero et al., 2017) but is explained in full here.

#### Skeletal models and segment properties

We used previously constructed skeletal models, as described in Baier et al. (2013) and Heers et al. (2016). In brief, we CT or microCT scanned the carcasses of one 7–8 day old bird (“baby” model; mass 34.6 g), one 18–20 day old bird (“juvenile” model; mass 84.8 g), and one adult female (“adult” model; mass 500 g) (adult females slightly smaller than adult males, but otherwise similar; individuals in “baby” and “juvenile” age classes not distinguishable by size or sex). Mesh models of individual bones were segmented from CT scans using Amira 4.0 (Thermo Fisher Scientific; https://www.fei.com/) or Osirix 4.0 32-bit (Rosset et al., 2004), and imported into Maya to create a skeletal “puppet” (Gatesy et al., 2010) with a hierarchy of joint coordinate systems (Grood and Suntay, 1983). Joint coordinate systems for the pelvis (whole body motion) and sternal, coracosternal, shoulder, elbow, and wrist joints were defined using inertial axes and anatomical landmarks, as in Baier et al. (2013), with 3 translational and 3 rotational degrees of freedom per joint (Heers et al., 2016). Movement at any given joint (e.g., shoulder joint) caused motion of the distal bone defining the joint (e.g., humerus) as well as motion of all downstream elements (elbow joint, ulna, radius, wrist joint, manus). For full details, see (Baier et al., 2013; Heers et al., 2016).

In addition to constructing the joint hierarchy, we used CT scans (one bird per age class) to quantify distribution of mass and account for inertial effects. We imported image slices from each scan into Mimics software (Materialise, Inc.; Leuven, Belgium), used density thresholds to isolate the animal from its surroundings and to visualize the muscles and skeleton, then digitally segmented the animal into hind limb, sternum (trunk), coracoid, brachial, antebrachial, and manual segments (feathers removed; Figure S1). Using a custom script in Matlab (Allen et al., 2013) and assuming a segment density of 1,060 kg m^−3^, we then calculated the mass, center of mass, and inertial tensor for each segment, based on its volume. Finally, we scaled each segment mass so that the sum of all segment masses matched the total body mass of the specimens used to develop the models (baby segments scaled by 1.31 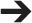 total mass 34.6 g; juvenile segments scaled by 1.03 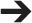 total mass 84.8 g; adult segments scaled by 1.11 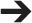 total mass 500 g). Scaled mass, center of mass, and inertial tensor for each segment were included as model parameters.

#### Muscle architecture

Following step (i), we added representations of muscle-tendon units to the skeletal models using SIMM software. A total of 30 muscles were modeled, representing all of the major muscles acting around the shoulder, elbow, and wrist joints. Individual muscle-tendon actuator dynamics were defined using Hill-type models (Zajac, 1989; Millard et al., 2013). For the “Millard Equilibrium Muscle” employed here, the force developed by a muscle and transmitted through tendon to bone (*f*^*M*^) depends on (i) the size and fiber architecture of the muscle, which dictates its maximum isometric force ($f_{o}^{M}$), (ii) the level of activation, ranging from 0 to 1 (*a*), (iii) the active force-length curve, normalized by optimal fiber length ($f^{L}\left({\tilde{l}}^{M}\right)$), (iv) the force-velocity curve, normalized by maximum contractile velocity (*f*^*V*^(ṽ^*M*^)), (v) the passive force-length curve ($f^{PE}\left({\tilde{l}}^{M}\right)$), and (vi) the pennation angle (α):

(1) $$f^{M}=\;f_{o}^{M}\left(af^{L}\left({\tilde{l}}^{M}\right)f^{V}\left({\tilde{v}}^{M}\right)+f^{PE}\left({\tilde{l}}^{M}\right)\right)\text{cos}\left(\alpha \right)$$

Calculations of maximum isometric force (i) are based on dissections and detailed below. With respect to (ii)–(vi), the Hill model used here:

- Models muscle fiber performance using an active force-length curve (iii), force-velocity curve (iv), and passive force-length curve (https://simtk.org/api_docs/opensim/api_docs/classOpenSim_1_1Millard2012EquilibriumMuscle.html)
- Models tendon elasticity using a force-length curve consisting of a non-linear toe region and a linear region with a slope set such that tendon strain is 4.9% of tendon slack length at the maximum isometric fiber force
- Assumes a constant muscle volume and varies α to maintain constant muscle height (no muscle bulging)

Parameters for (iii)–(v) were determined by fitting experimental data (see Figure 3 in Millard et al., 2013). The total force developed by muscle fibers is assumed to scale up from the force produced by a single fiber, which in turn is assumed to depend only on two time dependent states: fiber activation and fiber length. To avoid numerical singularities during simulation and reduce simulation time, minimum activation is assumed to be 0.01, maximum pennation angle is restricted (α < 84.26°), and $f^{L}\left({\tilde{l}}^{M}\right)>0$ (Millard et al., 2013). Finally, a time delay between excitation (e.g., firing of a neuron) and muscle force development is included, modeled as a first-order differential equation with activation and deactivation time constants of 10 and 40 ms, respectively (Millard et al., 2013).

Though included in our Hill model implementation, dynamic muscle properties (e.g., activation-deactivation dynamics, fiber force-velocity relationships) are not used in static simulation approaches such as OpenSim's static optimization routine (section Simulations). In addition, as detailed in the section Muscle Physiology, OpenSim's static optimization routine assumes a rigid tendon and results in artificially stretched or shortened muscle fibers for behaviors involving large ranges of motion, such as flapping. We therefore opted to not incorporate force-length relationships in our simulations. Consequently, in our simulations, muscle force development is solely dictated by the size and fiber architecture of the muscle (i.e., $f_{o}^{M}$), and the level of activation. Muscle pathways (Table S1) and fiber architecture (Table S2) were determined through dissection or measurements based on dissection (digital scales ± 0.0001 g; ImageJ ± 0.1 mm; ImageJ ± 0.1°), as detailed below.

##### Origins and insertions

**Figure 2:**
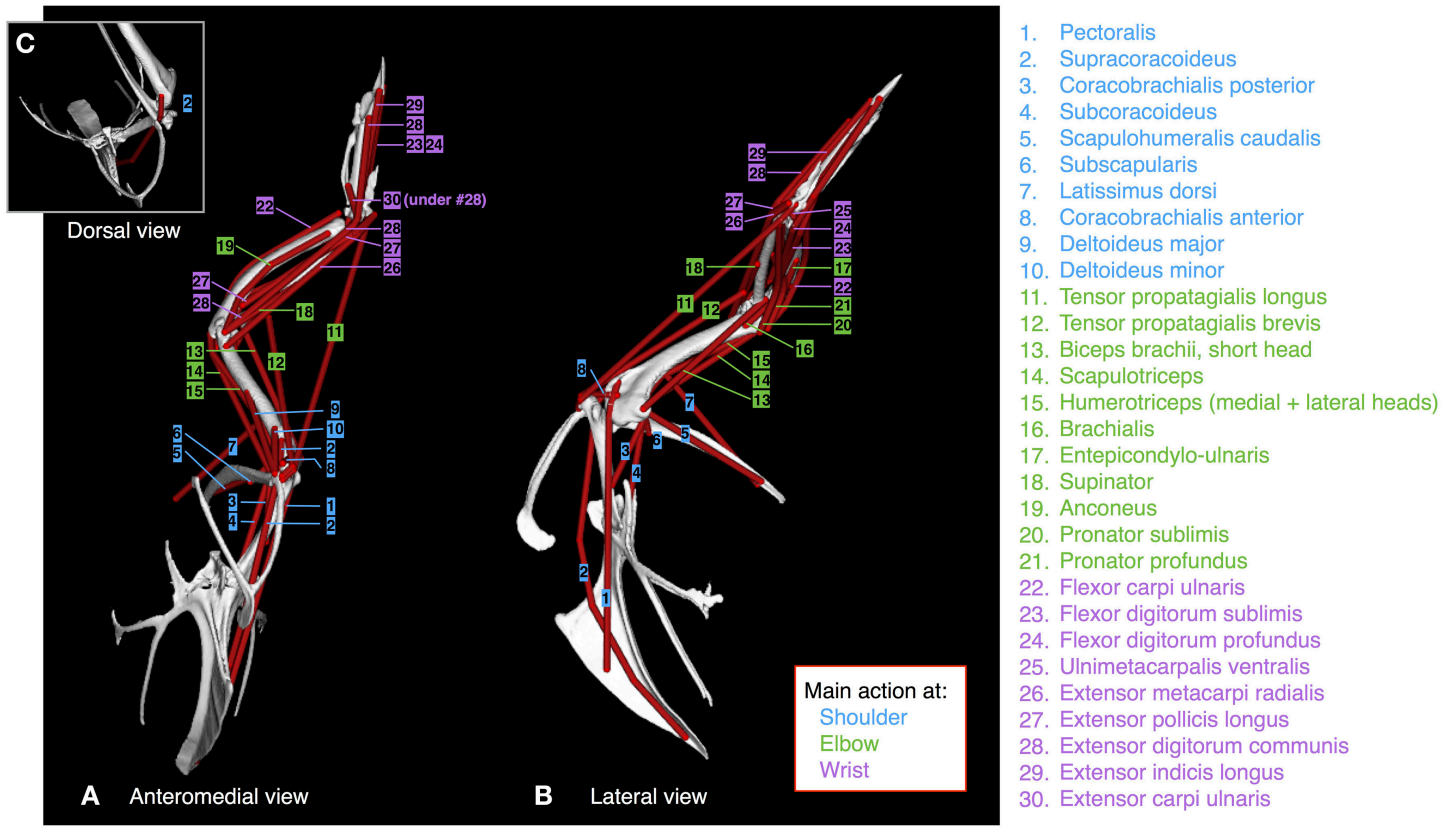
Muscles. Origins, paths, and insertions of the 30 forelimb muscles modeled. Muscles acting mainly at the shoulder are numbered in blue; muscles acting mainly at the elbow are numbered in green; muscles acting mainly at the wrist are numbered in purple. **(A)** adult model in anteromedial view, **(B)** adult model in left lateral view, **(C)** adult model in dorsal view.

We dissected 3 (baby, juvenile) or 4 (adult; two males and two females) birds per age class to obtain muscle pathways and physiological cross-sectional areas. All procedures were approved by the Institutional Animal Care and Use Committee (IACUC) at the University of Montana (specimens from Heers and Dial, 2015) or the Ethics and Welfare Committee at the Royal Veterinary College (URN 2013 1228). To define muscle pathways, we isolated each muscle during dissection, and added markers to the skeletal model in Maya at the position of each origin and insertion. Each marker was then associated with the proximal joint of the bone to which it was attached, so that the position of the marker was defined in the same coordinate space. We transferred these origin or insertion coordinates to the musculoskeletal model in SIMM to create each muscle (3D visualization is easier with Maya, hence the Maya to SIMM workflow), then added wrapping surfaces and/or via points (Delp and Loan, 2000; Delp et al., 2007) between them, where necessary, to prevent muscles from passing through bone and to maintain muscle paths that matched those of the dissected bird (Figure 2; Table S1).

Because we did not detect variation in origin and insertion positions or muscle pathways across age classes or individuals, we gave all models identical origins, insertions, and pathways, thus eliminating the possibility of having simulation outputs influenced by differences in the modeled positions of origins and insertions. To do this, we transferred origins and insertions of the adult model to the baby and juvenile models, in Maya, by (i) scaling each bone in the adult model (with associated origins and insertions) to the size of each bone in the baby or juvenile model, (ii) aligning bony landmarks, and (iii) insuring that bones made contact at joints. We scaled the length and width of each adult bone by calculating scaling factors (baby- or juvenile-to-adult ratios) based on measurements of bone lengths and widths in fresh adult, juvenile, and baby skeletons, to account for the presence of cartilage that could not be detected by CT scans and thus not visualized in our skeletal models. Origin and insertion positions were consistent with previously published data on other galliforms (Hudson and Lanzillotti, 1964).

Although many flight muscles have broad origins and/or insertions, for this analysis we modeled all muscles with a single point origin and single point insertion. To assess the effects of modeling large muscles as a single muscle with a path through the center of the volume of the muscle vs. multiple smaller muscles with different origins and paths, we compared simulations of WAIR where the pectoralis muscle was modeled as a single muscle with simulations where the pectoralis was modeled as three smaller muscles, in the adult model.

##### Optimal fiber length and average pennation angle

One additional adult bird was dissected to measure fiber lengths and pennation angles (measuring lengths and angles required removing and photographing individual muscles, which prevented accurate measurement or calculation of other parameters). Fiber length and orientation (pennation angle) are important determinants of muscle strain (changes in length) and stress (force per unit physiological cross-sectional area). To determine the optimal fiber length [taken as the fascicle length of the muscle at rest (Zajac, 1989) and assuming fiber length to be equivalent to fascicle length] and average pennation angle of each muscle, we first cut and gently removed each muscle at its origin and insertion. We then photographed the muscle from a perpendicular distance of ~60 cm with a ruler for scale, and used ImageJ (NIH; https://imagej.nih.gov/ij/) to measure the pennation angles and lengths of 1-6 muscle fibers, depending on the amount of fiber variation. For parallel-fibered muscles, we generally took one measurement, in the middle of the muscle; for pennate muscles, we took up to three evenly spaced measurements on either side of the central tendon. Averages were calculated for muscles with multiple measurements. Pennation angles were qualitatively consistent with previously published illustrations of forelimb muscles in galliform birds (Hudson and Lanzillotti, 1964).

Given that the adult bird on which we measured fiber lengths and pennation angles (“measured” in Equation 2) was slightly more muscular than the adult birds we used to construct the musculoskeletal model (“modeled” in Equation 2; total forelimb muscle mass of measured bird was 1.18 times greater than average of modeled birds), we assumed that length is proportional to mass^1/3^ and scaled the modeled fiber lengths to ~95% of their measured value, based on the following equation:

(2) $$\begin{array}{l} \text{fiber length},\text{ modeled }=\\ \;(\text{fiber length},\text{ measured}){\left(\frac{\text{total forelimb muscle mass},\text{ modeled}}{\text{total forelimb muscle mass},\text{ measured}}\right)}^{1/3} \end{array}$$

where total muscle mass is the sum of the masses of the major forelimb muscle-tendon units (pectoralis, supracoracoideus, coracobrachialis posterior, latissimus dorsi, scapulohumeralis caudalis, deltoideus major, tensor propatagialis brevis, biceps brachii, triceps brachii, all antebrachial muscles).

For baby and juvenile birds, we were unable to clearly see individual fascicles, either with the naked eye or under a dissecting microscope. We therefore assumed that across age classes, pennation angles would be constant, and fiber lengths would be proportional to muscle lengths:

(3) $$\begin{array}{l} \text{baby or juvenile fiber length }=\\ \;(\text{adult fiber length},\text{ modeled})\left(\frac{\text{MTU length},\text{ baby or juvenile model}}{\text{MTU length},\text{ adult model}}\right) \end{array}$$

where MTU length is the muscle-tendon unit length (calculated in SIMM) for the muscle of the fiber in question, averaged over one wingbeat cycle of WAIR (average kinematics during 60–65° WAIR for baby and juvenile, 70–80° WAIR for adult, to standardize for level of effort—see Heers et al., 2016).

##### Maximum (isometric) muscle force

Following previous studies (Hutchinson et al., 2015 and references therein), we assumed that for each muscle, maximum isometric force (*F*_*max*_, N) is proportional to the physiological cross-sectional area of the muscle (*A*_*phys*_, m^2^):

(4) $$\begin{array}{rcl} A_{phys}\; & = & \;m_{musc}\cos\left(\alpha \right)L_{f_{o}}^{-1}d^{-1} \end{array}$$

(5) $$F_{max}=(3.0\times 10^{5}\text{ N }{\text{m}}^{\text{-2}})(A_{phys})$$

where *m*_*musc*_ is the mass of the muscle in question [kg; average mass for 3 (baby, juvenile) or 4 (adult; 2 male and 2 female) birds (data from Heers and Dial, 2015; supplemented by additional dissection for previously unmeasured muscles)], α is the average pennation angle (radians), *L*_*f*_*o*__ is the optimal fiber length (m), *d* is muscle density (1,060 kg m^−3^ Mendez and Keys, 1960; Brown et al., 2003; Hutchinson et al., 2015), and 3.0 × 10^5^ N m^−2^ is isometric stress under maximal muscle activation (Medler, 2002; Nelson et al., 2004).

To check the validity of these calculations, we compared *F*_*max*_ of the pectoralis (main downstroke muscle) with force production measured in pigeons. Very few data are available on force production by avian flight muscles, partially because there is no direct way to measure force production by these muscles (see Biewener, 2011). Previous studies using calibrated strain gauges to estimate force production by the pectoralis in flying pigeons have reported forces ranging from 18 to 26 N (Dial and Biewener, 1993; body mass 301–314 g, pectoralis mass 28.0–33.9 g) to >120 N (Soman et al., 2005; body mass 522–593 g, pectoralis mass 46.6–55.8 g). In the smaller pigeons, pectoralis forces obtained during flight were well below the isometric force estimated by supramaximally stimulating the muscle in anesthetized birds (18–26 N vs. 67 N). Assuming a similar relationship, maximal isometric force in the larger pigeons should have been ~260–480 N. We calculated a maximal isometric force of 231 N for the pectoralis muscle of our adult chukar (body mass 500 g, pectoralis mass 39.5 g). This value is intermediate between the pigeons' isometric forces, as would be expected given that the mass of the chukar pectoralis is intermediate between that of the two groups of pigeons. Also consistent with our work, (Yang et al., 2015) calculated a maximal isometric muscle force of 335 N for the pectoralis of a Golden Pheasant (*Chrysolophus pictus*), which is slightly smaller than our chukar (average body mass 422 g, average pectoralis mass 28.8 g) but has proportionally shorter muscle fibers and therefore a larger *A*_*phys*_.

##### Tendon slack length

Tendon slack length (*L*_*ts*_) is defined as the length beyond which the tendons associated with a muscle begin resisting stretch and producing force. This parameter essentially determines how much of the total force that a muscle-tendon unit produces is produced actively, by the muscle contracting, vs. passively, by the tendon(s) (series elastic component) being stretched. We used the algorithm provided by Manal and Buchanan (2004) to calculate a tendon slack length *L*_*ts*_ for each muscle. This algorithm requires knowledge of the minimum and maximum muscle-tendon unit lengths (*L*_*mt*_; length of muscle (*L*_*m*_) + tendon(s) (*L*_*t*_)) across a range of joint motions, the average pennation angle of the muscle fibers (α), and the normalized fiber lengths (*L*_*f*~;_ = instantaneous fiber length (*L*_*f*_) / optimal fiber length (*L*_*f*_*o*__) at the minimum and maximum values of *L*_*mt*_) (Figure S2):

(6) $$L_{f^{∼}\;}=\frac{L_{f}}{L_{f_{o}}}\to L_{f}=(L_{f^{∼}})(L_{f_{o}})$$

(7) $$\cos(\alpha )=\frac{L_{m}}{L_{f}}=\frac{L_{m}}{\left(L_{f^{∼}\;})(L_{f_{o}}\right)}\to L_{m}=\cos(\alpha )(L_{f^{∼}})(L_{f_{o}})$$

(8) $$\begin{array}{c} \begin{array}{c} \begin{array}{c} L_{ts}\propto L_{mt}-L_{m}=L_{mt}-\cos(\alpha )(L_{f^{∼}})(L_{f_{o}}) \end{array} \end{array} \end{array}$$

For the minimum and maximum values of *L*_*mt*_, we used the minimum and maximum lengths associated with maximal effort WAIR or ascending flight (= full range of motion; kinematics from Baier et al., 2013; Heers et al., 2016; values of *L*_*mt*_ calculated in SIMM), and assumed that the normalized fiber lengths associated with these minimum and maximum muscle-tendon unit lengths were 0.5 and 1.5, respectively (Manal and Buchanan, 2004).

### Experimental data

#### Skeletal kinematics

Flapping kinematics for WAIR in each age class were taken from Heers et al. (2016) and Baier et al. (2013) (Videos S1–S3). These studies quantified translations and rotations for the “pelvis” (whole body motion), sternum, coracosternal, and all major forelimb joints (shoulder, elbow, wrist), averaged over the downstroke and upstroke of two (adults; two trials per bird) or three (baby, juvenile; one trial per bird) birds. As in Heers et al. (2016), we chose to use averaged kinematics rather than the kinematics of one individual for one trial, due to the difficulty of using XROMM to measure skeletal kinematics in juvenile birds.

#### Aerodynamic forces

**Figure 3:**
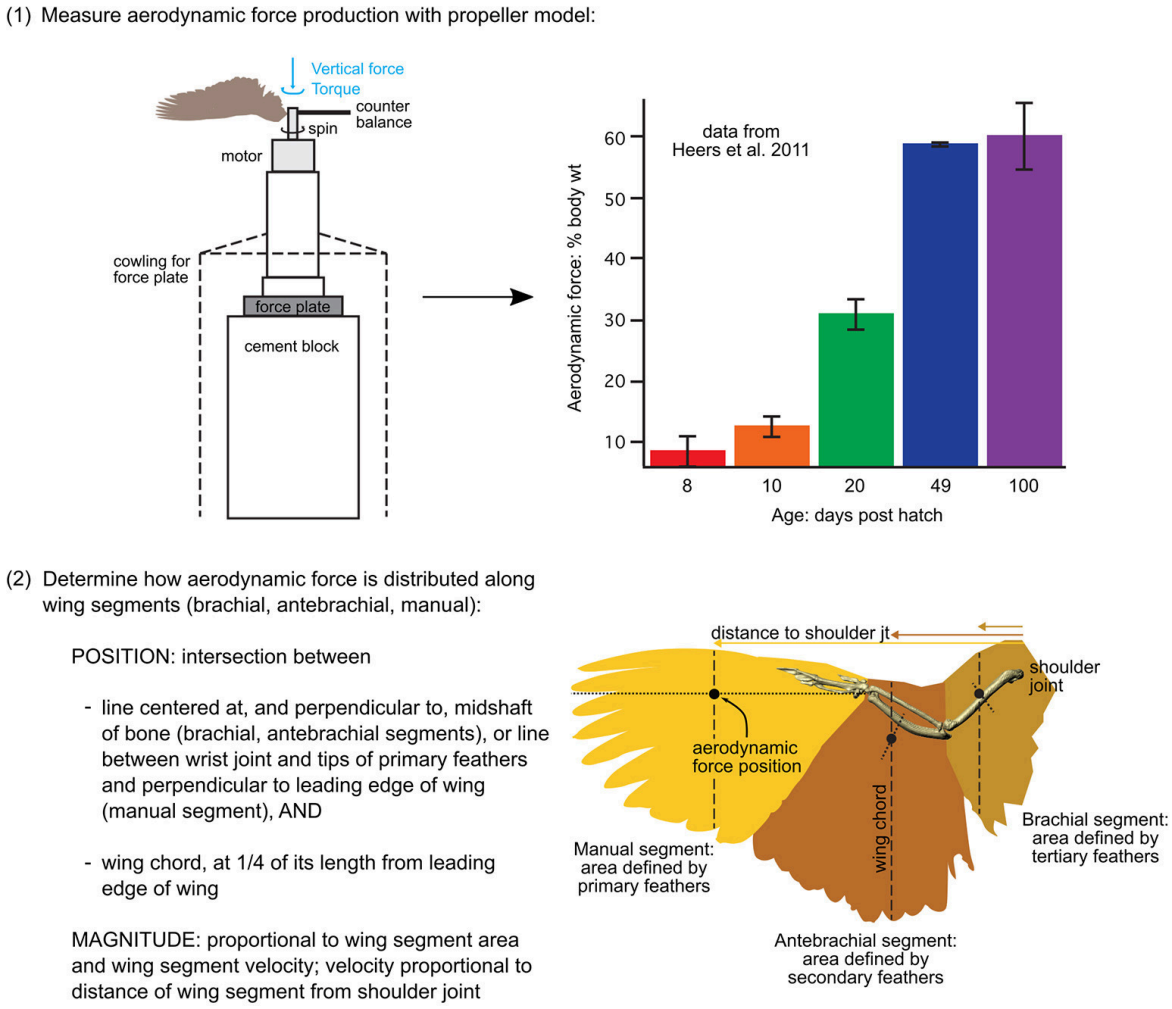
Aerodynamic force calculations. Stepwise procedure showing how the magnitudes and positions of aerodynamic forces were calculated for each model. Data in the graph is from Heers et al. (2011); the drawing of the propeller apparatus is modified from Crandell and Tobalske (2011). Resulting model inputs are shown in Table S3.

Incorporating aerodynamic forces into the models required two basic steps: (1) empirically measuring total force production, and (2) estimating the distribution (magnitude and position) of forces along the wing (steps summarized in Figure 3).

(1) To measure total aerodynamic force production during WAIR, we dried wings in a mid-downstroke posture and spun them like a propeller, attached to a force plate via a motor, to measure both lift and drag [two wings per age class; data from (Heers et al., 2011) consistent with PIV measurements on live birds (Tobalske and Dial, 2007)]. To scale the measured forces to the sizes of our model animals, we multiplied the weight of each model animal (baby, juvenile, adult) by the aerodynamic force measured for its age class, expressed as a proportion of body weight.

(2) To estimate how (scaled) aerodynamic force was distributed along each wing segment (brachial, antebrachial, manual), we calculated the proportion of the resultant force that would be produced by each wing segment:

(9) $$\text{Resultant force}=0.5pC_{R}S\left({\left(\Omega r\right)}^{2}+V_{T}{}^{2}\right)\propto \;S\left({\left(\Omega r\right)}^{2}+V_{T}{}^{2}\right)$$

(10) $$\begin{array}{r} \text{Vertical force}=\left(\text{resultant force}\right)\left(\text{sin}\left(\text{resultant force angle}\right)\right) \end{array}$$

(11) Horizontal force ​= ​(resultant force)(cos(resultant force angle))

where the resultant force (N) is the total aerodynamic force produced by the wing segment (brachial, antebrachial, or manus) at mid-downstroke, *p* is air density (1.07 kg m^−3^ in Missoula, Montana; where experiments were done), *C*_*R*_ is the coefficient of the resultant force based on force plate measurements (amount of force produced per unit surface area and velocity^2^, at a given air density; data from Heers et al., 2011, *n* = 2 individuals per age class), *S* is the surface area of the wing segment (m^2^), measured in ImageJ from photographs (photos from Heers et al., 2011), Ω is the angular velocity of the wing, based on high speed video of chukars performing WAIR (data from Heers et al., 2011), *r* is the “length” of the wing segment (distance between shoulder joint and center of wing segment at mid-downstroke), and *V*_*T*_ is the translational velocity of the animal during WAIR (data from Jackson et al., 2009).

Note that Equation (9) assumes angular (flapping) and translational (running) velocities are perpendicular to one another—this is a conservative estimation of total wing velocity that can be applied to future models. Any resulting discrepancies between the total aerodynamic force calculated using (Equations 9–11) and the total aerodynamic force measured by the force plate (Heers et al., 2011) were rectified by scaling.

Following steps (1) and (2), we positioned the aerodynamic force associated with each wing segment in the proximodistal center of the segment (at *r* in Equation 9), in the plane of the wing (dorsal surface of humerus, ulna + radius, or manus in mid-downstroke position), and at the quarter chord length position (Figure 3; Table S3), because aerodynamic theory predicts that the magnitude of aerodynamic force production should be greatest at this position (e.g., Anderson, 2017).

To check the validity of this workflow, we compared the distributions of aerodynamic force in our adult model with previous work using pressure sensors to determine force production along the wing of a flying pigeon (Usherwood et al., 2005). Modeled and measured forces were consistent. In the pigeon, aerodynamic force produced at the eighth primary feather (feather P8; approximately equivalent to manus segment) was 2–3 times greater than force produced by more proximal secondary feathers (feathers S1, S7; approximately equivalent to antebrachial segment). In our chukar model, aerodynamic force produced by the manual segment was 4.4 times greater than force produced by the antebrachial segment. Compared to the pigeon, chukars engaged in WAIR flap at higher angular velocities [higher wingbeat frequencies; 18.7 Hz (Jackson et al., 2009) vs. 8.0 Hz in the pigeon (Usherwood et al., 2005)] and move (i.e., run) at lower translational velocities [1.57 m s^−1^ (Jackson et al., 2009) vs. 4.46 m s^−1^ in the flying pigeon (Usherwood et al., 2005)]. Based on these velocity differences, and assuming that angular velocity is proportional to wingbeat frequency and wing segments are roughly proportional in chukars and pigeons, we would expect the manual-to-antebrachial force ratio to be ~2 times higher in chukars than in pigeons (aerodynamic force $\propto {\left(\Omega r\right)}^{2}+{V_{T}}^{2}$; see Equation 9).

### Simulations

Following musculoskeletal model construction, we imported our models and experimental data into OpenSim (Delp et al., 2007) and used the built-in inverse dynamics and static optimization algorithms to estimate patterns of muscle activation and force development. For a given set of kinematics and external loads (in this case, aerodynamic forces), inverse dynamics determines the net joint moments required to produce the motion. Static optimization then resolves net moments into individual muscle moments (muscle force times muscle moment arm) at each time step by minimizing the sum of squared muscle activations. We made three adjustments before running simulations:

#### Kinematic inputs

Model joints must be constrained either by skeletal geometry and ligaments or by muscles. Previous studies have incorporated skeletal and ligament constraints by limiting various rotations, or by defining bone translation as a function of bone rotation, such that muscle activity only affects unconstrained rotations and/or translations. Rather than characterizing soft-tissue and skeletal constraints that limit translation in each wing joint—which would have been extremely challenging given the complexity of the avian forelimb—we instead initially constrained joint translations to match the experimentally observed values. Thus, only bone rotations were driven by muscle activity.

Because our aim was to examine how the avian wing functions through ontogeny, we focused on the three main wing joints (shoulder, elbow, wrist); for other body joints (coracosternal, sternal, pelvis/whole body), we initially prescribed all translations and rotations to match experimental data so that the animal's body would be moving at the correct speed and orientation without requiring additional muscles in the model to drive those movements. However, simulations with the prescribed motions and simulations containing only rotations at the shoulder, elbow, and wrist (i.e., shoulder, elbow, and wrist translations, and all other non-wing joint motions, “locked”) yielded very similar results; thus, to simplify comparisons, here we report on simulations using only wing rotations. Kinematics were low-pass filtered at 53–56 Hz.

Reserve actuators, which contribute joint moments if model muscles are not strong enough (Hicks et al., 2015; Rankin et al., 2016), were set at an optimal force representing 50% of the maximum moment (based on inverse dynamics) at a joint in a given direction, for each model (Table S4); smaller reserve actuators resulted in simulation failure. Residual actuators were never used, since the body was “locked” into position.

#### Aerodynamic input

To account for the fact that our propeller models only measured aerodynamic force production at mid-downstroke, we assumed an inactive upstroke (no aerodynamic force production) and applied our measured aerodynamic forces to the middle quarter of the kinematic downstroke, then tapered force to zero using a linear interpolation in both directions. Thus, in our models, the beginning of the downstroke—defined as the point at which the tip of the manus began moving closer to the sternum—produced no aerodynamic force, aerodynamic force increased steadily up to mid-downstroke and remained constant through the middle quarter of downstroke, then decreased steadily to zero at the end of downstroke and remained at zero through the entire upstroke. Although this was a simplifying assumption, newly developed techniques for measuring aerodynamic force production *in vivo* confirm that aerodynamic force production peaks roughly in mid-downstroke and tapers steadily to zero or nearly zero at the beginning and end of downstroke (Lentink et al., 2015; Figure S3).

In addition to simulating 65° WAIR under *in vivo* conditions, we simulated 65° WAIR for the baby and juvenile models under five theoretical conditions (Table 1) designed to test our two hypotheses by assessing whether the pectoralis and supracoracoideus muscles of baby and juvenile birds were capable of flapping more effective, adult-like wings. Treatments 1 and 2 were designed to represent a baby or juvenile bird flapping a wing with adult-like feathers (adult value of aerodynamic force, in terms of percent body weight and lift-to-drag ratio; no change in wing size or position of aerodynamic force). Treatments 2, 3, and 4 were designed to represent a baby or juvenile bird flapping a wing with adult kinematics (adult wingbeat frequency and adult rotations at the shoulder, elbow, and wrist; all other kinematics unchanged) and different amounts of aerodynamic force (*in vivo* or adult magnitudes). Finally, Treatments 4 and 5 were designed to represent a baby, juvenile, or adult bird flapping a wing without producing any aerodynamic force, to account for inertial properties.

**Table 1:** Kinematic and aerodynamic manipulations.

**Treatment**	**Musculoskeletal morphology**	**Magnitude of aerodynamic force**	**Kinematics**
1	Baby or Juvenile musculoskeletal morphology (unchanged)	Adult aerodynamic force	Baby or Juvenile kinematics (unchanged)
2		Adult aerodynamic force	Adult kinematics
3		Baby or Juvenile aerodynamic force (unchanged)	Adult kinematics
4		No aerodynamic force	Adult kinematics
5		No aerodynamic force	Baby or Juvenile kinematics (unchanged)

*Adult aerodynamic force (Treatments 1, 2): adult value of aerodynamic force, in terms of percent body weight and lift-to-drag ratio; no change in wing size or position of aerodynamic force. Adult kinematics (Treatments 2, 3, 4): adult wingbeat frequency and adult rotations at the shoulder, elbow, and wrist; all other kinematics unchanged. No aerodynamic force (Treatments 4, 5): accounts for inertial properties*.

#### Muscle physiology

OpenSim's static optimization routine assumes that tendons are rigid and do not stretch. For behaviors involving a large range of motion, such as flapping, this is problematic because all length changes must unrealistically occur in the muscle fibers, resulting in overly stretched or compressed fibers less capable of generating force. Our preliminary simulations using a rigid tendon resulted in most of the joint moment being contributed by reserve actuators, rather than muscles, for all joints and all models. To remove errors imparted by the assumption of tendon rigidity, we ignored muscle force-length relationships (muscle physiology turned “off” in OpenSim) and re-ran simulations. This did not affect the timing of muscle activations and substantially reduced the moments contributed by reserve actuators, but did not eliminate them completely: reserve actuators still contributed >10% of the total joint moment (<10% desirable; see Hicks et al., 2015; Rankin et al., 2016) (Figure S4; Table S5).

Additional sensitivity analyses where joint locations, muscle geometries, flapping kinematics, and aerodynamic force locations were adjusted within the range observed among chukars or within the range calculated under different assumptions (e.g., joint location determined by joint anatomy vs. joint location determined by kinematics) could not eliminate the need for the reserve moments (Table S6). This suggested that the limitation was inherent to using a static approach, which likely (and unsurprisingly) cannot completely characterize dynamic muscle function during flapping. Static approaches are nonetheless a valuable first start that more dynamic approaches can build upon, and we proceeded with this in mind.

Previous studies have suggested that the storage and release of elastic energy by tendons and ligaments likely plays an important role in the high frequency flapping kinematics of birds (e.g., Tobalske and Biewener, 2008). These dynamic contributions cannot be captured by static optimization. Given that peak joint moments occurred at wing turnaround (upstroke-downstroke and downstroke-upstroke transitions), we hypothesized that the remaining moments contributed by reserve actuators most likely represent moments that would be contributed by elastic tendons / ligaments that are stretched during downstroke or upstroke and then spring back into place at wing turnaround. More complex, dynamic simulations can test this idea, but are beyond the scope of this paper. Regardless of the limitations of a static approach, it is very unlikely that our ontogenetic comparisons would change with dynamic simulations, because all models were constructed the same way and thus faced the same limitations associated with static simulations.

## Results

### Model validation

To validate our model and simulations, we compared the simulated muscle activations, lengths, and forces with empirical data—when available—from live birds. Simulations with the pectoralis modeled as a single muscle following a path through the center of the volume of the muscle vs. simulations with the pectoralis modeled as three smaller muscles—to account for its broad origin (see methods)—did not alter the timing of pectoralis activation but did slightly reduce its average level of activation, most likely due to the greater range of moment arm values (Figure S5). This and other modeling decisions (Table S6) would not affect our ontogenetic comparisons, so for the purposes of this study we herein report results with all muscles modeled as single muscles.

#### Timing of muscle activations, length changes, and force development

**Figure 4:**
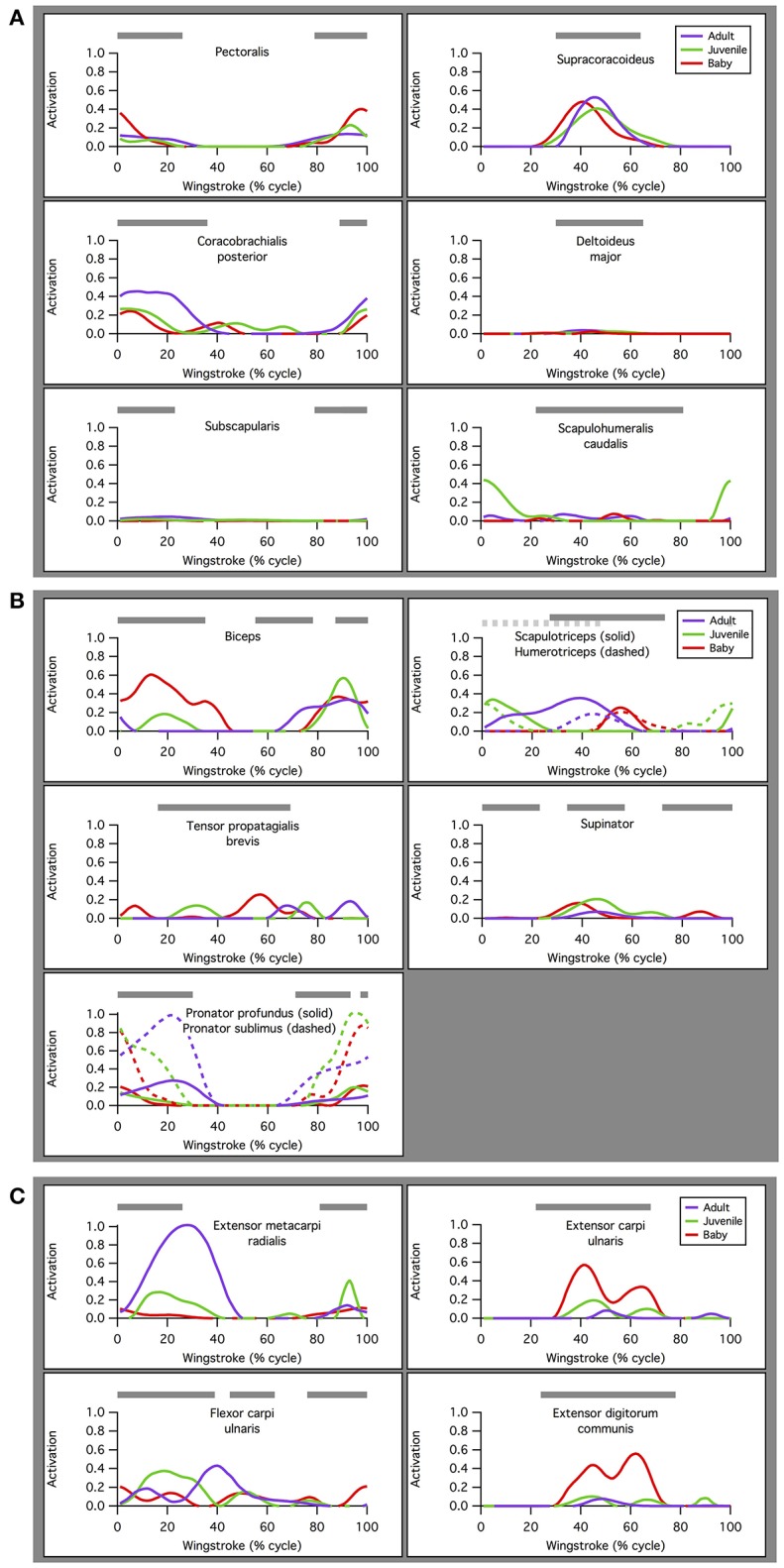
Muscle activations. Simulated patterns of muscle activation during wing-assisted incline running on 65° inclines (red, green, and purple lines) are broadly similar to the timing of muscle activity during ascending flight in pigeons (gray bars, from Dial, 1992a); for explanation of exceptions, see text. Here, the upstroke-downstroke transition is defined as the point at which the tip of the manus begins moving downward, and the downstroke-upstroke transition as the point at which the tip of the manus begins moving upward. **(A)** Muscles acting at the shoulder joint. **(B)** Muscles acting at the elbow joint. **(C)** Muscles acting at the wrist joint.

Our simulations of WAIR on 65° slopes yielded patterns of muscle activation that were broadly consistent with patterns of EMG activity in flying birds (Figure 4). EMG data are only available for the pectoralis muscle during WAIR [pigeons (Jackson et al., 2011b); chukars (Tobalske et al., 2017)]. However, ascending flight provides a reasonable comparison because it has a similar body trajectory to WAIR and comparable flapping kinematics [chukars show similar directions of movement, e.g., flexion vs. extension, but greater ranges of motion, in flight vs. WAIR (Baier et al., 2013)]. Compared to muscle activity in pigeons during ascending flight (Dial, 1992b), our simulated activations of most forelimb muscles (14 out of 16 for which comparisons were possible) either (a) qualitatively matched EMG signals recorded *in vivo*, with no or low offset in timing [pectoralis, supracoracoideus, coracobrachialis posterior, deltoideus major, subscapularis, pronator, supinator (second peak of activity *in vivo* very low magnitude), extensor metacarpi radialis, extensor carpi ulnaris, extensor digitorum communis], or (b) differed only moderately (biceps brachii—simulations did not capture second EMG burst; triceps—summed activity of scapulo- and humerotriceps identical to *in vivo* data, but model did not discriminate between different heads; flexor carpi ulnaris—constant but low activity). For the scapulohumeralis caudalis muscle, our simulations predicted an additional peak of activity in the juvenile model. For the tensor propatagialis brevis, our simulations differed substantially across age classes and occurred at different points in the stroke cycle.

**Figure 5:**
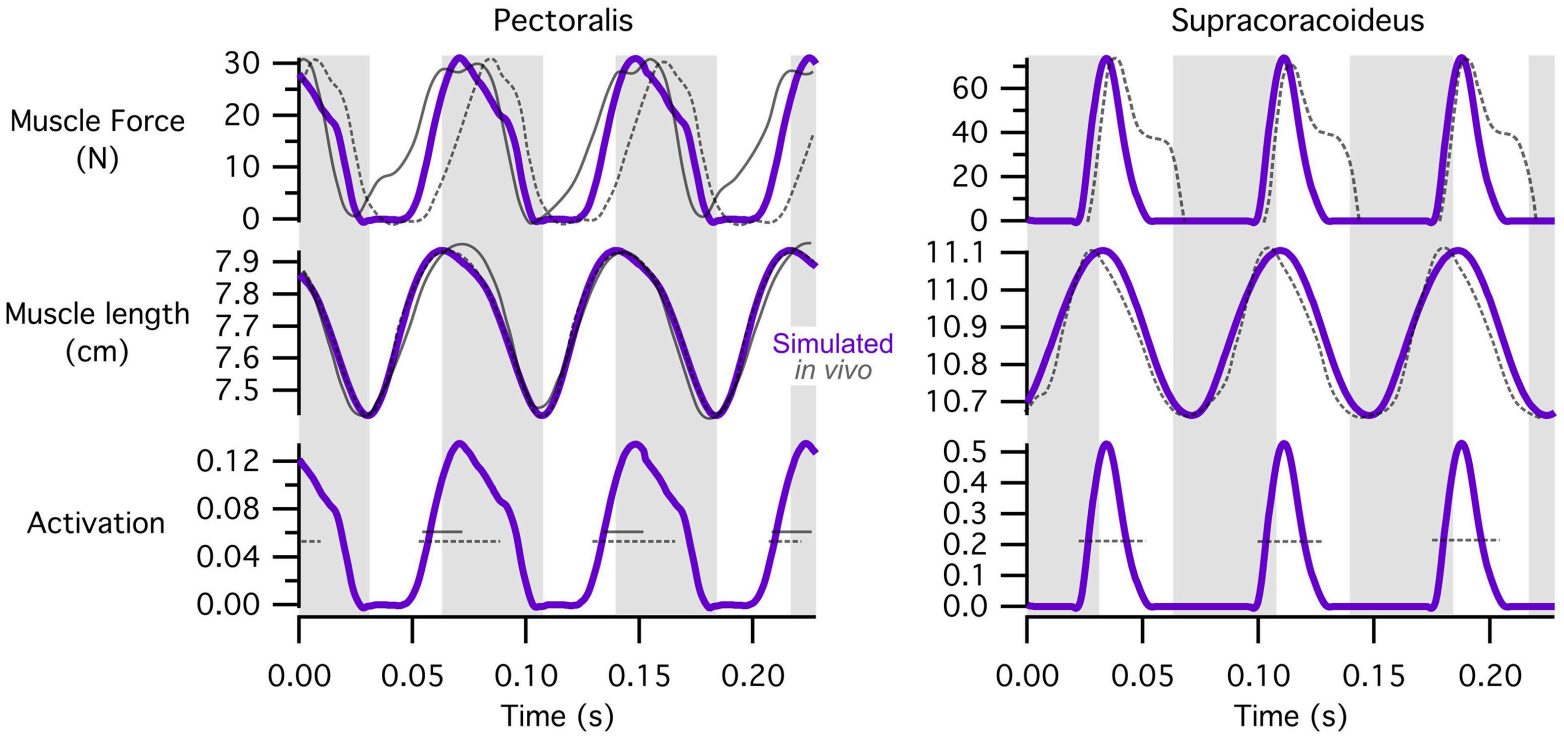
Patterns of muscle activation, changes in length, and force development. Simulation estimates of muscle activation, shortening vs. lengthening, and force development are qualitatively similar to patterns reported for flying (Tobalske and Biewener, 2008) or flap-running (Jackson et al., 2011a) pigeons. Simulated chukar data is in purple; *in vivo/in vitro* pigeon data is in gray; solid lines, flap-running on 65° inclines; dashed lines, ascending flight. Gray regions indicate simulated shortening of the pectoralis muscle. Lengths represent muscle-tendon lengths in chukars. Pigeon forces and lengths are expressed as stresses and strains, respectively; pigeon muscle “activity” is actually muscle excitation (EMG), which precedes activation; axes for pigeon data are not shown. All comparisons are based on adult birds.

For the pectoralis and supracoracoideus (data not available for other muscles), in addition to similarities in the timing of muscle activation, simulated patterns of muscle-tendon shortening vs. lengthening and force development were qualitatively similar to patterns reported for muscle fibers in flying (Tobalske and Biewener, 2008) or flap-running (Jackson et al., 2011a) pigeons (Figure 5). For both the pectoralis and supracoracoideus, simulated muscle activation began mid-muscle-lengthening, consistent with *in vivo* excitations. The pectoralis and supracoracoideus shortened and lengthened in opposition to one another, and both muscles began developing force while lengthening, as *in vivo*. Peak force occurred when the muscle-tendon units were relatively long.

#### Magnitude of muscle activations

**Figure 6:**
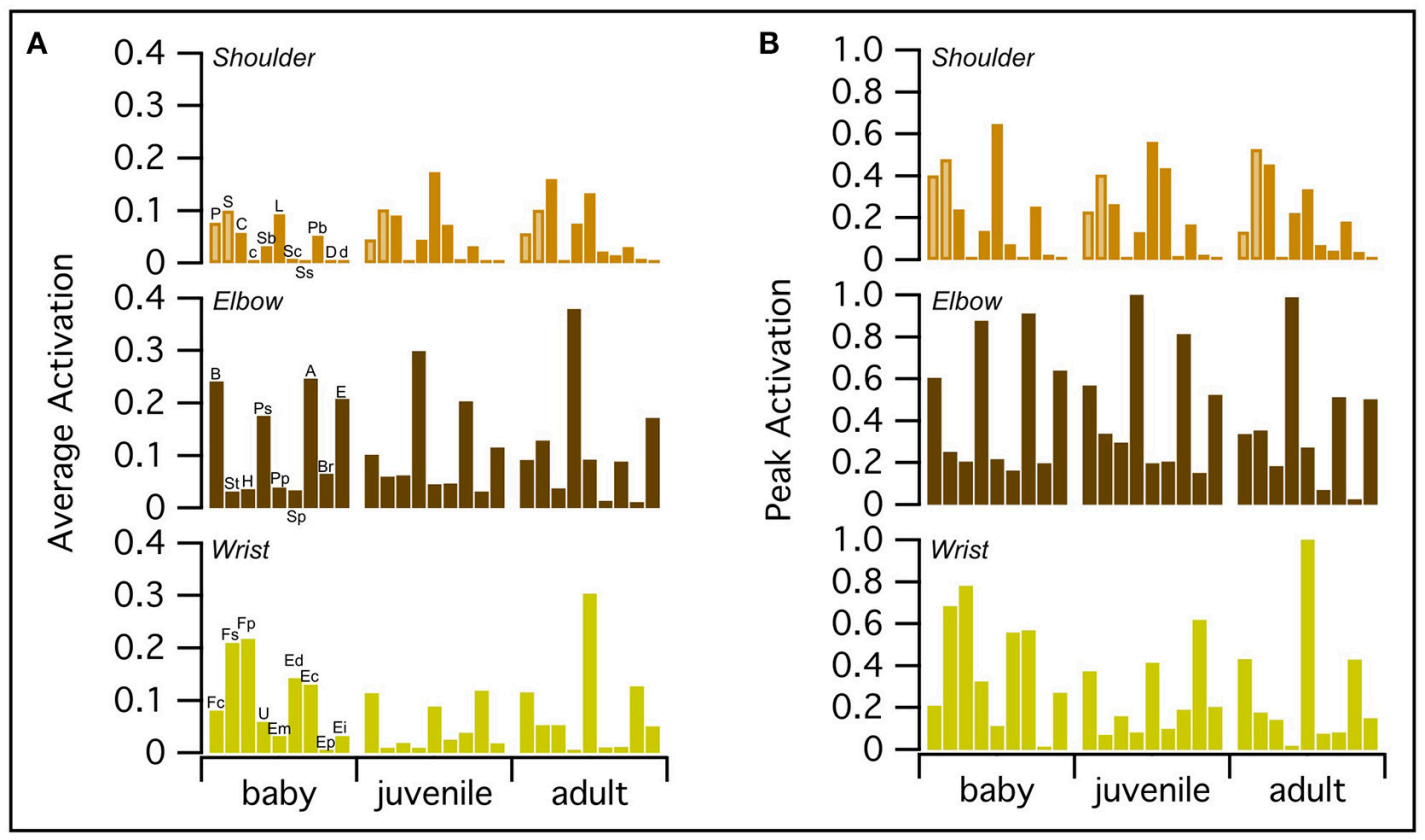
Peak and average muscle activations. On average, baby, juvenile, and adult chukars have similar levels of muscle activation **(A)**, though the baby chukar has slightly higher peak activations than the juvenile, which has slightly higher peak activations than the adult **(B)**. For all age classes, elbow and wrist muscles have higher average and/or peak activations than shoulder muscles, which have relatively low activations (generally <0.5) (Table S7). Each bar represents one muscle. The pectoralis and supracoracoideus are distinguished by lighter brown coloring in the top rows. P, Pectoralis; S, Supracoracoideus; C, Coracobrachialis posterior; c, Coracobrachialis anterior; Sb, Subcoracoideus; L, Latissimus dorsi; Sc, Scapulohumeralis caudalis; Ss, Subscapularis; Pb, Propatagialis brevis; D, Deltoideus major; d, Deltoideus minor; B, Biceps brachii; St, Scapulotriceps; H, Humerotriceps; Ps, Pronator sublimis; Pp, Pronator profundus; Sp, Supinator; A, Anconeus; Br, Brachialis; E, Entepicondyloulnaris; Fc, Flexor carpi ulnaris; Fs, Flexor digitorum sublimis; Fp, Flexor digitorum profundus; U, Ulnimetacarpalis ventralis; Em, Extensor metacarpi radialis; Ed, Extensor digitorum communis; Ec, Extensor carpi ulnaris; Ep, Extensor pollicis longus; Ei, Extensor indicis longus.

WAIR is one of the least power-demanding flapping behaviors for adult birds [pectoralis power output <60 W kg^−1^ compared to ~60–190 W kg^−1^ for various modes of flight (Jackson et al., 2011b)]. Thus, we expected activation of the flight muscles to be relatively low for the power-generating (shoulder) muscles in the adult model. On average, all three models had similar levels of muscle activation, though the baby had slightly higher peak activations than the juvenile, which had slightly higher peak activations than the adult (Figure 6; Table S7). For all age classes, elbow and wrist muscles had higher average and/or peak activations than shoulder muscles, which, consistent with expectations for the adult model, had relatively low activations (generally <0.5).

### Model predictions of muscle function

#### Muscle moment arms

Moment arms indicate how muscle force is transformed into limb motion—which joint rotations a muscle should cause (or oppose) if activated at a particular point in a locomotor sequence. To estimate each muscle's maximum movement-generating potential at the shoulder, elbow, and/or wrist joints, we multiplied the maximum isometric force (Table S2) by moment arm estimates at every 1% of the stroke cycle. Almost all (~90%) muscle actions were similar across age classes (Table 2). Differences in muscle action across age classes occurred in 7 (out of 30) muscles. At the shoulder, the scapulohumeralis caudalis had high potential to contribute to humeral elevation in the adult model but little capacity to do so in the baby and juvenile models. This muscle could also contribute to humeral protraction in the adult and baby models, but not in the juvenile model. At the elbow, the pronator had capacity for antebrachial supination in the adult and baby but not the juvenile model, while the humerotriceps had capacity for antebrachial supination in the baby and juvenile but not the adult. Finally, at the wrist, the ulnimetacarpalis ventralis had potential to flex the wrist in the juvenile but not the adult or baby models, and to supinate the manus in the adult and baby models, but not the juvenile. The extensor metacarpi radialis could contribute to manual abduction in the adult and baby models but not the juvenile, the flexor carpi ulnaris could supinate the manus in the adult and baby but not the juvenile model, and the flexor digitorum could supinate the manus in the baby but not the adult or juvenile models. All other potential actions were similar across age classes.

**Table 2:** **(A–C)** Muscle moment arms and potential functions.

**Rotation**	**Age class**			**Ontogenetic differences? (< 5% in one model and >20% in another)**
	**Baby**	**Juvenile**	**Adult**	
**(A) SHOULDER MUSCLES**				
Depression	**Pectoralis**	**Pectoralis**	**Pectoralis**	
Elevation	**Supracoracoideus** Scapulohumeralis caudalis Latissimus dorsi Deltoideus major Scapulotriceps	**Supracoracoideus** Scapulohumeralis caudalis Latissimus dorsi Deltoideus major Scapulotriceps	**Supracoracoideus** Scapulohumeralis caudalis Latissimus dorsi Deltoideus major Scapulotriceps	Scapulohumeralis caudalis
Retraction	**Pectoralis** Coracobrachialis posterior Scapulohumeralis caudalis Latissimus dorsi Subcoracoideus Scapulotrceps	**Pectoralis** Coracobrachialis posterior Scapulohumeralis caudalis Latissimus dorsi Subcoracoideus Scapulotriceps	**Pectoralis** Coracobrachialis posterior Scapulohumeralis caudalis Latissimus dorsi Subcoracoideus Scapulotriceps	
Protraction	**Supracoracoideus** Scapulohumeralis caudalis Pectoralis Propatagialis brevis	**Supracoracoideus** Scapulohumeralis caudalis Pectoralis Propatagialis brevis	**Supracoracoideus** Scapulohumeralis caudalis Pectoralis Propatagialis brevis	Scapulohumeralis caudalis
Pronation	**Pectoralis** Scapulohumeralis caudalis	**Pectoralis** Scapulohumeralis caudalis	**Pectoralis** Scapulohumeralis caudalis	
Supination	**Supracoracoideus** x Propatagialis brevis Coracobrachialis posterior Subcoracoideus	**Supracoracoideus** Pectoralis Propatagialis brevis Coracobrachialis posterior Subcoracoideus	**Supracoracoideus** x Propatagialis brevis Coracobrachialis posterior x	
**(B) ELBOW MUSCLES**				
Flexion	Propatagialis brevis Biceps brachii Pronator Extensor metacarpi radialis	Propatagialis brevis Biceps brachii Pronator Extensor metacarpi radialis	Propatagialis brevis Biceps brachii Pronator Extensor metacarpi radialis	
Extension	Humerotriceps Scapulotriceps Flexor carpi ulnaris Anconeus	Humerotriceps Scapulotriceps Flexor carpi ulnaris x	Humerotriceps Scapulotriceps Flexor carpi ulnaris x	
Adduction	Pronator Humerotriceps Entepicondyloulnaris Flexor carpi ulnaris Biceps brachii Scapulotriceps Propatagialis brevis	Pronator Humerotriceps Entepicondyloulnaris Flexor carpi ulnaris Biceps brachii Scapulotriceps Propatagialis brevis	Pronator Humerotriceps Entepicondyloulnaris Flexor carpi ulnaris Biceps brachii Scapulotriceps x	
Abduction	Anconeus Extensor metacarpi radialis Propatagialis brevis Supinator Extensor carpi ulnaris Extensor digitorum communis	Anconeus Extensor metacarpi radialis Propatagialis brevis Supinator Extensor carpi ulnaris Extensor digitorum communis	Anconeus Extensor metacarpi radialis Propatagialis brevis Supinator Extensor carpi ulnaris Extensor digitorum communis	
Pronation	**Propatagialis brevis** Anconeus Biceps brachii Humerotriceps Scapulotriceps Extensor metacarpi radialis	**Propatagialis brevis** Anconeus Biceps brachii x x Extensor metacarpi radialis	Propatagialis brevis Anconeus Biceps brachii Humerotriceps x Extensor metacarpi radialis	
Supination	Pronator Flexor carpi ulnaris Entepicondyloulnaris Scapulotriceps x Humerotriceps	Pronator Flexor carpi ulnaris Entepicondyloulnaris Scapulotriceps x Humerotriceps	Pronator Flexor carpi ulnaris Entepicondyloulnaris Scapulotriceps Biceps brachii Humerotriceps	Pronator Humerotriceps
**(C) WRIST MUSCLES**				
Flexion	**Flexor carpi ulnaris** x Ulnimetacarpalis ventralis	**Flexor carpi ulnaris** Flexor digitorum **Ulnimetacarpalis ventralis**	**Flexor carpi ulnaris** Flexor digitorum Ulnimetacarpalis ventralis	Ulnimetacarpalis ventralis
Extension	**Extensor metacarpi radialis** Extensor pollicis longus Extensor carpi ulnaris Extensor indices longus Extensor digitorum communis x Flexor digitorum Ulnimetacarpalis ventralis	**Extensor metacarpi radialis** Extensor pollicis longus Extensor carpi ulnaris Extensor indices longus Extensor digitorum communis Flexor carpi ulnaris Flexor digitorum Ulnimetacarpalis ventralis	**Extensor metacarpi radialis** Extensor pollicis longus Extensor carpi ulnaris Extensor indices longus Extensor digitorum communis x Flexor digitorum Ulnimetacarpalis ventralis	
Adduction	**Flexor digitorum** **Flexor carpi ulnaris** Extensor metacarpi radialis Ulnimetacarpalis ventralis Extensor pollicis longus	Flexor digitorum Flexor carpi ulnaris Extensor metacarpi radialis Ulnimetacarpalis ventralis Extensor pollicis longus	**Flexor digitorum** Flexor carpi ulnaris Extensor metacarpi radialis Ulnimetacarpalis ventralis Extensor pollicis longus	
Abduction	Extensor carpi ulnaris Extensor digitorum communis Extensor metacarpi radialis Extensor indices longus Flexor carpi ulnaris Extensor pollicis longus Flexor digitorum Ulnimetacarpalis ventralis	**Extensor carpi ulnaris** Extensor digitorum communis x Extensor indices longus **Flexor carpi ulnaris** x x Ulnimetacarpalis ventralis	Extensor carpi ulnaris Extensor digitorum communis Extensor metacarpi radialis Extensor indices longus Flexor carpi ulnaris Extensor pollicis longus x Ulnimetacarpalis ventralis	Extensor metacarpi radialis
Pronation	Flexor carpi ulnaris Flexor digitorum Extensor carpi ulnaris Ulnimetacarpalis ventralis Extensor metacarpi radialis x Extensor digitorum communis	**Flexor carpi ulnaris** Flexor digitorum Extensor carpi ulnaris Ulnimetacarpalis ventralis Extensor metacarpi radialis Extensor pollicis longus Extensor digitorum communis	**Flexor carpi ulnaris** **Flexor digitorum** Extensor carpi ulnaris Ulnimetacarpalis ventralis Extensor metacarpi radialis x x	
Supination	**Extensor metacarpi radialis** Extensor pollicis longus Extensor indices longus Flexor carpi ulnaris Ulnimetacarpalis ventralis Extensor digitorum communis Flexor digitorum	**Extensor metacarpi radialis** Extensor pollicis longus Extensor indices longus x x Extensor digitorum communis x	**Extensor metacarpi radialis** Extensor pollicis longus Extensor indices longus Flexor carpi ulnaris Ulnimetacarpalis ventralis Extensor digitorum communis x	Flexor carpi ulnaris Ulnimetacarpalis ventralis Flexor digitorum

*Potential muscle contributions, determined as a percentage of the total moment at a joint, averaged over the entire stroke cycle. Color codes: bold black, muscle moment ≥ 50% total moment; black, 50% > moment ≥ 20%; purple, 20% > moment ≥ 5%; red, 5% > moment. “Pronator” includes P. profundus and P. sublimis; “Flexor digitorum” includes F. d. sublimis and F. d. profundus. “Ontogenetic differences” column refers to muscles in which moments differ across age classes*.

#### Muscle function

Whereas moment arms represent the potential for a muscle to perform an action, inverse dynamics and static optimization analyses provide timing and intensity of muscle activity and thus suggest specific functional roles for each muscle during wing flapping. Here, we defined the upstroke-downstroke transition as the point at which the tip of the manus began moving downward (i.e., closer to the sternum in a transverse plane), and the downstroke-upstroke transition as the point at which the tip of the manus began moving upward. However, both transitions were initiated at the shoulder joint, and progressed proximal to distal (shoulder 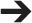 elbow 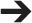 wrist). Thus, at the start of downstroke (as defined by position of the manus), the humerus was already being depressed and pronated, and just beginning to retract. Similarly, at the start of upstroke, the humerus was already being elevated, whereas the tip of the manus was just beginning to reverse direction.

Overall, muscle actions determined from muscle moment arms, simulated muscle activity, and flapping kinematics were consistent with empirical studies of live birds. As in previous work (Dial et al., 1991; Dial, 1992a; Poore et al., 1997a; Biewener, 2011; Robertson and Biewener, 2012), the pectoralis and supracoracoideus emerged in our simulations as the main drivers of the downstroke and upstroke, respectively. The pectoralis was active during the upstroke-to-downstroke transition, acting to decelerate and then depress, retract, and pronate the humerus (Table S8). The supracoracoideus acted in an opposite pattern, contracting during the downstroke-upstroke transition to decelerate and then elevate and supinate the humerus. Both muscles were aided by the activity of smaller muscles spanning the shoulder joint. The pectoralis was supplemented by the coracobrachialis posterior and subcoracoideus (mainly retraction), whereas the supracoracoideus was supplemented slightly by the deltoideus major (elevation, protraction). The scapulohumeralis caudalis (elevation, retraction or protraction, pronation, stabilization), latissimus dorsi (retraction, elevation, stabilization), and scapulotriceps (retraction, elevation, stabilization) assisted both the pectoralis and supracoracoideus. Most of these simulated functions were consistent with previous work (Dial et al., 1991; Dial, 1992a; Poore et al., 1997a; Biewener, 2011; Robertson and Biewener, 2012), though some differences were evident (Table S8).

Whereas the pectoralis and supracoracoideus dominated movement at the shoulder joint, our simulated muscle functions were more evenly distributed at the elbow and wrist, and muscles were not distinguished by clear downstroke or upstroke activity. Between early-to-mid upstroke and early-to-mid downstroke, several muscles were activated to unfurl the wing by extending the elbow (scapulotriceps, humerotriceps, flexor carpi ulnaris, pronator profundus) and then the wrist (extensor metacarpi radialis, flexor digitorum sublimis, extensor carpi ulnaris). Simultaneously, the wrist was abducted (extensor carpi ulnaris, extensor metacarpi radialis), which would allow the primary feathers to be untucked from their folded position against the body (Heers et al., 2016). Elbow abduction (anconeus, extensor metacarpi radialis, extensor carpi ulnaris, supinator, extensor digitorum communis) occurred as well but preceded wrist abduction, initiating the downstroke-to-upstroke transition.

Beginning in early-to-mid downstroke, in preparation for tucking in the wing during the downstroke-upstroke transition, the elbow (pronator sublimis, extensor metacarpi radialis; small contributions from brachialis, supinator, anconeus, extensor digitorum communis, extensor carpi ulnaris) and then wrist (flexor carpi ulnaris, flexor digitorum profundus) began to flex, and the wrist began to adduct (flexor digitorum, flexor carpi ulnaris). Elbow adduction (pronator, humerotriceps and scapulotriceps, flexor carpi ulnaris, biceps brachii; small contributions from entepicondyloulnaris) also occurred but preceded wrist adduction, beginning in late upstroke and continuing into mid-downstroke.

Long axis rotation at the elbow and wrist was more complex, generally acting in opposition (see note on washout in Heers et al., 2016) and reversing directions several times, which would allow the bird to fine-tune the angle of attack along the wing (Biewener, 2011). Elbow pronation was likely achieved by the tensor propatagialis brevis and biceps brachii, and elbow supination by the flexor carpi ulnaris, scapulotriceps, and humerotriceps. Wrist pronation seemed to be driven by the flexor carpi ulnaris and flexor digitorum, and wrist supination by the extensor metacarpi radialis and extensor digitorum communis.

As with the shoulder muscles, the simulated functions of muscles spanning the elbow and/or wrist were consistent with data on live birds (Dial et al., 1991; Dial, 1992a; Poore et al., 1997a; Biewener, 2011; Robertson and Biewener, 2012), with one informative exception (Table S8). Our models suggested that the pronator muscles did not actually contribute to pronation at the elbow: although the pronator profundus and pronator sublimis were activated while the elbow was pronating, they had a supinating moment and thus helped to stabilize against excessive pronation.

Finally, as in live birds, all of our modeled muscles had a decelerating and/or stabilizing function at some point during the stroke cycle. For example, the pectoralis and supracoracoideus were initially activated when their moment arms opposed the current shoulder rotations, and thus decelerated the humerus at the end of the upstroke or downstroke, respectively. The antagonistic triceps and biceps muscles were sometimes co-activated, and the flexor digitorum stabilized against abduction whereas the extensor digitorum communis and extensor carpi ulnaris stabilized against flexion and adduction at the wrist (Table S8).

### Development of the avian flight apparatus

#### Joint moments

**Figure 7:**
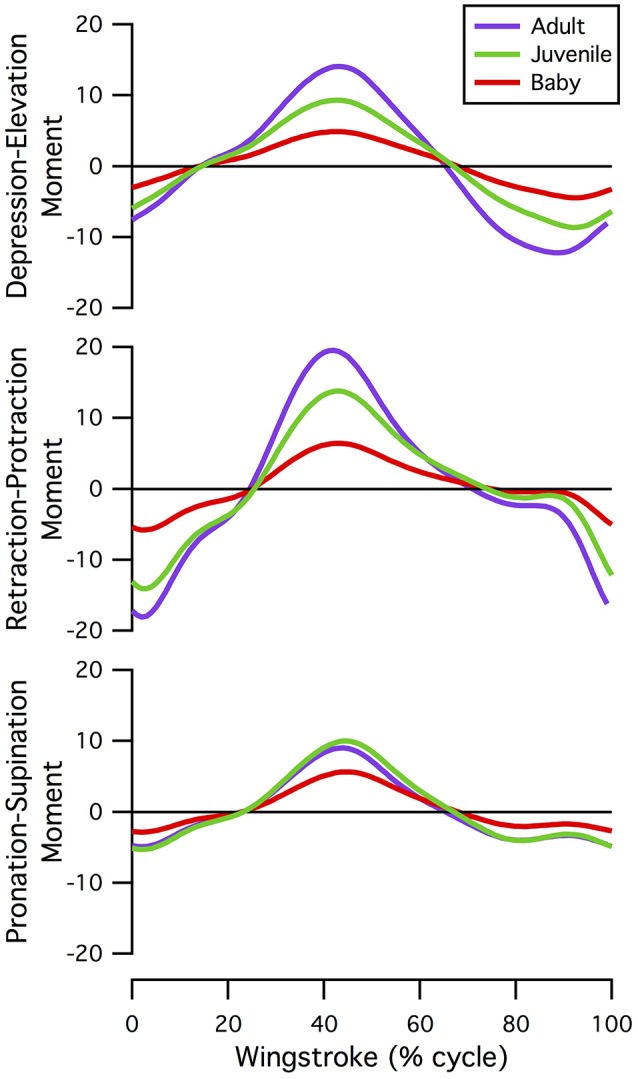
Normalized shoulder moments. Moments at the shoulder joint, standardized by body weight and moment arm lengths of the pectoralis and supracoracoideus (averaged over one wingbeat); all models were simulated under identical conditions (adult kinematics and no aerodynamic force). Joint moments increase from baby to juvenile to adult, even when accounting for body size.

Maximum joint moments associated with flap-running increased from baby to juvenile to adult, even when accounting for body size (Figure 7; Table S4). Long-axis rotation moments were small compared to other motions. At the elbow and wrist, abduction-adduction moments were generally greater than those for flexion-extension, reflecting the high inertial torques associating with flapping and aerodynamic force production. Similarly, at the shoulder, elevation-depression moments were greatest in the adult and juvenile, but protraction-retraction was greatest in the baby; young chicks tend to flap in a more craniocaudal direction than older birds (Heers et al., 2011).

#### Ontogenetic limits on locomotor capacity: feathers vs. muscles

We initially hypothesized that musculoskeletal and feather morphology would equally limit locomotor capacity, such that baby and juvenile chukars would have high muscle activations (H_0_). However, in general, the baby and juvenile models had low simulated muscle activations, particularly at the shoulder (generally <0.5) (Figures 4, 6). Activations were higher at the elbow and wrist, but only slightly higher than activations in the adult (Table S7). These relatively low activations—particularly in the power-generating shoulder muscles—suggest that flapping performance in developing chukars is more limited by other, non-muscular factors (e.g., feathers or skeletal kinematics).

**Figure 8:**
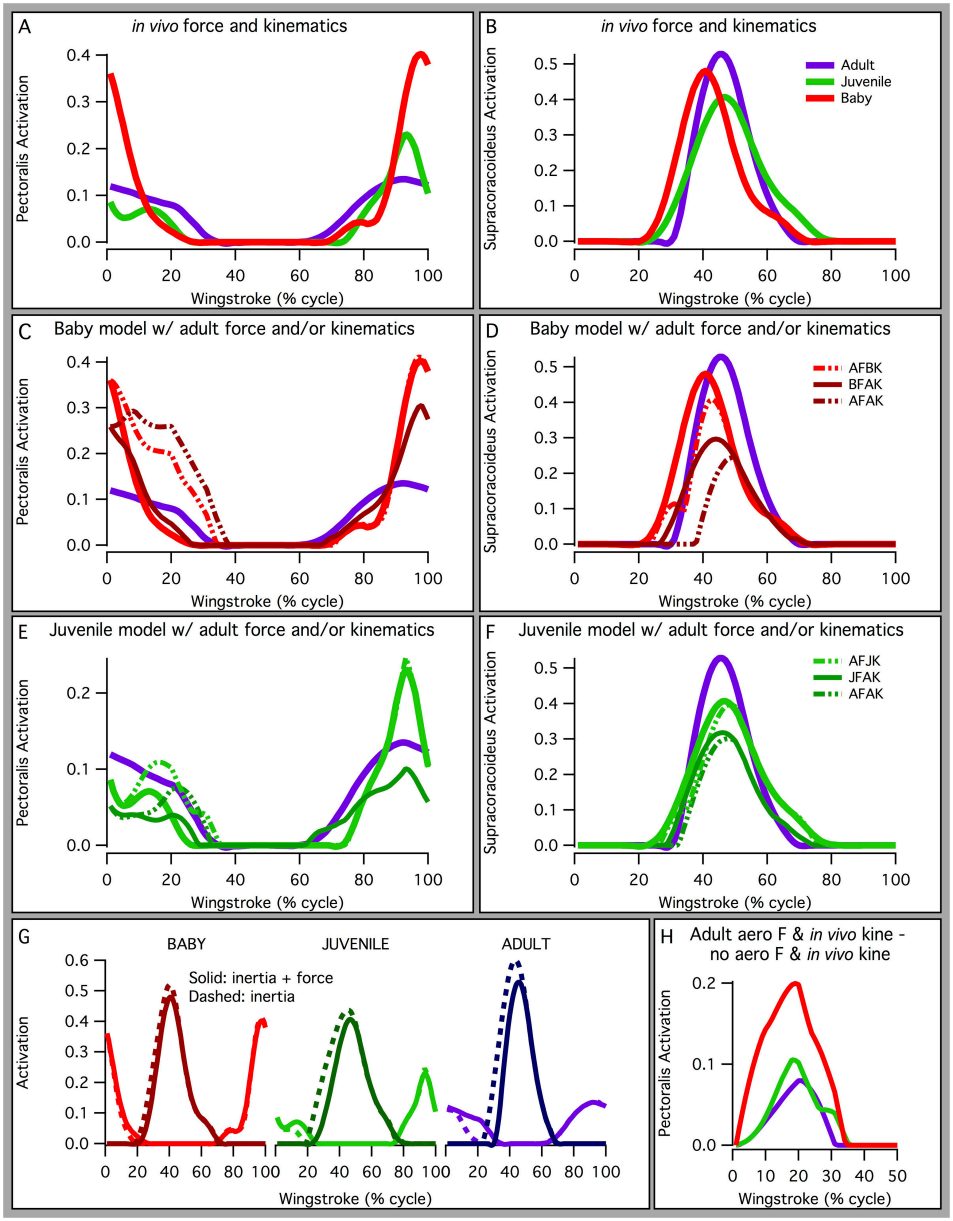
Manipulations in aerodynamic force and kinematics: effects on pectoralis **(A,C,E,G,H)** and supracoracoideus **(B,D,F,G)** activation levels. Overall, increasing aerodynamic force production to adult levels (in terms of percent body weight) in the baby and juvenile models increases activation of the pectoralis muscle during mid-downstroke, but not to high levels (<0.4, baby; <0.15, juvenile). Supracoracoideus activation decreases, because increased aerodynamic force production helps decelerate the wing and prepare for upstroke. **(A,B)** *In vivo* kinematics and aerodynamic force production; **(C–F)** baby or juvenile model simulated with different combinations of aerodynamic force (BF, baby force; JF, juvenile force; AF, adult force) and kinematics (BK, baby kinematics; JK, juvenile kinematics; AK, adult kinematics), *in vivo* activations for adult and baby or juvenile still shown; **(G)** *in vivo* kinematics with (solid lines) or without (dashed lines) aerodynamic force production, pectoralis indicated by lighter colors (red, light green, purple), and supracoracoideus by darker colors (maroon, dark green, indigo); **(H)** activation due to aerodynamic force production: *in vivo* kinematics with no aerodynamic force production subtracted from *in vivo* kinematics with adult aerodynamic force production, to account for ontogenetic differences in inertial properties.

Kinematic and morphological manipulations (Table 1) revealed several trends. First, for all age classes, improving feather and wing quality (represented by increased aerodynamic forces) increased activation of the pectoralis muscle during the downstroke (Figures 8C,E,G) but decreased activation of the supracoracoideus muscle during the downstroke-upstroke transition (Figures 8D,F,G). Though somewhat counterintuitive, aerodynamic force helped to slow the wing in late downstroke and thus reduced the role of the supracoracoideus muscle in deceleration (reduced activation by 13% in adult model).

Second, for the baby and juvenile models, simulating WAIR with adult kinematics reduced peak activation of both the pectoralis (Figures 8C,E) and supracoracoideus (Figures 8D,F), presumably because adult chukars use a lower angular velocity during WAIR [61 vs. 66–69 rad s^−1^ (Heers et al., 2011)].

Finally, manipulations indicated that the muscles of baby and particularly juvenile chukar models were capable of flapping more aerodynamically effective wings. Under all conditions (Table 1), the juvenile model's muscles were able to flap a better, more adult-like wing: increasing aerodynamic force to adult magnitudes increased activation of the pectoralis muscle, but not much above adult levels of activation (Figure 8E; reserve actuators unchanged). For the baby model, increasing aerodynamic force to adult magnitudes increased activation of the pectoralis muscle much more substantially, above adult levels but less than maximal [0.023 during mid-downstroke (*in vivo*) vs. 0.20 (Treatment 1—baby kinematics) or 0.26 (Treatment 2—adult kinematics)] (Figure 8C; reserve actuators changed by <3%). This suggests that baby and particularly juvenile chukars are capable of flapping more aerodynamically effective wings.

Inertial properties contributed to, but did not alter, these trends. During early-downstroke, when aerodynamic force production was rising, inertial properties accounted for 71–78% of pectoralis activation in all three models (Figure 8G, dashed vs. solid lines). During wing turnaround (downstroke-upstroke and upstroke-downstroke transitions), pectoralis and supracoracoideus activations could be attributed almost entirely to overcoming limb inertia because aerodynamic force was not being produced. However, as mentioned earlier, aerodynamic force production reduced supracoracoideus activation by helping to decelerate the wing in late downstroke. Wing inertia therefore played a substantial role in determining muscle activation throughout the stroke cycle. This was particularly true for the adult model, which experienced greater inertial forces even when standardizing for body size (Figure 7).

When inertial effects were eliminated (Figure 8H), pectoralis activation in the juvenile model was still low—only slightly above adult levels—and pectoralis activation in the baby model was still 2–3 times higher than the adult. Our simulations thus collectively suggest that the relatively low activations of baby, and particularly juvenile, flight muscles occurred partially because developing chukars are small (low wing inertia, Figure 7), and partially because in developing chukars feather quality (capacity for aerodynamic force production) limits flapping performance more than muscle morphology—i.e., baby and especially juvenile chukars appear to be capable of flapping better, more adult-like wings (Figures 8C–F).

#### Compensatory mechanisms

Given our initial prediction (H_0_), the underutilized potential of the baby and especially juvenile pectoral muscles is somewhat surprising. However, our models and simulations suggest that chukars acquire a number of compensatory mechanisms by the time they become flight capable, such that 18–20 day old juvenile birds are not hampered by muscle morphology.

##### Muscle compensation

Proportionally, developing chukars have much smaller muscles than their adult counterparts, especially for muscles acting around the shoulder (Figure S6). However, all else being equal, smaller animals tend to be relatively stronger than larger animals because the ratio of muscle area (proportional to force) to body weight declines with size, as does moment arm length relative to the inertial moments that must be opposed.

**Figure 9:**
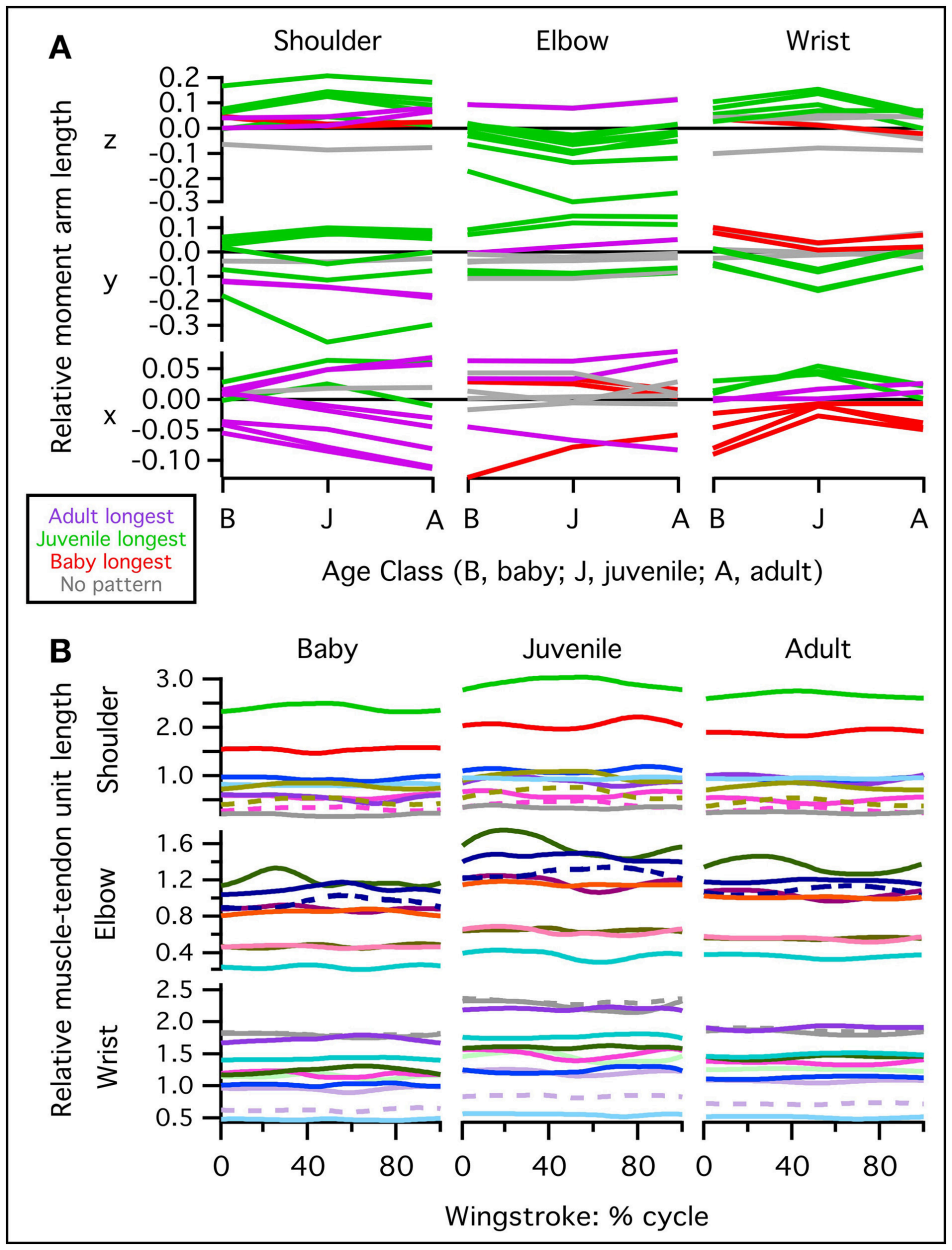
Normalized muscle moment arms and lengths. The juvenile model tends to have relatively long moment arms and muscle lengths compared to the adult and baby models. **(A)** Moment arms for muscles crossing the shoulder, elbow, or wrist, averaged over the stroke cycle and standardized by notarium length. Different lines are for different muscles. Red lines: moment arm is greatest (most positive or most negative) in the baby model; green: moment arm is greatest in the juvenile model; purple: moment arm is greatest in the adult model; gray: no ontogenetic trend. The juvenile has proportionally long z (elevation-depression or extension-flexion) and y (protraction-retraction or abduction-adduction) moment arms, and the adult has long x (supination-pronation) moment arms at the shoulder. **(B)** Muscle-tendon unit (MTU) length through one wingbeat cycle (0–100%), standardized by notarium length; different colors represent different muscles crossing the shoulder, elbow, or wrist.

**Figure 10:**
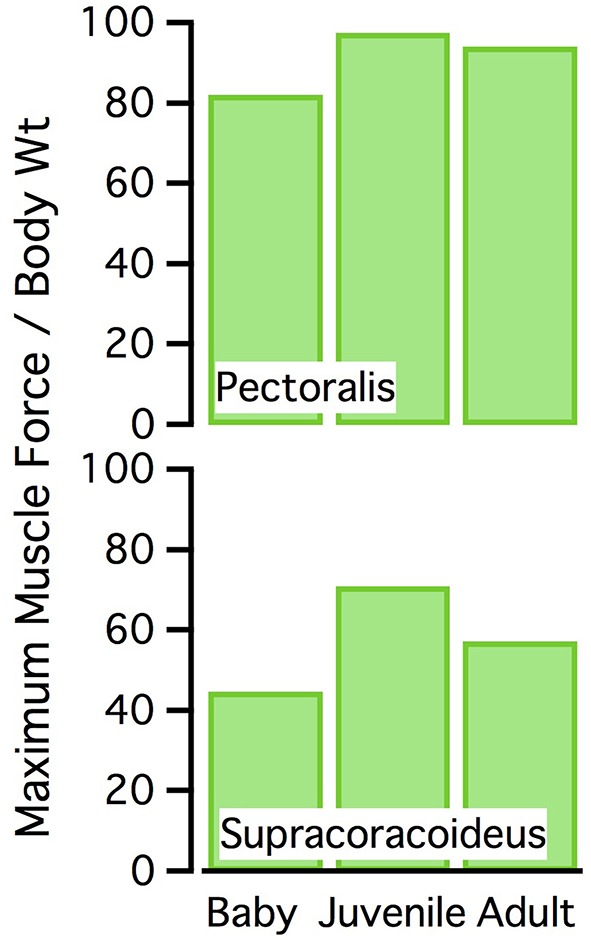
Normalized muscle force. Maximum isometric muscle force as a percentage of body weight, for the pectoralis and supracoracoideus (both sides of the body). The juvenile model has the highest maximum force, for its body size.

**Figure 11:**
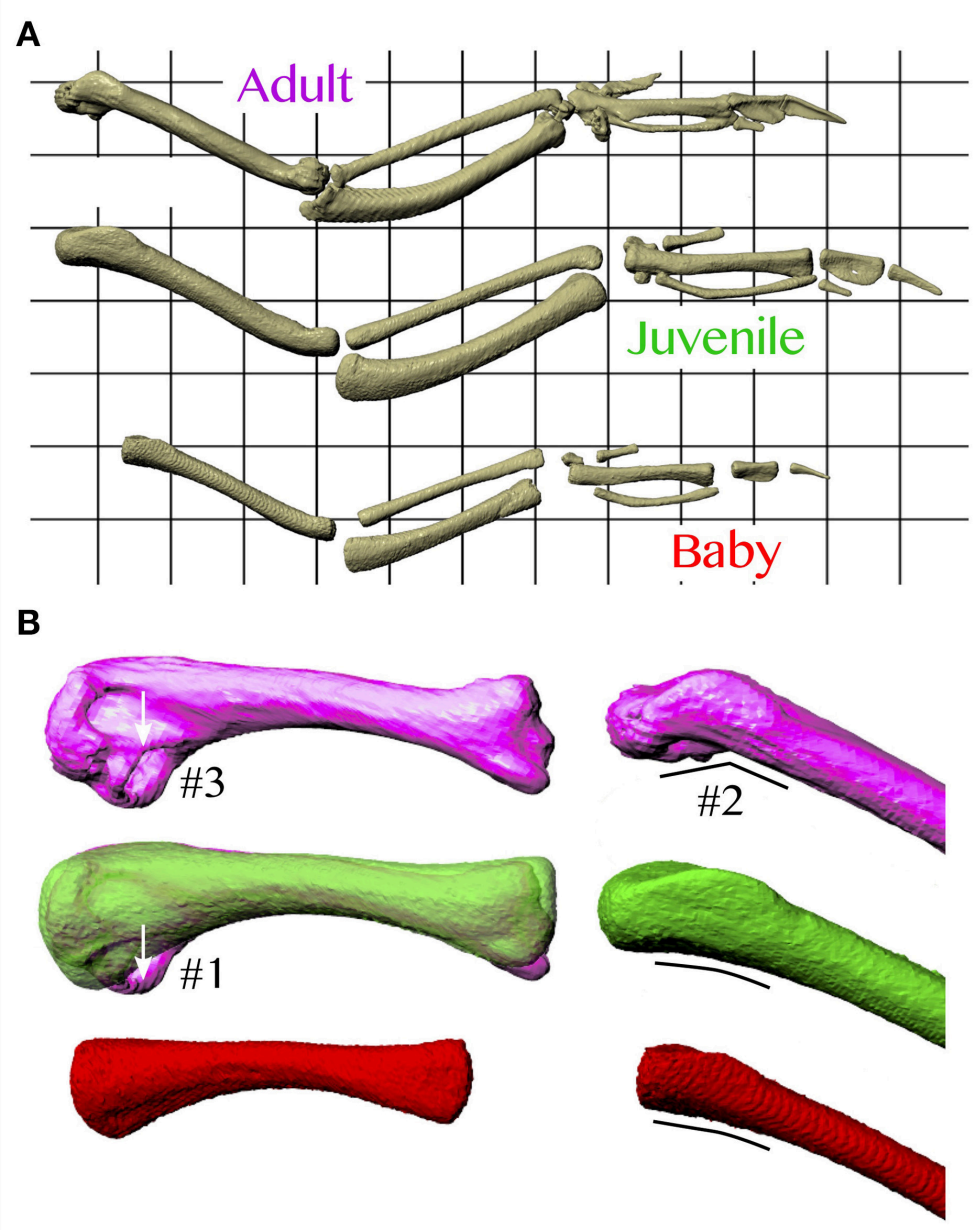
Skeletal anatomy. **(A)** Images of right forelimbs in dorsal view, standardized by notarium length. The proportionally long moment arms and muscle-tendon lengths (Figure 9) of the juvenile are likely due, at least partially, to its proportionally long and sometimes wide limb bones. **(B)** Images of right humeri (adult, purple; juvenile, green; baby, red), standardized by notarium length; left images in posterior view, right images in dorsal view. The proportionally long moment arms of the adult for long axis rotation at the shoulder (Figure 9) are at least partially due to its expanded bicipital crest (#1), exaggerated angle between the head and shaft of the humerus (#2), and expanded margo caudalis (#3).

In addition, based on our models, juvenile chukars seemed to compensate for small flight muscles by having relatively long moment arms and muscles (Figure 9) compared to baby and adult chukars, as well as high muscle cross-sectional areas for their body weight (Figure 10). The relatively long moment arms of the juvenile were mainly in the z (elevation-depression or extension-flexion) and y (protraction-retraction or abduction-adduction) directions [see (Heers et al., 2016) and references therein for further explanation of coordinate systems]. These moment arm increases were at least partially attributable to the juvenile's proportionally long and/or wide limb bones (Figure 11A), which allowed for long muscle fibers and shifted muscle lines of action away from the wing joints. In contrast, our models suggested that adult chukars have proportionally long moment arms at the shoulder in the x direction (supination-pronation). This appears to be the result of an expanded bicipital crest, exaggerated angle between the head and shaft of the humerus, and expanded margo caudalis (Figure 11B). All of these features are absent in developing chukars but allow for long moment arms perpendicular to the long axis of the limb in adult birds.

**Figure 12:**
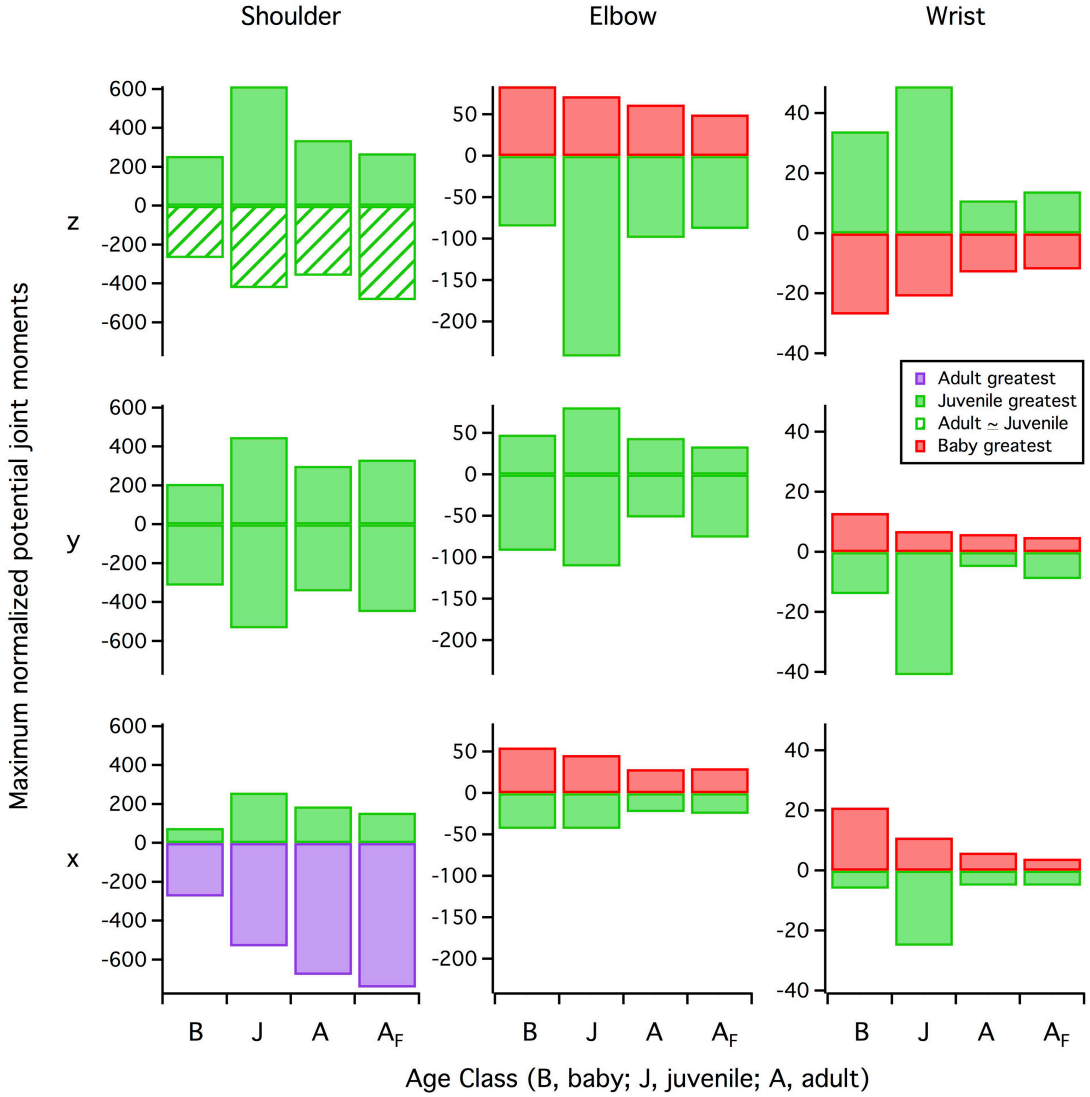
Maximum potential joint moments. Maximum potential joint moments (positive or negative muscle moment arms (in z, y, and x directions) multiplied by maximum isometric muscle force, summed for all muscles at each 1% of the stroke cycle, then averaged over the stroke cycle and normalized by notarium length and body weight) are greatest in the juvenile model for more than half of the possible joint motions. Red bars, baby has the greatest (most positive or most negative) joint moment; green bars, juvenile has the greatest joint moment; green hashed bars, juvenile and adult have similar joint moments; purple bars, adult has the greatest joint moment. Row z, elevation (+ moments) vs. depression (−) (shoulder joint) or extension (+) vs. flexion (−) (elbow, wrist); row y, protraction (+) vs. retraction (−) (shoulder) or abduction (+) vs. adduction (−) (elbow, wrist); row x, supination (+) vs. pronation (−) (all joints). B, baby model (7–8 days); J, juvenile model (18–20 days); A, adult model (>100 days) during wing-assisted incline running; A_F_, adult model during ascending flight, for comparison.

Given that a muscle's ability to induce joint movement depends on both its moment arm and its capacity to produce force (proportional to physiological cross-sectional area), the proportionally long z and y moment arms and proportionally high cross-sectional areas of our juvenile model yielded high potential joint moments for elevation-depression and protraction-retraction (Figure 12). These high potential joint moments suggest that the juvenile model was not limited by the amount of force the muscles could produce in elevation-depression and protraction-retraction. Plotting muscle force against muscle activation under a standardized set of conditions (adult kinematics, no aerodynamic force) supported this inference: for a given level of muscle activation, force production in the juvenile model was relatively similar to that of the adult (sometimes higher, sometimes lower; Figure S7), indicating that muscles in the juvenile model, though small, were effective for the model's (small) body size.

##### Feather compensation

Developing chukars have less aerodynamically effective feathers than adults: compared to adults, younger birds produce less aerodynamic force for their body weight (Figure 3), and less lift per unit drag (Heers et al., 2011). This is partially due to small wing size (Dial et al., 2006; Heers and Dial, 2012), and partially due to feather microstructure (Heers et al., 2011). Previous work has shown that during WAIR, developing chukars compensate some for their high-drag wings by flapping with a steep stroke plane angle, such that drag mainly supports body weight (Heers et al., 2011). 18–20 day old juvenile chukars additionally appeared to compensate for poorer quality feathers by having relatively long feathers (Figure S8).

**Figure 13:**
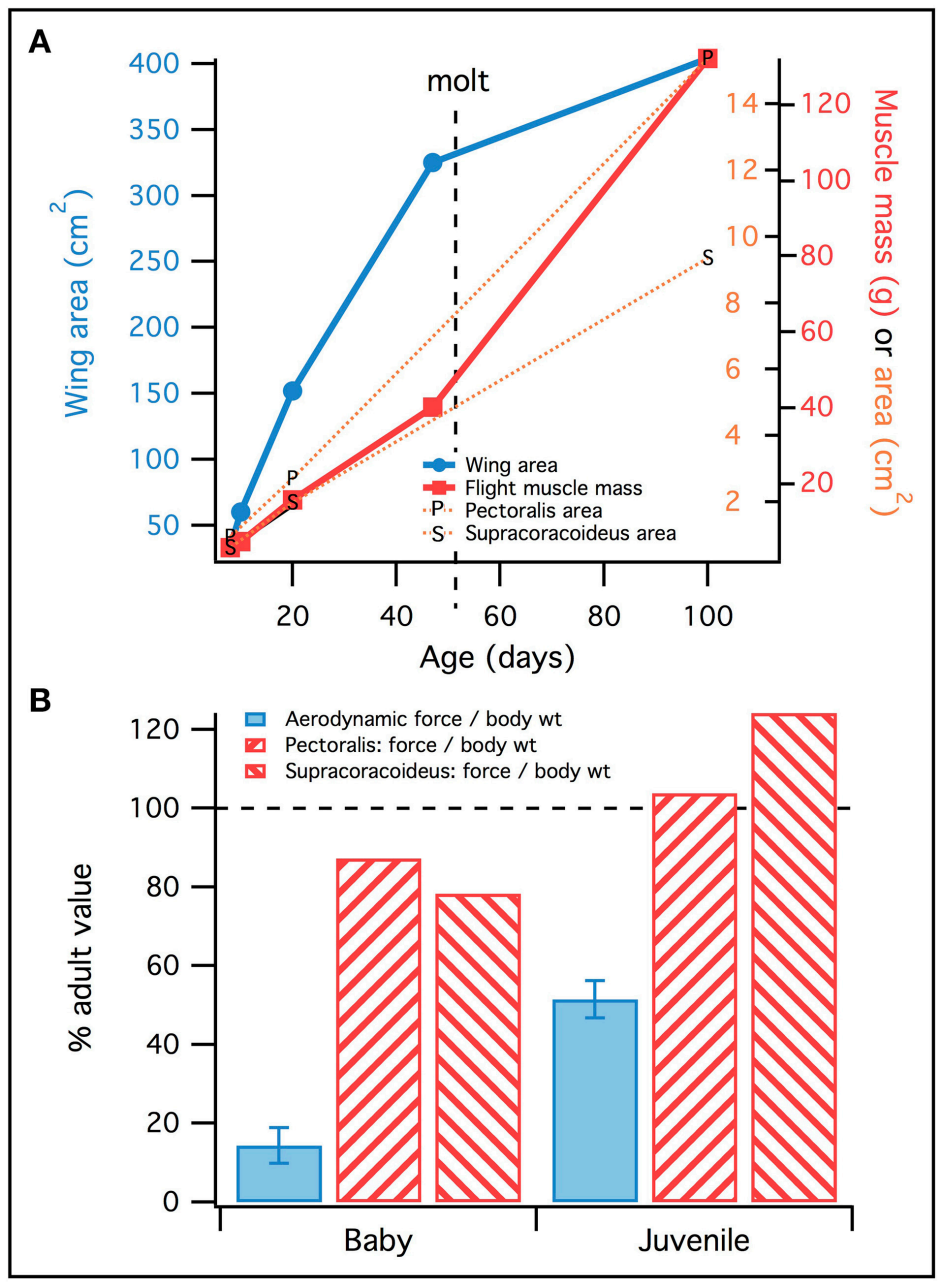
Feathers vs. muscles**. (A)** Wing area (blue) increases faster than muscle mass (red; all flight muscles) or physiological cross-sectional area (orange), until molt. This suggests that early in ontogeny, muscles must be “pre-equipped” to flap bigger and better wings. **(B)** Consistent with (A), muscles reach adult levels of performance (maximum force/body weight; red) more rapidly than wings (force/body weight, from Heers et al., 2011; blue), suggesting that muscles are more functionally developed than feathers in baby and juvenile chukars. Data in **(A)** represents two wings, and muscles on both sides of the body.

However, feather compensation seemed to be less substantial than muscle compensation. Our results suggested that long muscles and long muscle moment arms, coupled with small body size, allowed the 18–20 day old chukar model to produce high muscle forces per unit body weight—greater than those produced by adults (Figure 10). Simulations also showed that juvenile and adult chukars activated their muscles to similar levels (Figure 8; Table S7). Together, these results implied that juvenile chukars are not hampered by muscle morphology. In contrast, despite having proportionally long feathers, 18–20 day old chukars produced less aerodynamic force per unit body weight than adults (Figure 13). Feather morphology therefore seemed to limit flapping performance more than muscle morphology in developing chukars: accounting for body size, our models suggested that the feathers of immature chukars produced proportionally less force than their muscles, and that baby and especially juvenile chukars would be capable of flapping better, more adult-like wings. Thus, H_1_ is supported over H_0_.

## Discussion

### Comparison with live birds

Overall, our musculoskeletal models and simulations appear to be reasonable, at least qualitative approximations of the chukar flight apparatus, because muscle activity and functional roles are largely consistent with data from live birds.

#### Muscle activity

In general, the timing of simulated muscle activations during WAIR was very similar to the timing of muscle activity in live birds during similar behaviors [ascending flight—similar body orientation and flapping kinematics (Baier et al., 2013)] (Figure 4). This was particularly true for the two most important flight muscles, the pectoralis (downstroke) and supracoracoideus (upstroke), which also shortened, lengthened, and developed force similarly to flying and flap-running pigeons (Figure 5). For the seven muscles showing differences between simulated and *in vivo* muscle activity, discrepancies could be real—due to differences in kinematics and/or muscle morphology (WAIR vs. flight, chukars vs. pigeons)—or may reflect model simplification. For example, muscle origins and insertions were modeled as points, but many muscles have broad origins (e.g., scapulohumeralis caudalis originates from the entire lateral surface of the scapula but was modeled as a “point” origin midway along the scapula). Limitations of static optimization might also have contributed to differences between modeled vs. *in vivo* activations (e.g., optimizing to minimize squared muscle activations; assuming that prior/future events in a cycle do not influence others; tendon elasticity ignored). Future analyses can discriminate between some of these possibilities, but in general, our simulated muscle activations during WAIR were similar to patterns of muscle activity in live birds. This similarity is interesting because the simulations involved rapid, intense behaviors that would be expected to be very non-static and hence potentially result in large discrepancies between experimental (i.e., dynamic) and simulation (i.e., static) results. Forward dynamic analysis is beyond the scope of this study but will be done in follow-up analyses.

#### Muscle function

Muscle functions based on simulated muscle activity, moment arms, and kinematics were also largely consistent with previously suggested functions (Table S8) (Dial et al., 1991; Dial, 1992a; Poore et al., 1997b; Biewener, 2011; Robertson and Biewener, 2012). Discrepancies between *in vivo* and simulated functions occurred in the coracobrachialis posterior, subscapularis, deltoideus major, and scapulohumeralis caudalis muscles, and appear to have resulted from kinematic differences between WAIR and flight. During WAIR, the humerus is kept relatively elevated and retracted compared to level flight, which alters the moment arms of these shoulder muscles (Table S8). Differences also occurred in the pronator sublimis and pronator profundus, but our simulations suggest that in birds these muscles actually oppose rather than cause pronation, due to the unique orientation of the avian ulna and radius with respect to the humerus (articulation with humerus rotated compared to human condition; Table S8). Although the pronator muscle pronates the forearm in humans, “pronator” is probably a misnomer in birds. In short, functional discrepancies between *in vivo* and our simulated data occurred, but likely reflect kinematic differences between WAIR and flight or nomenclatural inaccuracies.

#### Magnitude of muscle activations

The magnitudes of simulated muscle activations during WAIR were generally low for all three age classes, particularly at the shoulder. This is consistent with previous work demonstrating that WAIR is an “easy” flapping behavior compared to flight, at least for adult birds (Jackson et al., 2011b). However, even in the adult model the simulated activations of some muscles, particularly the supracoracoideus (~0.5; Figure 4), might be somewhat higher than expected. This is probably due to simulation limitations, namely the inability of static optimization to account for dynamic effects.

Whereas most flight muscles are parallel-fibered with short tendons, the supracoracoideus muscle is pennate with a long tendon that likely stores a substantial amount of elastic energy, possibly contributing 28–60% of the net work done by the supracoracoideus (Tobalske and Biewener, 2008). Static optimization does not allow for elastic energy storage and release (Delp et al., 2007; Rankin et al., 2016) and thus might result in erroneously high activations in muscles like the supracoracoideus, and/or greater contributions from the reserve actuators used in our study (Figure S4). It is also possible that the fast twitch (white/fast glycolytic) muscle fibers characteristic of chukars and other galliform birds make their muscles more effective than the generic muscles modeled (e.g., in terms of maximal force per unit area, or dynamic effects not simulated in our static analyses—such as maximal muscle contraction velocity). Force enhancement following muscle stretching has been demonstrated for some vertebrates (Herzog and Leonard, 2002) and may play a role avian flight, given that the pectoralis and supracoracoideus are stretched substantially prior to the downstroke and upstroke, respectively, but this has not been explored experimentally. Finally, ligaments or bony restrictions at joints, and dynamic events such as wing clapping at the end of upstroke, could reduce active muscle contributions by passively restricting the range of motion at joints. Future analyses will assess these possibilities.

### Feather vs. muscle development

Compared to adults, developing chukars have small wings and/or less aerodynamically effective feathers (Dial et al., 2006; Heers et al., 2011), proportionally small muscles (Heers and Dial, 2015), and less specialized skeletons with smaller bony projections for muscle attachment (Heers and Dial, 2012; Heers et al., 2016). Immature chukars appear to compensate partially for their underdeveloped flight apparatuses in several ways. Our models showed that because developing chukars are small, they have low wing inertia (Figure 7). 18–20 day old juvenile chukars additionally appear to compensate by having relatively long wing feathers (Figure S8), very low wing loading (Jackson et al., 2009), and proportionally long muscles with long moment arms that contribute to high potential joint moments (Figure 12). However, feather compensation appears to be less substantial than muscle compensation (Figure 13), and baby and especially juvenile chukars seem to be capable of flapping better, more adult-like wings (Figure 8). Thus, locomotor performance in developing chukars may be limited more by wing morphology and aerodynamic force production than by muscle morphology.

It is possible that the muscles of young chukars differ histologically from the muscles of adults, just as feather microstructure differs between immature and adult chukars (Heers et al., 2011). If so, the muscles of developing chukars may not be as effective as our simulations would suggest. However, previous studies indicate that our 18–20 day juvenile model's muscle physiology likely is accurate. For example, Jones (1982) observed no difference in structural detail or fiber size in the pectoralis between fledged and adult House Sparrows (*Passer domesticus*). The muscle fibers of sparrows about to fledge (15 days) were adult-like in composition and organization, though smaller in cross-sectional area (consistent with smaller muscle mass). Similarly, Ricklefs (1979) found that the pectoralis of juvenile Japanese Quail (*Coturnix coturnix*) had an adult-like water index (indicative of muscle functional maturity) by the time the birds began to fly (87 or 100% adult value at 20 and 30 days post-hatch, respectively; *C. coturnix* reported to fly at 30 days but 20 days is probably more similar to our 20 day old chukars). Finally, Tobalske et al. (2017) tracked pectoralis function during chukar ontogeny and found that activation and contractile behavior differed in very young chukars (≤9 days; long EMG duration and lower EMG amplitude, strain, fractional shortening, and contractile velocity) but converged on adult levels between 9 and 20 days post-hatch.

Collectively, these studies indicate that our 7–8 day baby model likely overestimates muscle capacity (e.g., maximal force), whereas our 18–20 day juvenile model is probably accurate in this regard. Maximum isometric muscle force of the pectoralis muscle would have to decrease by 83% (functional maturity 17% of adult) to prevent our juvenile model from flapping at adult levels of aerodynamic force production. Even newly hatched quails have water indices that are 50% of the adult value (Ricklefs, 1979), indicating that juvenile chukars should be able to flap adult-like wings even if their muscles are not quite as mature as our models assumed.

There are at least two reasons why feathers might be expected to be more limiting than muscles early in chukar ontogeny. First, wing size increases more rapidly than muscle size in developing chukars (Figure 13), so muscles must be “pre-equipped” to flap better wings. For example, between 8 and 10 days post-hatching, wing area increased by 82% but muscle cross-sectional area increased by <30%. Wing size increases more rapidly than muscle size until ~50 days post-hatch. At this stage juveniles molt and replace their poorer quality juvenile plumage with high quality adult feathers (Heers et al., 2011), and muscle size begins to increase more rapidly.

Second, unlike adult birds during molt, developing birds must grow all of their feathers simultaneously, in addition to growing other body parts. A number of studies [reviewed in (Butler et al., 2008)] suggest that developing birds probably do not have enough resources to grow high quality feathers. Whereas bones and muscles can be continuously modified for improvements in performance, feathers are not modified after emerging from the sheath—feather quality does not improve until the juvenile plumage is molted and replaced by adult feathers (Heers et al., 2011). Feather development thus may be a case of “something is better than nothing”: it is likely better to grow a poorer quality wing quickly than a higher quality wing slowly, and to compensate by growing longer feathers at 18–20 days and then molting feathers later on. Muscles and bones are not constrained by this style of growth.

It is possible that if chukars activated their muscles more they could increase wingbeat frequency and thereby increase aerodynamic force production (∞ velocity^2^), such that feathers would be less limiting than they appear to be. However, this does not seem to occur. Although 7–8 day old chukars do use higher wingbeat frequencies during controlled aerial descents (Jackson et al., 2009), during WAIR they do not seem to increase wingbeat frequency to increase aerodynamic output and thereby ascend steeper inclines. This could reflect a neurological constraint—chukars adopt adult-level wingbeat frequencies early in ontogeny (Jackson et al., 2009). Alternatively, perhaps at higher wingbeat frequencies the more compliant feathers and/or wing joints of young birds would excessively deform (Heers et al., 2011, 2016) and result in a less effective wing orientation, such that aerodynamic force production would not actually increase much and increasing wingbeat frequency would offer little improvement to performance. Regardless, our simulations suggest that baby and particularly juvenile muscles are strong enough to flap wings with better quality feathers.

### Implications for the evolution of avian flight

The fossil record shows that large, bird-like wings evolved before fully bird-like skeletons (Dececchi et al., 2016; Heers et al., 2016)—and presumably hypertrophied pectoral muscles—were acquired. Traditionally, small pectoral muscles were assumed to preclude non-avian theropods and early birds from producing significant amounts of aerodynamic force via flapping (Ostrom, 1974, 1976, 1979, 1986; Bock, 1986). However, our results reaffirm that animals with small body size do not require hypertrophied flight muscles for flapping behaviors involving the cooperative use of wings and legs. As in juvenile chukars, small, incipiently flight-capable theropods with relatively bird-like wings but less derived musculoskeletal anatomies might have had enough muscle capacity for behaviors like flap-running, flapping jumps, and possibly even brief flight. In species with larger bodied adults [e.g., *Velociraptor* (Turner et al., 2007a)], it is reasonable to hypothesize that juveniles were capable of flapping behaviors unavailable to adults (see Parsons and Parsons, 2015), such that younger animals were more wing-reliant and older animals were more leg-reliant, as in extant peafowl [*Pavo cristatus* (Heers and Dial, 2015)]. Selective pressure for improved wing performance may then have favored paedomorphosis, which would be consistent with the small body size (Turner et al., 2007b; Lee et al., 2014) of paravians and the paedomorphic characteristics [e.g., skulls (Bhullar et al., 2012)] of avialans. The approach outlined here provides a framework for constructing musculoskeletal models of other birds or extinct theropod dinosaurs, and future analyses addressing the relationship between wing vs. muscle limitations across a wide range of body sizes, comparing different behaviors (e.g., WAIR vs. flight), and exploring the effects of muscle origin and insertion positioning would provide great insight into the biomechanics and evolution of avian locomotion.

## Conclusions

Our models and simulations allowed us to estimate muscle function under different combinations of aerodynamic force and kinematics, in order to better examine how muscles, bones, and feathers interact with each other and the environment to accomplish locomotor tasks during bird ontogeny. Although static approaches are limited in their ability to account for dynamic effects such as tendon elasticity, the simulated patterns of muscle activation, shortening vs. lengthening, and force development reported here are broadly similar to patterns previously reported for flap-running and flying birds. A static perspective is thus useful for estimating musculoskeletal biomechanics in flapping chukars, and provides a valuable first start that more dynamic approaches can build upon. Static simulations offer several new insights into development of the avian flight apparatus and, most importantly, suggest that (i) feathers are more limiting than muscles in young birds, likely due to their unique style of growth, and that (ii) small animals do not need large muscles to produce at least moderate amounts of aerodynamic force (>60% of body weight).

## Ethics statement

This study was carried out in accordance with the recommendations of the IACUC guidebook, University of Montana Institutional Animal Care and Use Committee, and the Royal Veterinary College Ethics and Welfare Committee. The protocol was approved by the University of Montana Institutional Animal Care and Use Committee, and the Royal Veterinary College Ethics and Welfare Committee.

## Data availability

All relevant data are contained within the manuscript. The adult musculoskeletal model will be made publicly available in 2019 at https://simtk.org/. Prior to this date all models are available upon request to the primary author.

## Author contributions

AH and JH designed the musculoskeletal modeling and simulation protocol. AH performed dissections, built musculoskeletal models, ran simulations, and analyzed data. JH and JR provided feedback and guidance on modeling and simulation procedures. AH wrote the manuscript. JR and JH reviewed and edited manuscript drafts.

### Conflict of interest statement

The authors declare that the research was conducted in the absence of any commercial or financial relationships that could be construed as a potential conflict of interest.

## Abbreviations

WAIR: wing-assisted incline running.
