# Supplementary material for: Building a Bird: Musculoskeletal Modeling and Simulation of Wing-Assisted Incline Running During Avian Ontogeny
Source: Front Bioeng Biotechnol. 2018 Oct 23;6:140. doi: 10.3389/fbioe.2018.00140 (PMC6205952; doi:10.3389/fbioe.2018.00140)
Supplement: Supplementary file 1 [file Table_1.PDF]

Table S1. Origins and insertions of forelimb muscles

SHOULDER MUSCLES	ORIGIN	INSERTION
**Pectoralis thoracica (Pec)***	*Fleshy*<br>Lateral surface of furcular shaft<br>Membrane between hypocleideum and sternum, and lateral surface of coracoclavicular membrane<br>Ventral 1/3 of keel<br>Lateral rami of sternum and interosseus membranes<br>Lateral surface of sternal elements of thoracic ribs	*Tendon*<br>Ventral surface of deltopectoral crest of humerus
**Pectoralis propatagialis** [x]<br>("included" with PrL)	*Fleshy*<br>Anterolateral surface of belly of Pec, adjacent to its insertion	*Tendon*<br>continuous with tendon of PrL
**Pectoralis abdominalis** [x]	Ventral feather tract	SHC, Pec
**Supracoracoideus, ventral head (Spr)**	*Fleshy*<br>Keel, dorsal to Pec<br>Ventral surface of sternal body<br>Proximal halves of interosseus membranes between lateral rami of sternum	*Tendon*<br>which passes thru triosseal canal to distal end of dorsal surface of deltopectoral crest of humerus, where crest meets posterior surface of humerus
**Supracoracoideus, deep head** [x]<br>("included" with Spr)	*Fleshy*<br>Episternal process (anterior sternum)<br>Ventromedial surface of coracoid<br>Lateral surface of coracoclavicular membrane	*Tendon*<br>which passes thru triosseal canal, medial to Spr, to Tuberculum dorsale on proximal end of dorsal surface of deltopectoral crest of humerus, near anterior edge of crest
**Coracobrachialis anterior (CbA)**	*Tendon*<br>Posteromedial side of acrocoracoid, near articulation with scapula and furcula	*Fleshy*<br>Anterior surface of proximal humerus, adjacent to proximal end of deltopectoral crest and between insertion of Pec and Acrocoracohumeral ligament
**Coracobrachialis posterior (CbP)**	*Fleshy*<br>Sternum, near common base of lateral rami, but mainly from<br>Lateral surface of coracoid	*Tendon*<br>Posterior surface of proximal humerus on Margo caudalis, adjacent to pneumatic foramen
**Subcoracoideus (Sbc)**	*Fleshy*<br>Dorsal surface of sternum near articulation with coracoid, and episternal process near rostrum of sternum<br>Strip along posteromedial surface of coracoid<br>Coracoclavicular membrane	
**Subscapularis (Sbs)**	*Fleshy*<br>Ventral and medial surfaces of scapula, from anterior end to almost mid-bone<br>Medial surface of adjacent coracoid (not modeled)	
**Latissimus dorsi anterior (LtD)**	*Fleshy*<br>Spinous processes of last cervical and first thoracic vertebrae	*Fleshy*<br>Passes between STri & HTri to posterior surface of humerus, ~ 1/3 distance down shaft
**Latissimus dorsi posterior** [x]<br>("included" in LtD, SHC)	*Tendinous*<br>Spinous processes, posterior to LtD and extending to anterior edge of ilium	*Tendinous sheet*<br>attached to LtD, SHC, scapula, and humerus, near shoulder joint
**Scapulohumeralis caudalis (SHC)**	*Fleshy*<br>Dorsal and lateral surfaces of most of shaft of scapula, beginning short distance behind shoulder joint and extending to posterior end of bone	*Tendon*<br>Posterior surface of proximal humerus on bicipital crest, adjacent to pneumatic fossa
**Scapulohumeralis cranialis** [x]<br>("included" in SHC)	*Fleshy*<br>Lateral surface of scapula, short distance behind glenoid fossa and anteroventral to origin of SHC	*Fleshy*<br>Posterior surface of proximal humerus, just distal to pneumatic fossa; separates medial & lateral heads of HTri
**Deltoideus major (DMa)**	*Fleshy*<br>Medial surfaces of clavicular head, scapula, and acrocoracoid, adjacent to triosseal canal; attached to joint capsule and STri	*Tendinous*<br>Large area of posterodorsal surface of humeral shaft
**Deltoideus minor (DMi)**	*Fleshy*<br>Medial surfaces of arocoracoid and scapula, adjacent to triosseal canal	*Tendinous*<br>Passes beneath propatagialis to Tuberculum dorsale of proximal humerus , adjacent to Spr

ELBOW MUSCLES	ORIGIN	INSERTION
**Propatagialis longus (PrL), brevis (PrB)**	*Fleshy*<br>Anteromedial side of acrocoracoid<br>Acrocoracoclavicular ligament	*Tendinous*<br>PrB: anteroproximal surface of EMR<br>PrL: disappears into propatagium, inserting diffusely on ventral surface of wrist (not analyzed)
**Biceps brachii (BiB)**	*Tendinous*<br>Anteroventral surface of proximal humerus, on bicipital crest (short head)<br>Small area on anterior surface acrocoracoid, at coracoclavicular joint (long head; not modeled because firmly connected to short head)	*Tendon*<br>Bifurcates just before inserting on ventral surface of head of radius and adjacent surface of ulna
**Scapulotriceps (STri)**	*Fleshy (scapular origin) or tendinous (humeral origin)*<br>Dorsal and lateral surfaces of scapula, just posterior to glenoid fossa<br>Posterior surface of humeral head (not modeled separately)	*Tendon*<br>Olecranon process of ulna
**Humerotriceps (HTri)**	*Fleshy*<br>Long area along posterior surface of humerus, from pneumotricipital fossa to distal condyles; split into medial and lateral heads by Margo caudalis (but modeled as a single muscle because heads merge)	
**Brachialis (Brc)**	*Fleshy*<br>Small region on anterior surface of humeral shaft, just proximal to distal condyles	*Fleshy*<br>Ventral surface of proximal ulna
**Entepicondylo-ulnaris (Ent)**	*Tendinous*<br>Medial epicondyle of humerus, anterodistal edge, with ProP (fused with ProP proximally)	*Fleshy*<br>Ventral surface of proximal ulna, between insertion of Brc and origin of UMV
**Pronator profundus (ProP)**	*Tendon*<br>Medial epicondyle of humerus, anterodistal edge, with Ent (fused with Ent proximally)	*Somewhat tendinous*<br>Long narrow region on posteroventral surface of radius, mid-shaft
**Pronator sublimis (ProS)**	*Tendon*<br>Medial epicondyle of humerus, just proximal to origin of ProP and Ent	*Fleshy*<br>Long narrow region on ventral surface of distal radius
**Supinator (Sup)**	*Tendon*<br>Middle of lateral epicondyle of humerus	*Mainly fleshy*<br>Extensive region on anterior surface of radius, mid-shaft
**Anconeus (Anc)**	*Tendon*<br>Middle of lateral epicondyle of humerus	*Fleshy*<br>Anterodorsal surface of most of shaft of ulna

WRIST MUSCLES	ORIGIN	INSERTION
**Flexor carpi ulnaris (FCU)**	*Tendon*<br>Medial epicondyle of humerus, posterior edge	*Tendon*<br>Proximal surface of ulnare, near articulation with ulna
**Flexor digitorum profundus (FDP)**	*Fleshy*<br>Anteroventral surface of proximal ulna, bordered by Brc, Ent, and EPL	*Tendon*<br>which passes through retinaculum on ventral surface of wrist, joins tendon of FDS ~ midway down metacarpus, and inserts proximally on anteroventral edge of Digit II.2
**Flexor digitorum sublimis (FDS)**	*Fleshy*<br>Anterior fold of fibrous sheet enclosing FDS and FCU	*Tendon*<br>which anchors to ventral surface of ulnare, joins tendon of FDP ~ midway down metacarpus, and inserts on anterior edge of Digit II.1 and sometimes Digit II.2
**Ulnimetacarpalis ventralis (UMV)**	*Fleshy*<br>Extensive region on ventral surface of mid- to distal-ulna	*Tendon*<br>Passes through wrist and inserts on anterodorsal edge of carpometacarpus, proximal to extensor process of Metacarpal I
**Extensor metacarpi radialis (EMR)**	*Partly fleshy*<br>Lateral epicondyle of humerus, proximal end	*Tendon*<br>which slides over radiale and inserts on anterior edge of extensor process of Metacarpal I; fuses with tendon of EPL
**Extensor digitorum communis (EDC)**	*Tendon*<br>Middle of lateral epicondyle of humerus	*Tendon*<br>which passes through retinaculum on anterodorsal surface of ulna, then splits and inserts on (i) small tubercle on posterior edge of Digit I.1, ~ 1/3 of distance from proximal end (not modeled), and (ii) anterior edge of base of Digit II.1
**Extensor carpi ulnaris (ECU)**	*Tendon*<br>Middle of lateral epicondyle of humerus	*Tendon*<br>which passes through retinaculum on anterodorsal surface of ulna, and inserts on dorsal surface of intermetacarpal process of Metacarpal II
**Extensor pollicis longus (EPL)**	*Fleshy*<br>Small head on posterior surface of shaft of proximal radius (not modeled) + large head on anterodorsal surface of proximal ulna; bellies fuse ~ midway along forearm	*Tendon*<br>which fuses completely with tendon of EMR, at wrist, and inserts on anterior edge of extensor process of Metacarpal I
**Extensor indicis longus (EIL)**	*Fleshy*<br>Posterior surface of radius, mid-shaft	*Tendon*<br>which passes through retinaculum on dorsal surface of ulna and inserts on distal end of anterior edge of Digit II.2

Origins and insertions are based on dissection of multiple adult and juvenile chukars, and confirmed with (Hudson and Lanzilloti 1964); some small differences present but otherwise similar. Bone terminology follows (Baumal & Witmer 1993) and (Ghetie 1976) (more applicable to galliforms).

Directional definitions:
(M = medial, L = lateral)

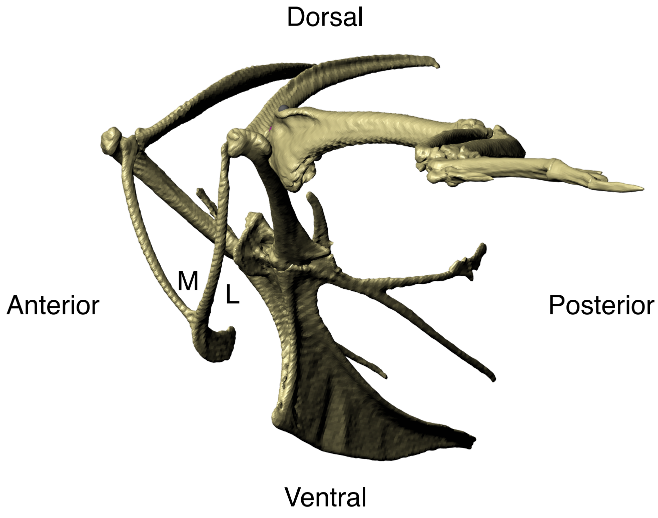

Notes on specific muscles:

For muscles with broad attachment sites, we positioned the origin or insertion in the middle of the attachment site for parallel muscles (ex: LtD), or at the end of the central tendon when present (SHC, Spr). Because muscle fascicles converge when crossing joints — even for muscles with broad origins — they have ~ similar moment arms. Simulations using a single muscle or multiple muscles thus yield comparable results (but see Fig. S5 for discussion).

* For modeling purposes, we did not distinguish between pars sternobrachialis (SB) and pars thoracobrachialis (TB). SB and TB are innervated by separate branches of the brachial plexus (Kaplan 1987) and partially separated by an internal aponeurosis. However, both muscles are activated ~ simultaneously (Dial 1992), and TB fascicles merge with the tendon of SB before inserting on the humerus (Dial 1988). Future analyses could examine these complexities of the pectoralis, but here we modeled it as a single muscle.

x muscles not included in model, either due to small size or because their functional effects are "included" in other muscles (e.g., the very small Scapulohumeralis cranialis is included as part of the Scapulohumeralis caudalis)

Citations:

Baumel, J. J., and L. M. Witmer. 1993. Osteologia. Pp. 45–132 *in* J. J. Baumel, A. S. King, J. E. Breazile, H. E. Evans, and J. C. Vanden Berge, eds. Handbook of Avian Anatomy: Nomina Anatomica Avium. Nuttall Ornithological Club, Cambridge MA.

Dial, K. P. 1992. Activity Patterns of the Wing Muscles of the Pigeon (Columba livia) During Different Modes of Flight. Journal of Experimental Zoology 262:357–373.

Dial, K. P., S. R. Kaplan, G. E. J. Goslow, and F. A. J. Jenkins. 1988. A functional analysis of the primary upstroke and downstroke muscles in the domestic pigeon (Columba livia) during flight. Journal of Experimental Biology 134:1–16.

Ghetie, V., S. Chitescu, V. Cotofan, and A. Hildebrand. 1976. Anatomical Atlas of Domestic Birds. Editura Academiei Republicii Socialiste Romania, Bucharest, Romania.

Hudson, G. E., and P. J. Lanzillotti. 1964. Muscles of the Pectoral Limb in Galliform Birds. American Midland Naturalist 71:1–113.

Kaplan, S. R. 1987. Functional heterogeneity in the pigeon pectoralis. Northern Arizona University, Flagstaff.
