# Supplementary material for: Building a Bird: Musculoskeletal Modeling and Simulation of Wing-Assisted Incline Running During Avian Ontogeny
Source: Front Bioeng Biotechnol. 2018 Oct 23;6:140. doi: 10.3389/fbioe.2018.00140 (PMC6205952; doi:10.3389/fbioe.2018.00140)
Supplement: Supplementary file 3 [file Table_3.PDF]

**Table S3. Aerodynamic inputs for simulation.** Horizontal and vertical aerodynamic forces applied to brachial (B), antebrachial (A), and manual (M) wing segments for each model. Values are based on wing-assisted incline running on 65° inclines (data from (Heers et al., 2011)); colors match colors of wing segments in Figure 3.

| Age | | Total aerodynamic force | | Coefficient of resultant force, 45° angle of attack ($C_R$) | Angle of force (deg from horizontal) | Surface area of wing segment (S; $m^2$) | Distance btwn center of wing segment and shoulder jt (r; m) | Angular velocity (Ω; radians) | Trans-lational velocity ($V_T$; m/s) | Proportion of total force ∝ $S[(\Omega r)^2+V_T^2]$ | Total force (N) | Horizontal force (N) | Vertical force (N) |
|---|---|---|---|---|---|---|---|---|---|---|---|---|---|
| | | 2 wings, % body wt | 1 wing, N | | | | | | | | | | |
| Baby: 7-8 days | B | 8.68 | 1.47E-02 | 1.69 | 72 | 1.87E-04 | 7.30E-03 | 68.80 | 0.62 | 0.012 | 1.82E-04 | 5.63E-05 | 1.73E-04 |
| | A | | | | | 7.10E-04 | 1.93E-02 | | | 0.158 | 2.33E-03 | 7.21E-04 | 2.22E-03 |
| | M | | | | | 7.27E-04 | 4.73E-02 | | | 0.829 | 1.22E-02 | 3.78E-03 | 1.16E-02 |
| Juvenile: 18-20 days | B | 31.15 | 1.30E-01 | 1.40 | 60 | 9.50E-04 | 1.06E-02 | 66.29 | 1.18 | 0.013 | 1.63E-03 | 8.16E-04 | 1.41E-03 |
| | A | | | | | 3.55E-03 | 3.93E-02 | | | 0.204 | 2.64E-02 | 1.32E-02 | 2.28E-02 |
| | M | | | | | 2.83E-03 | 9.30E-02 | | | 0.784 | 1.02E-01 | 5.08E-02 | 8.80E-02 |
| Adult: >100 days | B | 60.48 | 1.48E+00 | 1.97 | 57 | 3.24E-03 | 2.39E-02 | 60.95 | 1.57 | 0.015 | 2.22E-02 | 1.21E-02 | 1.86E-02 |
| | A | | | | | 8.23E-03 | 7.27E-02 | | | 0.182 | 2.71E-01 | 1.47E-01 | 2.27E-01 |
| | M | | | | | 9.63E-03 | 1.47E-01 | | | 0.803 | 1.19E+00 | 6.48E-01 | 9.98E-01 |
