# Supplementary material for: Building a Bird: Musculoskeletal Modeling and Simulation of Wing-Assisted Incline Running During Avian Ontogeny
Source: Front Bioeng Biotechnol. 2018 Oct 23;6:140. doi: 10.3389/fbioe.2018.00140 (PMC6205952; doi:10.3389/fbioe.2018.00140)
Supplement: Supplementary file 4 [file Table_4.pdf]

Table S4. Reserve actuators. Reserve actuators, which contribute torque if muscles are not strong enough, were set at an optimal torque representing 50% of the maximum torque (based on inverse dynamics) at a joint in a given direction, for each model. Maximum joint torques, and hence the magnitudes of reserve actuators, increase from baby to juvenile to adult. Long-axis rotation torques are small compared to other motions, and, at the elbow and wrist, abduction-adduction torques are generally greater than those for flexion-extension, reflecting the high inertial torques associating with flapping and aerodynamic force production. Similarly, at the shoulder, elevation-depression torques are greatest in the adult and juvenile, but protraction-retraction is greatest in the baby; young chicks tend to flap in a more craniocaudal direction than older birds (Heers et al., 2011).

	Shoulder moments (N m)			Elbow moments (N m)			Wrist moments (N m)		
	Baby	Juvenile	Adult	Baby	Juvenile	Adult	Baby	Juvenile	Adult
elevation-depression (shoulder) or extension-flexion (elbow, wrist)	0.000955	0.0199	0.193	0.000456	0.00958	0.0606	0.000106	0.00201	0.0198
protraction-retraction (shoulder) or abduction-adduction (elbow, wrist)	0.00104	0.00901	0.161	0.000865	0.00730	0.104	0.000298	0.00496	0.0337
pronation-supination	0.000933	0.00787	0.0994	0.000296	0.00536	0.0427	0.0000331	0.000339	0.00497
