# Supplementary material for: Building a Bird: Musculoskeletal Modeling and Simulation of Wing-Assisted Incline Running During Avian Ontogeny
Source: Front Bioeng Biotechnol. 2018 Oct 23;6:140. doi: 10.3389/fbioe.2018.00140 (PMC6205952; doi:10.3389/fbioe.2018.00140)
Supplement: Supplementary file 5 [file Table_5.PDF]

Table S5. Reserve actuators: maximum percentage of total shoulder moment during one wingstroke.

	Elevation-Depression	Protraction-Retraction	Pronation-Supination
Baby	28	23	21
Juvenile	68	35	8
Adult	74	49	5
