# Supplementary material for: Building a Bird: Musculoskeletal Modeling and Simulation of Wing-Assisted Incline Running During Avian Ontogeny
Source: Front Bioeng Biotechnol. 2018 Oct 23;6:140. doi: 10.3389/fbioe.2018.00140 (PMC6205952; doi:10.3389/fbioe.2018.00140)
Supplement: Supplementary file 6 [file Table_6.PDF]

Table S6. Sensitivity Analysis of Model Uncertainties

Properties that were not varied:

- Anatomical, kinematic, or kinetic variables that were measured on specimens or recorded during experiments (e.g., muscle mass (proportional to peak isometric muscle force), fiber length, raw wing kinematics, magnitude of aerodynamic force)
- Dynamic muscle properties, which do not affect static simulations (e.g., maximum contractile velocity, activation and deactivation time constants, force-velocity curves, damping)

Properties that were varied: morphological, kinematic, or kinetic features with multiple ways of being represented, in terms of model construction and/or simulation

---

Solid lines = baseline model (adult chukar, static optimization with muscle physiology “off”)
Dashed lines = adjusted model (adult chukar, static optimization with muscle physiology “off”)

<table>
<tr><th colspan="2">Parameter Adjusted</th><th>Activation of Pectoralis and Supracoracoideus muscles</th><th>Reserve torques at shoulder joint</th></tr>
<tr><td>Skeletal anatomy</td><td>Location of shoulder joint</td><td>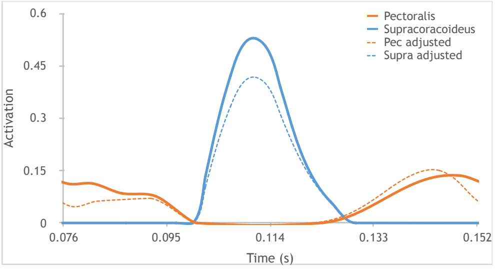
</td><td>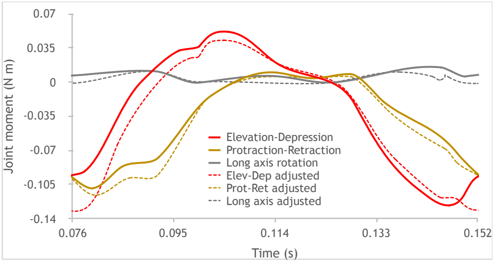
</td></tr>
<tr><td rowspan="2">Muscle geometry</td><td>Pectoralis wrapping surface</td><td>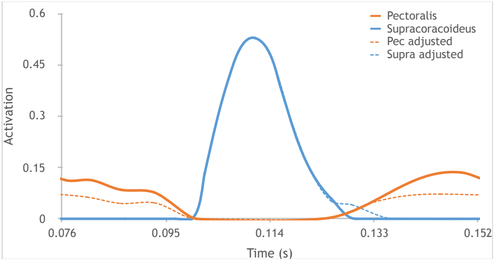
</td><td>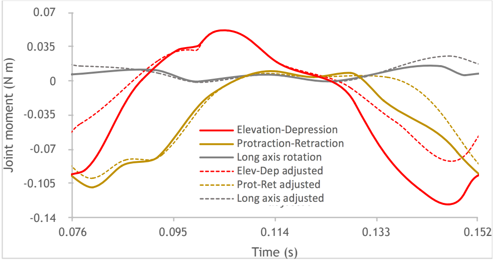
</td></tr>
<tr><td>Pectoralis modeled as 3 muscles</td><td>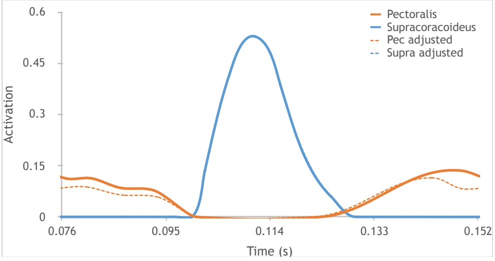
</td><td>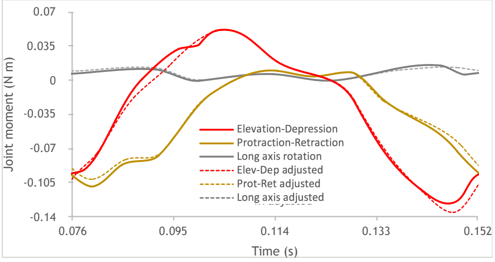
</td></tr>
</table>

<table>
<tr><td rowspan="1">Distribution of wing mass</td><td>With wing feathers</td><td>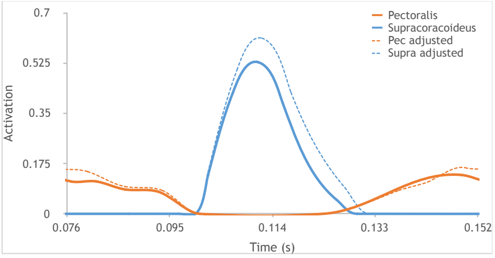</td><td>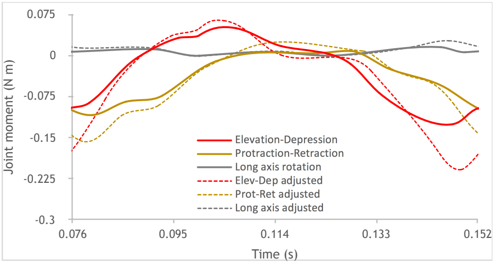</td></tr>
<tr><td rowspan="4">Flapping kinematics</td><td>Level of filtering: iterative smoothing</td><td>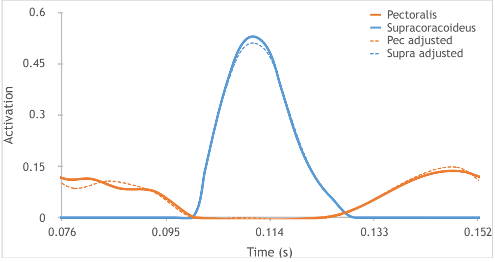</td><td>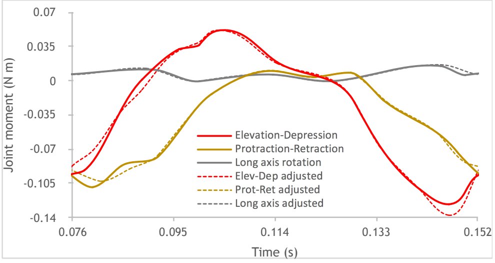</td></tr>
<tr><td>Level of filtering: sine wave</td><td>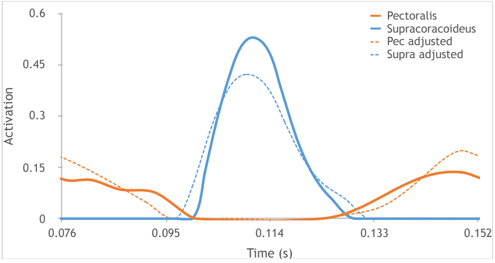</td><td>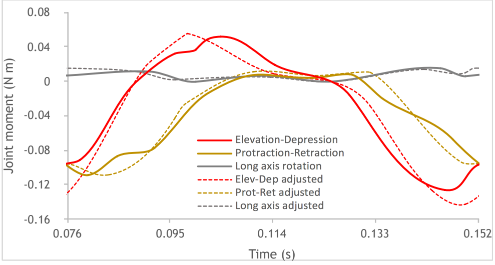</td></tr>
<tr><td>Duration of wing turnaround</td><td>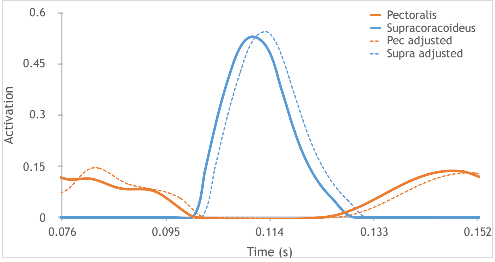</td><td>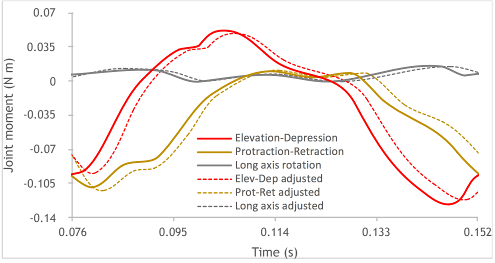</td></tr>
<tr><td>Body kinematics prescribed (driven by legs)</td><td>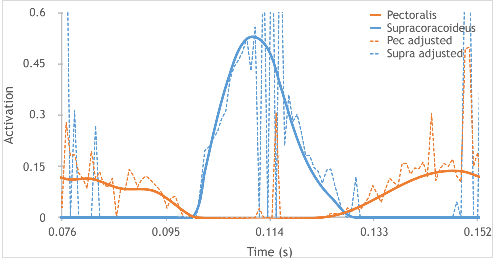</td><td>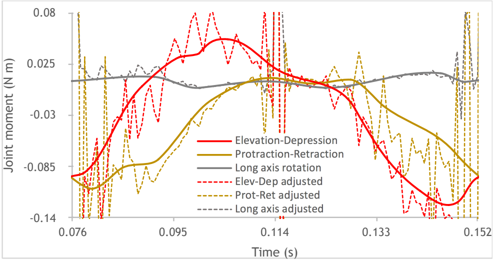</td></tr>
</table>

<table>
<tr>
<td rowspan="2">Flapping kinematics cont.</td>
<td>Body kinematics “free” (driven by wings and legs)</td>
<td>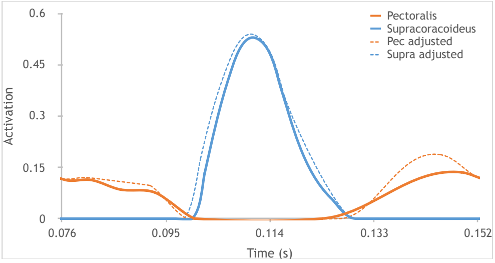
</td>
<td>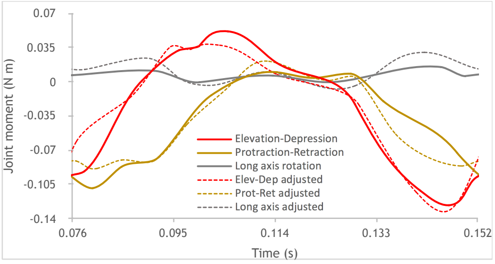
</td>
</tr>
<tr>
<td>Wing joint translations prescribed (driven by joint geometry)</td>
<td>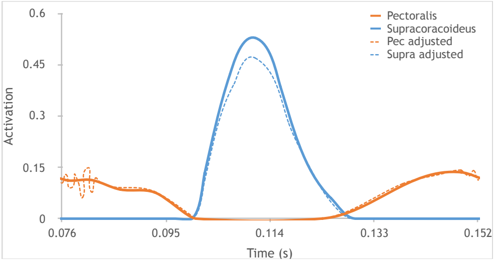
</td>
<td>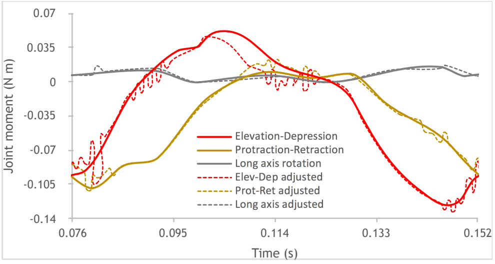
</td>
</tr>
<tr>
<td>External loads</td>
<td>Location of aerodynamic force</td>
<td>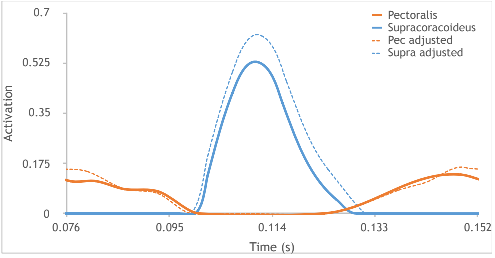
</td>
<td>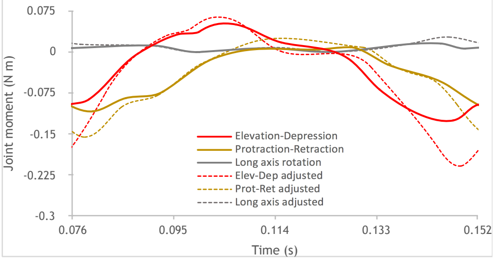
</td>
</tr>
</table>

<u>*Explanations*</u>:

Location of shoulder joint. <u>Baseline model</u>, shoulder joint location defined anatomically: centered between head of humerus and glenoid facet (see Heers et al. 2016 and Baier et al. 2013). <u>Adjusted model</u>, shoulder joint location set at center of rotation (determined from wing kinematics used in simulation): shifted 1.1 mm distally *into* humerus (2% of humeral length), which actually reduces the moment arms of the pectoralis and does not reduce reserve torques.

Pectoralis wrapping surface. <u>Baseline model</u>, pectoralis prevented from passing through the coracoid, scapula, and humerus by a wrapping surface anchored to humerus, resulting in substantial movement in the path of the muscle during flapping. <u>Adjusted model</u>, pectoralis prevented from passing through the coracoid, scapula, and humerus by a wrapping surface anchored to sternum, resulting in less movement in the path of the muscle and substantially reducing - but not eliminating - reserve torques.

Pectoralis: 1 or 3 muscles? <u>Baseline model</u>, pectoralis modeled as single muscle, at the center of the volume of the muscle. <u>Adjusted model</u>, pectoralis modeled as 3 muscles (average activation plotted; see Fig. S5). Does not reduce reserve torques.

Centers of Mass of wing segments: with or without wing feathers? <u>Baseline model</u>, COM calculated without wing feathers; COM’s of wing segments are close to the wing bones. <u>Adjusted model</u>, COM calculated with wing feathers; COM’s of wing segments are close to the quarter-chord positions. Increases contribution by reserve torques, possibly because the model cannot account for changes in COM positioning when the wing feathers tuck in during the upstroke.

Level of filtering. Baseline model, kinematics smoothed using a Loess function, equivalent to a low pass filter, prior to running simulations; kinematics additionally filtered at 53 Hz during simulations. Adjusted model, kinematics iteratively smoothed to minimize kinematic discontinuities in velocity and acceleration, prior to running simulations, OR kinematics fit with a sine wave, prior to running simulations; for both cases, kinematics additionally filtered at 53 Hz during simulations. Changes activation of pectoralis and particularly supracoracoideus; does not reduce reserve torques.

Duration of wing turnaround. Baseline model, beginning and end of one wingbeat determined kinematically, as the maximum height of the tip of the manus (see Heers et al. 2016 and Baier et al. 2013); this wingbeat was duplicated multiple times to produce a sequence of 3 wingbeats for simulations, and the middle of these 3 wingbeats was analyzed. Adjusted model, 1.5 ms (~2% of the original wingbeat duration) was added to the end of each wingbeat, with kinematics kept constant over the 1.5 ms duration, to account for the observation that our chukars sometimes kept the manus elevated momentarily before beginning another downstroke (data from Baier et al. 2013). Note that offset in baseline versus adjusted results is due to a shift in wingbeat timing; reserve torques not reduced.

Body kinematics prescribed (driven by legs). Baseline model, all joints "locked" into place except wing rotations (i.e., rotational movements at shoulder, elbow, and wrist). Adjusted model, motion at body joints (pelvis / whole body, sternum, coracosternal) prescribed: allows body to move as measured during WAIR (without requiring leg or trunk muscles), to account for small changes in wing orientation due to changes in body pitch. Results noisy due to kinematic noise in body kinematics, but does not reduce reserve torques.

Body kinematics "free" (driven by wings and legs). Baseline model, all joints "locked" into place except wing rotations (i.e., rotational movements at shoulder, elbow, and wrist). Adjusted model, body kinematics included but driven by additional reserve actuators that were created to take the place of leg and trunk muscles not included in the model. Collectively, these changes account for the fact that whole body motion is driven at least to a small extent by the wings (i.e., "Body kinematics prescribed" assumes that whole body motion is driven entirely by the legs).

Wing translations prescribed (driven by joint geometry). Baseline model, wing translations locked into position (average position over one wingbeat). Adjusted model, wing translations prescribed (range of motion at shoulder joint = 1.5-8.0 mm), such that bone rotation is driven by muscles and bone translation is driven by joint geometry.

Location of aerodynamic force. Baseline model, aerodynamic forces proximodistally centered in each wing segment, at the quarter chord length. Adjusted model, aerodynamic forces proximodistally positioned in alignment with the center of mass (COM) of each wing segment, at the quarter chord length (which aligns very closely with COM position); magnitudes adjusted to match new location.

Simulation insights:

- Run simulations using all shoulder muscles; using just the pectoralis and supracoracoideus inflates their activations and occasionally results in simulation failure
- Run simulations using multiple (3) wingbeats — the beginning and end of static optimization does not yield accurate results, so the middle wingbeat should be analyzed
