# Supplementary material for: Building a Bird: Musculoskeletal Modeling and Simulation of Wing-Assisted Incline Running During Avian Ontogeny
Source: Front Bioeng Biotechnol. 2018 Oct 23;6:140. doi: 10.3389/fbioe.2018.00140 (PMC6205952; doi:10.3389/fbioe.2018.00140)
Supplement: Supplementary file 7 [file Table_7.PDF]

Table S7. Peak and average muscle activations. Values are means ± standard deviations. For muscle activations, 0 is minimum (muscle not active) and 1 is maximum (100% of the muscle is fully active).

	Age Class	All Muscles		Shoulder Muscles		Elbow Muscles		Wrist Muscles	
		average	SD	average	SD	average	SD	average	SD
Average activations	baby	0.082	0.078	0.038	0.040	0.11	0.091	0.10	0.079
	juvenile	0.069	0.067	0.055	0.056	0.097	0.084	0.049	0.045
	adult	0.080	0.088	0.058	0.058	0.099	0.11	0.081	0.094
Peak activations	baby	0.34	0.27	0.20	0.23	0.41	0.29	0.39	0.27
	juvenile	0.30	0.24	0.21	0.21	0.42	0.29	0.25	0.19
	adult	0.27	0.26	0.18	0.19	0.33	0.27	0.28	0.31
