# Supplementary material for: Building a Bird: Musculoskeletal Modeling and Simulation of Wing-Assisted Incline Running During Avian Ontogeny
Source: Front Bioeng Biotechnol. 2018 Oct 23;6:140. doi: 10.3389/fbioe.2018.00140 (PMC6205952; doi:10.3389/fbioe.2018.00140)
Supplement: Supplementary file 8 [file Table_8.pdf]

Table S8 (parts A, B, C). Muscle functions based on muscle moment arms and co-occurrence of activation and kinematics.

Behaviors: WAIR = wing-assisted incline running, AF = ascending flight. Kinematics: DEP = depression, ELEV = elevation, RET = retraction, PROT = protraction, PRO = pronation, SUP = supination, FLEX = flexion, EXT = extension, ADD = adduction, ABD = abduction; DS = downstroke, US = upstroke. Superscripts: A = adult model (>100 days), J = juvenile model (18-20 days), B = baby model (7-8 days); if no superscript, kinematic rotation occurs in all models. Text color, potential function column: black = consistent w/ previously suggested function, red = inconsistent w/ previously suggested function, blue = no previously suggested function; bold = main function. Cell color, right column: green = DS muscle or muscle not active during DS-US transition, blue = US muscle, active during DS-US transition. **: biarticular muscle. *: not tested here (digit kinematics).

A

	Muscle	EMG timing (colored lines = WAIR simulations, Adult, Juvenile, or Baby model; gray bars = AF, from Dial 1992)	Moment arms (Adult solid lines, Juvenile large dash, Baby small dash) + timing of activity (WAIR, simulated; AF from Dial 1991 & 1992) + kinematics (from Heers et al. 2016)		Potential function based on moment arms and phys x-sectional area (Table 2) (black: consistent with previous data, blue: no previous data, red: inconsistent with previous data)		Relative importance (average % torque from Table 2)		Reason for discrepancy with previous work (Dial 1992, Poore 1997, Biewener 2011, Berg & Biewener 2012) on flight?	Suggested function based on moment arms and co-occurrence of EMG and kinematics; green = downstroke muscle, blue = upstroke muscle
			WAIR	AF	WAIR	AF (adult only)	WAIR	AF (adult only)		
SHOULDER	Pectoralis	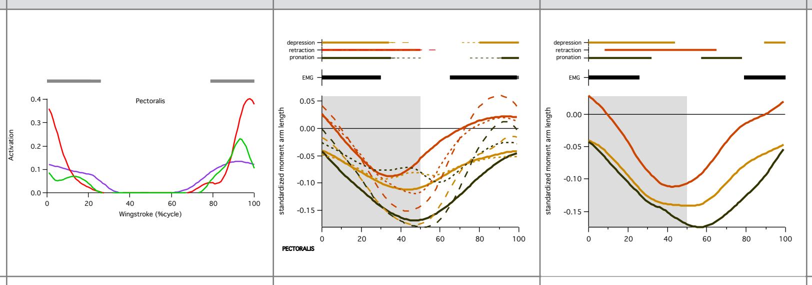			DEP<br>RET or PROT<br>PRO	same	97-100<br>52-59, 19-24<br>73-79	96<br>51, 13<br>76	Consistent	**decelerate ELEV -> DEP;<br>PROT -> RET;<br>decelerate SUP -> PRO**
	Supracoracoideus	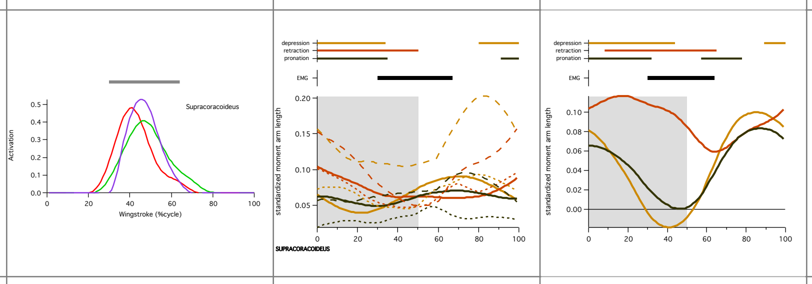			ELEV<br>PROT<br>SUP	same	54-75<br>67-74<br>84-91	50<br>80<br>72	Consistent	**decelerate DEP + RET + PRO -> ELEV + SUP**
	Coracobrachialis posterior	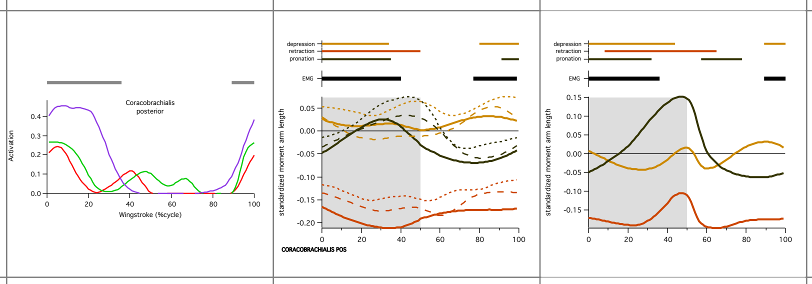			ELEV or DEP^{J}<br>RET<br>SUP or PRO	mainly DEP<br>RET<br>SUP or PRO	x, x<br>10-23<br>4-12, x	x<br>19<br>38, x	WAIR kinematics: humerus kept elevated -> ELEV instead of DEP 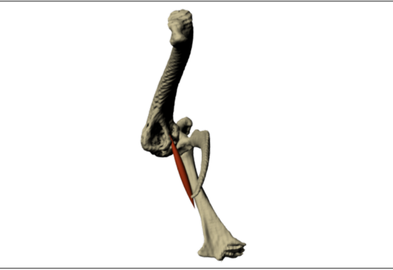	DEP (AF) or opposes DEP (AF, WAIR);<br>**decelerate PROT -> RET**;<br>PRO or opposes PRO
	Coracobrachialis anterior	Very low activation	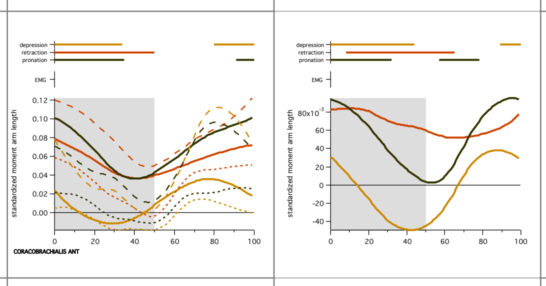		DEP or ELEV<br>PROT<br>SUP or PRO^{B}	same	x, x<br>x<br>x, x	x, x<br>x<br>x	No previously suggested functions	does not seem to contribute much — mainly stabilization
	Subcoracoideus	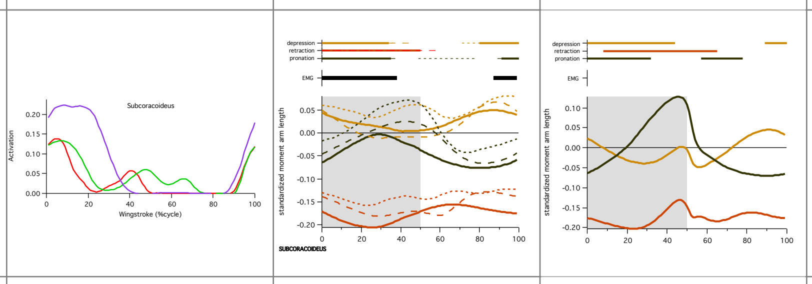			ELEV or DEP^{J}<br>RET<br>PRO or SUP^{BJ}	ELEV or DEP<br>RET<br>PRO or SUP	x, x<br>6-11<br>x, x	x, x<br>9<br>x, 16	No previously suggested functions	opposes DEP;<br>**decelerate PROT -> RET**;<br>mainly PRO
	Latissimus dorsi	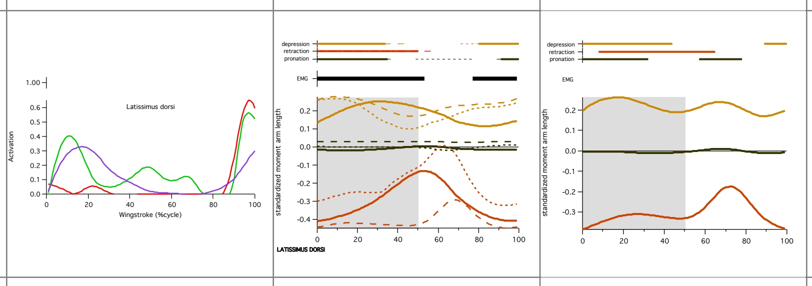			ELEV<br>RET<br>PRO or SUP	same	8-23<br>14-26<br>x, x	19<br>12<br>x, x	No previously suggested functions	**opposes DEP -> ELEV;<br>decelerate PROT -> RET**
	Scapulohumeralis caudalis	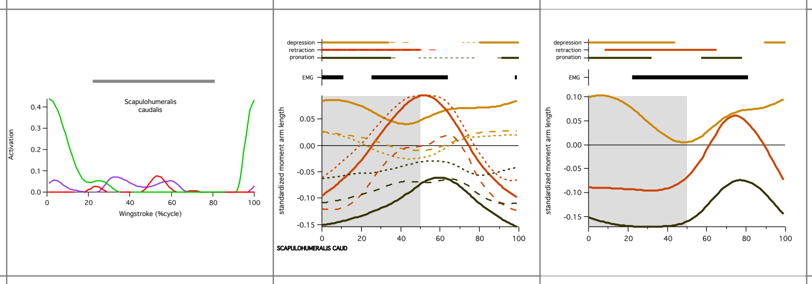			ELEV or DEP^BJ<br>RET or PROT<br>PRO	same	4-21, x<br>17-22, 5-30<br>17-30	24<br>18, 14<br>21	WAIR kinematics: humerus very retracted -> PROT instead of RET 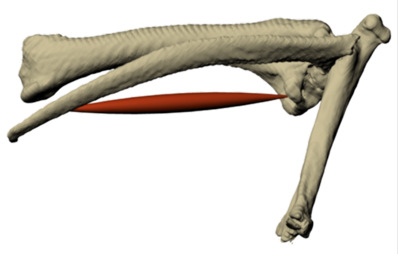	**oppose DEP^{A} -> ELEV^{A} (esp. WAIR);<br>RET -> oppose RET -> PROT;<br>mainly PRO or oppose PRO**
	Subscapularis	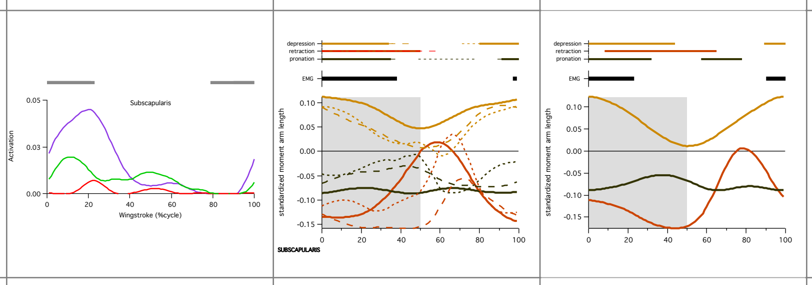			ELEV<br>Mainly RET<br>PRO	ELEV<br>RET<br>PRO	x<br>x<br>x	x<br>x<br>x	Disagree w/ previous work: muscle pulls "down", but elevates an already elevated humerus 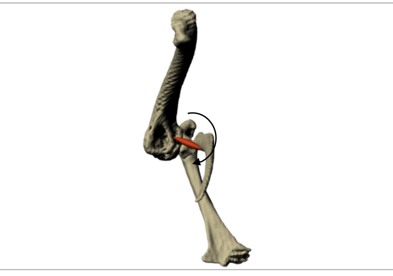	does not seem to contribute much:<br>opposes DEP;<br>decelerate PROT -> RET;<br>decelerate SUP (AF) -> PRO
	Deltoideus major	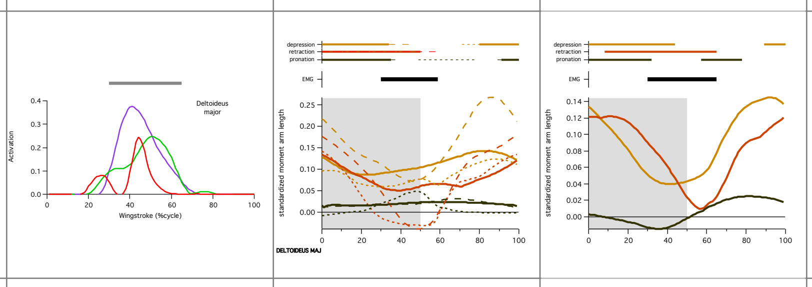			ELEV<br>PROT or RET^BJ<br>SUP	ELEV<br>PROT<br>PRO or SUP	4-6<br>3-5, x<br>x	7<br>x<br>x, x	WAIR kinematics: humerus very retracted -> PROT instead of RET 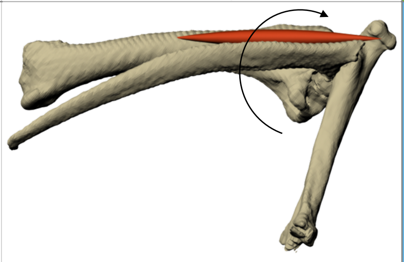	**decelerate DEP -> ELEV;<br>opposes RET^{A} -> PROT^{A} (WAIR);**<br>decelerate PRO -> SUP (WAIR), or opposes long axis rotation (AF)
	Tensor propatagialis brevis	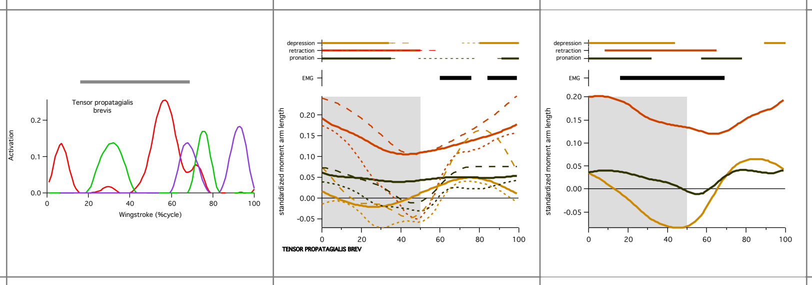			DEP or ELEV<br>PROT or RET^{B}<br>SUP or PRO^{B}	same	x, x<br>7-12, x<br>5-6, x	x, x<br>9<br>x	No previously suggested functions (at shoulder); note that SIMM cannot account for the role of the propatagialis in tensing the propatagium	ELEV^{A} (WAIR) or DEP (AF), stabilization;<br>**PROT (WAIR) or opposes RET (AF)**<br>SUP or opposes PRO (AF)
	Scapulotriceps	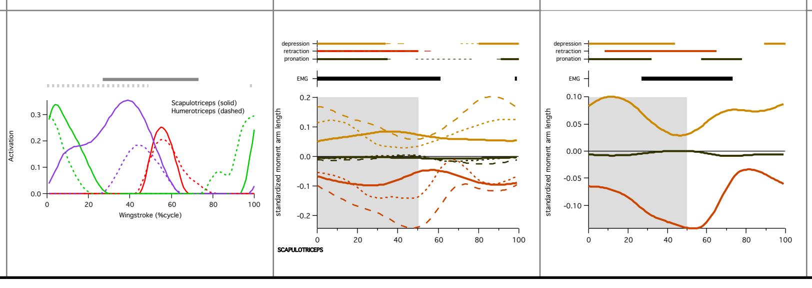			ELEV<br>RET<br>PRO or SUP^{BJ}	same	4-7<br>5-11<br>x, x	7<br>x<br>x	Consistent	**opposes DEP -> ELEV;<br>RET**<br>~ no long axis rotation

Table S8 (parts A, B, C). Muscle functions based on muscle moment arms and co-occurrence of activation and kinematics.

Behaviors: WAIR = wing-assisted incline running, AF = ascending flight. Kinematics: DEP = depression, ELEV = elevation, RET = retraction, PROT = protraction, PRO = pronation, SUP = supination, FLEX = flexion, EXT = extension, ADD = adduction, ABD = abduction; DS = downstroke, US = upstroke. Superscripts: A = adult model (>100 days), J = juvenile model (18-20 days), B = baby model (7-8 days); if no superscript, kinematic rotation occurs in all models. Text color, potential function column: black = consistent w/ previously suggested function, red = inconsistent w/ previously suggested function, blue = no previously suggested function; bold = main function. Cell color, right column: green = DS muscle or muscle not active during DS-US transition, blue = US muscle, active during DS-US transition. **: biarticular muscle. *: not tested here (digit kinematics).

B

	Muscle	EMG timing (colored lines = WAIR simulations, Adult, Juvenile, or Baby model; gray bars = AF, from Dial 1992)	Moment arms (Adult solid lines, Juvenile large dash, Baby small dash) + timing of activity (WAIR, simulated; AF from Dial 1991 & 1992) + kinematics (from Heers et al. 2016)		Potential function based on moment arms and phys x-sectional area (Table 2) (black: consistent with previous data, blue: no previous data, red: inconsistent with previous data)		Relative importance (average % torque from Table 2)		Reason for discrepancy with previous work (Dial 1992, Poore 1997, Biewener 2011, Berg & Biewener 2012) on flight?	Suggested function based on moment arms and co-occurrence of EMG and kinematics; green = downstroke muscle, blue = upstroke muscle
			WAIR	AF	WAIR	AF (adult only)	WAIR	AF (adult only)		
ELBOW	Tensor propatagialis brevis	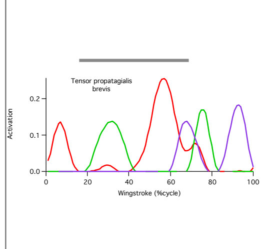	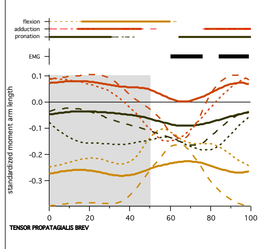	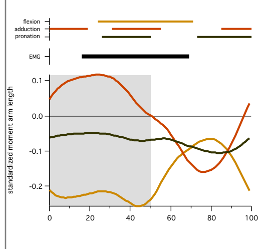	FLEX<br>ADD[BJ] or ABD<br>PRO	FLEX<br>ADD or ABD<br>PRO	39-49<br>15-16, 19-24<br>47-55	37<br>17, 33<br>53	Consistent; note that SIMM cannot account for the role of the propatagialis in tensing the propatagium	**FLEX (AF) or opposes FLEX (WAIR);**<br>**mainly opposes ADD and ABD (WAIR, AF);**<br>PRO or opposes SUP
	Biceps brachii	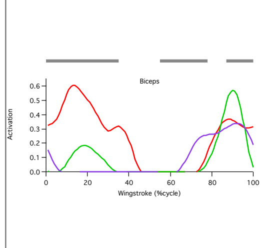	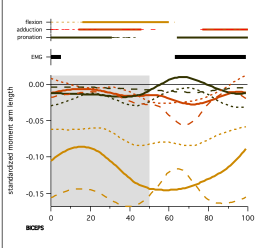	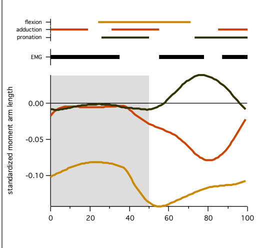	FLEX<br>ADD or ABD[B]<br>PRO or SUP[A]	same	22-32<br>6-7, x<br>5-17, 5	32<br>9<br>6, 19	Consistent	**opposes EXT, or FLEX (AF)**<br>ADD or opposes ABD;<br>SUP (AF) or opposes PRO (AF)
	Scapulotriceps	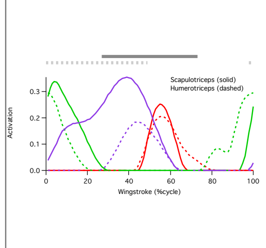	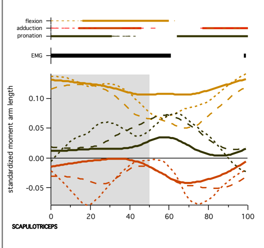	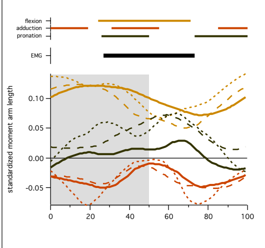	EXT<br>ADD<br>SUP or PRO[B]	EXT<br>ADD<br>SUP or PRO	29-43<br>6-11<br>11-26, x	41<br>9<br>8, 8	Consistent	**opposes FLEX, or EXT (WAIR)**;<br>ADD or opposes ABD;<br>opposes PRO -> SUP
	Humerotriceps	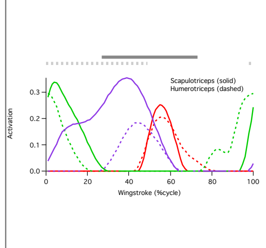	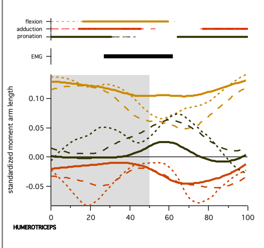	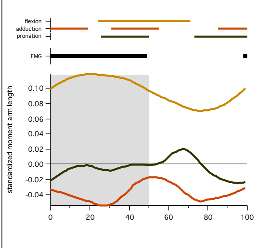	EXT<br>ADD<br>PRO[A] or SUP	same	33-48<br>10-14<br>x, 4-21	45<br>12<br>7, 6	Consistent	**EXT (AF) -> opposes FLEX;**<br>ADD or opposes ABD<br>SUP or opposes PRO (AF)
	Brachialis	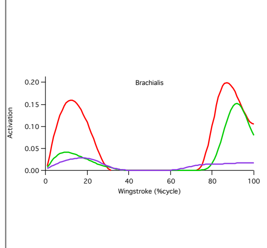	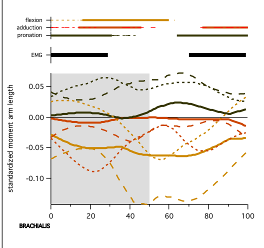	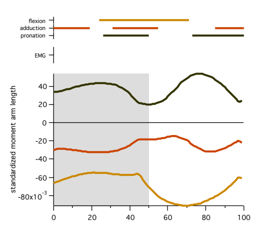	FLEX or EXT[B]<br>ADD[BJ>A]<br>SUP[BJ>A]	same	x, x<br>x<br>x	x<br>x<br>x	No previously suggested functions	opposes EXT -> FLEX;<br>opposes PRO
	Pronator sublimis, profundus	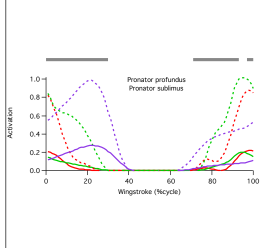	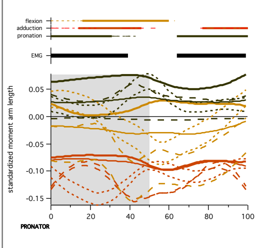	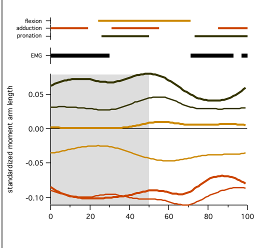	FLEX or EXT[AB]<br>ADD<br>SUP	FLEX<br>ADD<br>SUP	8-18, x<br>42-58<br>3-41	12<br>46<br>44	Disagree: activated during PRO but actually stabilizes against PRO; misnomer from human anatomy. Bird position left, human position right. 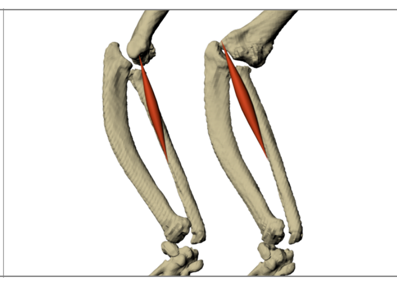	P. profundus: mainly EXT; P. sublimis: FLEX or opposes EXT<br>**mainly ADD**;<br>**opposes PRO, or SUP (AF)**
	Entepicondylo-ulnaris	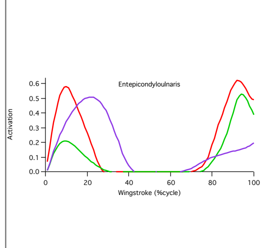	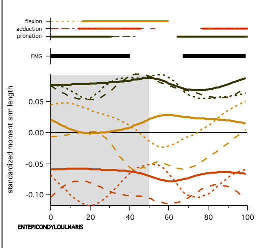	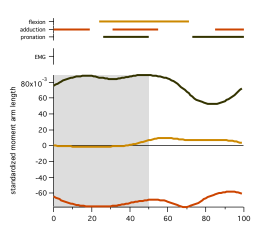	EXT[BA] or FLEX[BJ]<br>ADD<br>SUP	same	x, x<br>7-12<br>14-21	x<br>7<br>20	No previously suggested functions	EXT;<br>**ADD or opposes ABD;**<br>**opposes PRO**
	Supinator	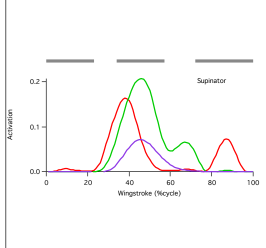	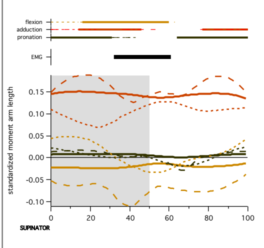	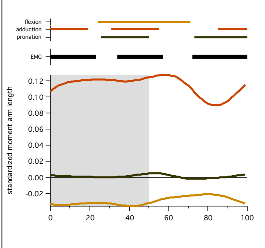	FLEX or EXT[B]<br>ABD<br>SUP or PRO[BJ]	same	x, x<br>5-10<br>x, x	x<br>12<br>x	Consistent; PRO in B and J models is only brief and occurs when the muscle is not activated	FLEX<br>**ABD or opposes ADD**
	Anconeus	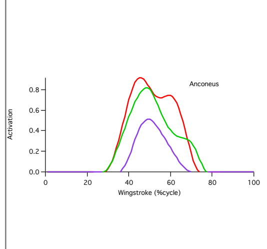	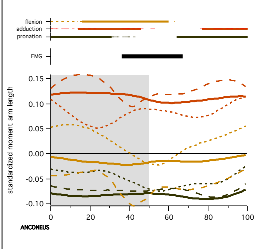	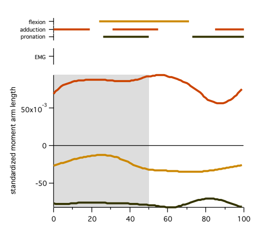	FLEX[AJ>B] or EXT[B]<br>ABD<br>PRO	same	x, x<br>25-32<br>22-39	x<br>28<br>33	No previously suggested functions	FLEX;<br>**ABD or opposes ADD;**<br>**opposes SUP**
	Flexor carpi ulnaris**	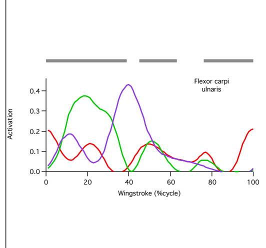	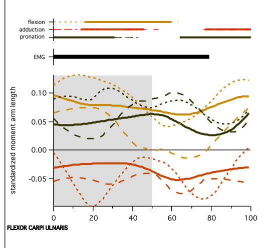	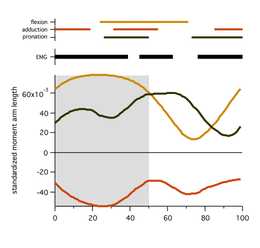	FLEX[J] or EXT<br>ADD<br>SUP	same	x, 11-26<br>8-11<br>21-36	13<br>7<br>18	No previously suggested functions (at elbow)	**EXT -> opposes FLEX;**<br>**ADD or opposes ABD;**<br>**opposes PRO, or SUP**
	Extensor metacarpi radialis**	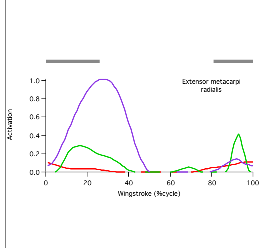	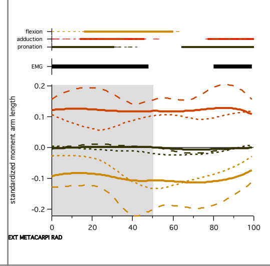	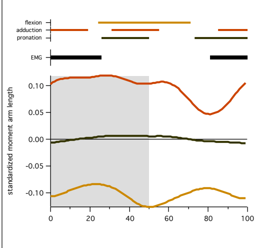	FLEX<br>ABD<br>PRO or SUP	same	9-16<br>26-38<br>x, x	11<br>27<br>x	No previously suggested functions (at elbow)	**FLEX or opposes EXT;**<br>**ABD or opposes ADD**
	Extensor digitorum communis**	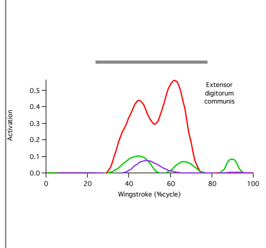	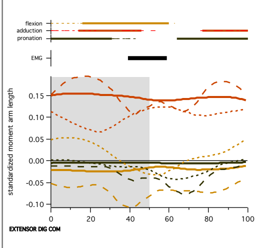	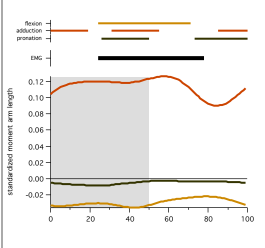	FLEX or EXT[B]<br>ABD<br>PRO	same	x, x<br>5-8<br>x	x<br>6<br>x	No previously suggested functions (at elbow)	**mainly ABD**
	Extensor carpi ulnaris**	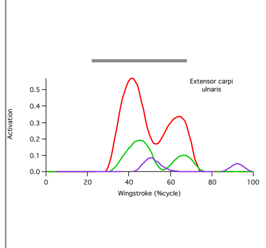	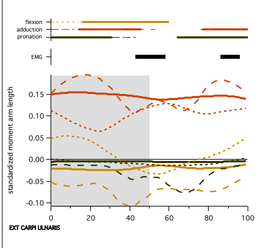	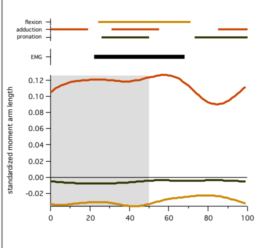	FLEX or EXT[B]<br>ABD<br>PRO	same	x, x<br>7-9<br>x	x<br>10<br>x	No previously suggested functions (at elbow)	**ABD, or opposes ADD**

Table S8 (parts A, B, C). Muscle functions based on muscle moment arms and co-occurrence of activation and kinematics.

Behaviors: WAIR = wing-assisted incline running, AF = ascending flight. Kinematics: DEP = depression, ELEV = elevation, RET = retraction, PROT = protraction, PRO = pronation, SUP = supination, FLEX = flexion, EXT = extension, ADD = adduction, ABD = abduction; DS = downstroke, US = upstroke. Superscripts: A = adult model (>100 days), J = juvenile model (18-20 days), B = baby model (7-8 days); if no superscript, kinematic rotation occurs in all models. Text color, potential function column: black = consistent w/ previously suggested function, red = inconsistent w/ previously suggested function, blue = no previously suggested function; bold = main function. Cell color, right column: green = DS muscle or muscle not active during DS-US transition, blue = US muscle, active during DS-US transition. **: biarticular muscle. *: not tested here (digit kinematics).

C

	Muscle	EMG timing (colored lines = WAIR simulations, Adult, Juvenile, or Baby model; gray bars = AF, from Dial 1992)	Moment arms (Adult solid lines, Juvenile large dash, Baby small dash) + timing of activity (WAIR, simulated; AF from Dial 1991 & 1992) + kinematics (from Heers et al. 2016)		Potential function based on moment arms and phys x-sectional area (Table 2) (black: consistent with previous data, blue: no previous data, red: inconsistent with previous data)		Relative importance (average % torque from Table 2)		Reason for discrepancy with previous work (Dial 1992, Poore 1997, Biewener 2011, Berg & Biewener 2012) on flight?	Suggested function based on moment arms and co-occurrence of EMG and kinematics; green = downstroke muscle, blue = upstroke muscle
			WAIR	AF	WAIR	AF (adult only)	WAIR	AF (adult only)		
WRIST	Flexor carpi ulnaris**	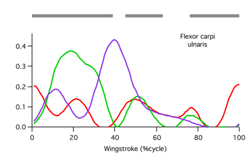	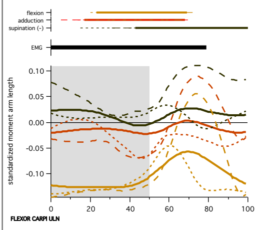	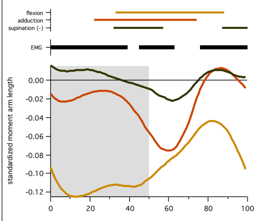	FLEX or EXT^{J}<br>ADD or ABD^{J}<br>PRO or SUP^{AB}	FLEX<br>ADD or ABD<br>PRO or SUP	89-99, x<br>22-53, 8-87<br>46-64, 8-25	94<br>37, 69<br>27, 65	Consistent; EXT moment in J small and brief	**FLEX or opposes EXT;**<br>ADD or opposes ABD;<br>PRO or opposes SUP
	Flexor digitorum sublimis, profundus	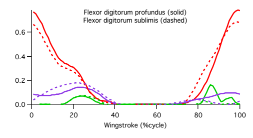	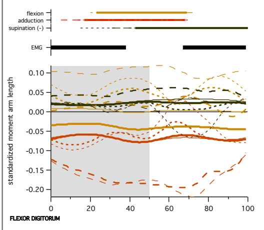	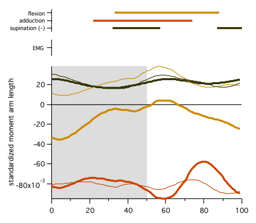	EXT^{BJ} or FLEX^{A}<br>ADD<br>PRO or SUP^{B}	EXT or FLEX<br>ADD<br>PRO	11-15, x<br>38-64<br>17-83, 29	x<br>52<br>35	No previously suggested functions (at wrist); main function may be to ADD phalanges (FLEX in human anatomy)	EXT^{BJ}, opposes FLEX, FLEX^{A}, opposes EXT^{A};<br>**mainly opposes ABD;**<br>**PRO or opposes SUP**
	Ulnimetacarpalis ventralis	EMG very low	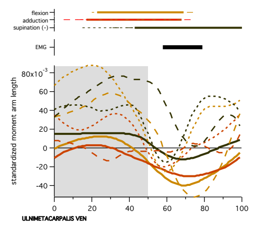	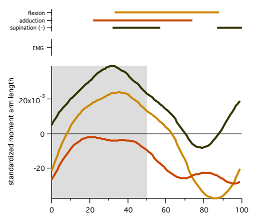	FLEX or EXT<br>ADD or ABD^{B}<br>PRO or SUP^{AB}	same	4-60, x<br>4-6, x<br>8-32, 13-20	6, x<br>x<br>17, 80	No previously suggested functions	does not seem to contribute much during WAIR:<br>FLEX;<br>ADD
	Extensor metacarpi radialis**	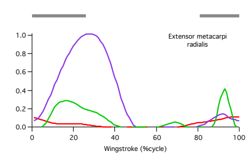	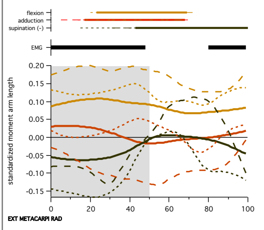	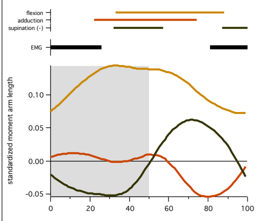	EXT<br>ADD or ABD^{AB}<br>PRO^{J} or SUP	EXT<br>ADD or ABD<br>PRO or SUP	59-67<br>11-36, 21-24<br>14-31, 65-79	60<br>23, 12<br>50, 58	Consistent	**EXT, also opposes FLEX;**<br>ABD or opposes ABD (WAIR^{J}, AF);<br>**SUP or opposes PRO**
	Extensor digitorum communis**	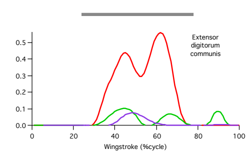	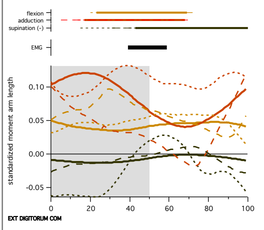	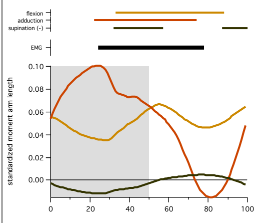	EXT<br>ADD^{J} or ABD<br>PRO^{BJ} or SUP	EXT<br>ADD or ABD<br>PRO or SUP	3-6<br>x, 28-30<br>x, 8-10	5<br>x, 24<br>x, x	Consistent - ADD moment in J brief and does not occur when muscle is activated. Should also ABD phalanges (EXT in human anatomy)	**mainly opposes FLEX;**<br>**mainly opposes ADD**;<br>SUP or opposes PRO
	Extensor carpi ulnaris**	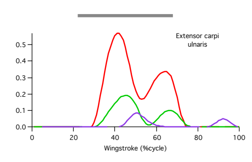	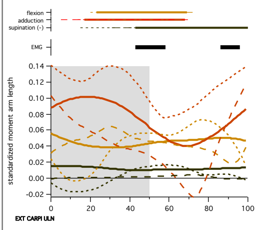	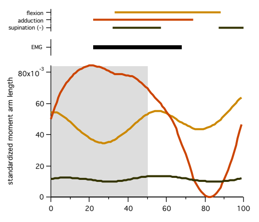	EXT<br>ADD^{J} or ABD<br>PRO^{AB} or SUP^{B}	same	3-9<br>x, 33-52<br>6-14, x	8<br>34<br>8	Some texts say EXT, some say FLEX; activated during wrist flexion but actually serves to stabilize wrist - tendons of Extensor digitorum communis and Extensor carpi ulnaris pass through retinaculum on distal ulna, preventing flexion	**EXT but mainly opposes FLEX**<br>**ABD but mainly opposes ADD**;<br>PRO but mainly opposes SUP
	Extensor pollicis longus	EMG very low	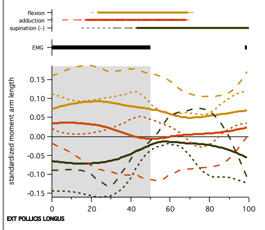	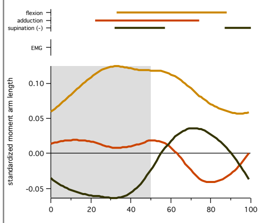	EXT<br>ABD^{AB} or ADD$^{J>BA}$<br>PRO^{J} or SUP	same	3-16<br>8, x<br>x, 6-57	16<br>7, 8<br>9, 32	No previously suggested functions (at wrist); main function may be to extend pollex*	does not seem to contribute much to wrist movement during WAIR:<br>EXT or opposes FLEX;<br>ABD or opposes ADD;<br>opposes PRO
	Extensor indices longus	EMG very low	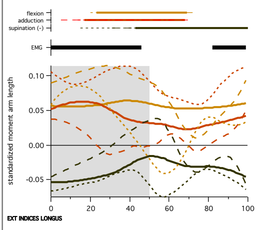	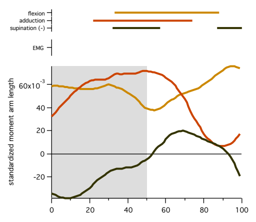	EXT or FLEX^{B}<br>ADD^{J} or ABD<br>PRO^{J} or SUP	same	3-7, x<br>x, 9-13<br>x, 10-38	6<br>23<br>7	No previously suggested functions (at wrist). Should also ABD interphalangeal joints* (EXT in human anatomy)	does not seem to contribute much to wrist movement during WAIR:<br>EXT or opposes FLEX;<br>ABD or opposes ADD;<br>opposes PRO
