# Supplementary material for: Building a Bird: Musculoskeletal Modeling and Simulation of Wing-Assisted Incline Running During Avian Ontogeny
Source: Front Bioeng Biotechnol. 2018 Oct 23;6:140. doi: 10.3389/fbioe.2018.00140 (PMC6205952; doi:10.3389/fbioe.2018.00140)
Supplement: Supplementary file 12 [file Image_1.PDF]

Figure S1. Distribution of mass. Different body segments, and their masses (in grams), indicated by different colors. Image of adult chukar.

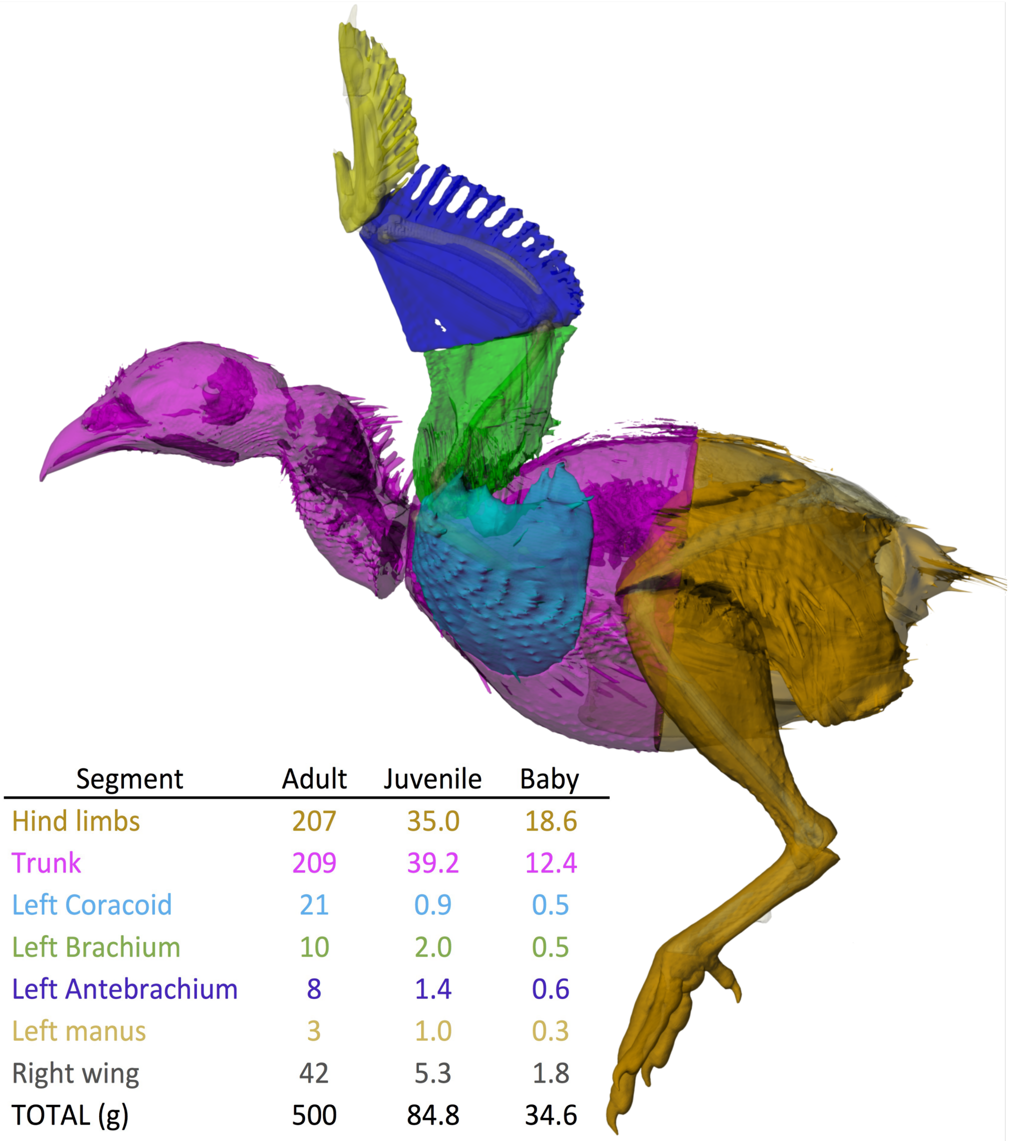

Segment	Adult	Juvenile	Baby
Hind limbs	207	35.0	18.6
Trunk	209	39.2	12.4
Left Coracoid	21	0.9	0.5
Left Brachium	10	2.0	0.5
Left Antebrachium	8	1.4	0.6
Left manus	3	1.0	0.3
Right wing	42	5.3	1.8
TOTAL (g)	500	84.8	34.6
