# Supplementary material for: Building a Bird: Musculoskeletal Modeling and Simulation of Wing-Assisted Incline Running During Avian Ontogeny
Source: Front Bioeng Biotechnol. 2018 Oct 23;6:140. doi: 10.3389/fbioe.2018.00140 (PMC6205952; doi:10.3389/fbioe.2018.00140)

Figure S2. Hill-type muscle model used to define modeled muscles. Each modeled muscle contains an active contractile element (CE; muscle fibers) operating in parallel with a passive elastic element (PE; e.g., connective tissue associated with muscle belly) and in series with a series elastic element (SEE; elastic components of tendon(s)). In static optimization simulations, muscle force (F_m) is proportional to fiber length (L_f) and activation, and tendon force (F_t) is equal to F_m*cosα, where α is the average pennation angle of the muscle fibers. The length of the entire muscle-tendon unit (L_{mt}) is the sum of the length of the tendon(s) (L_t) and muscle belly (L_m). Modified from (Charles et al., 2016; Zajac, 1989).

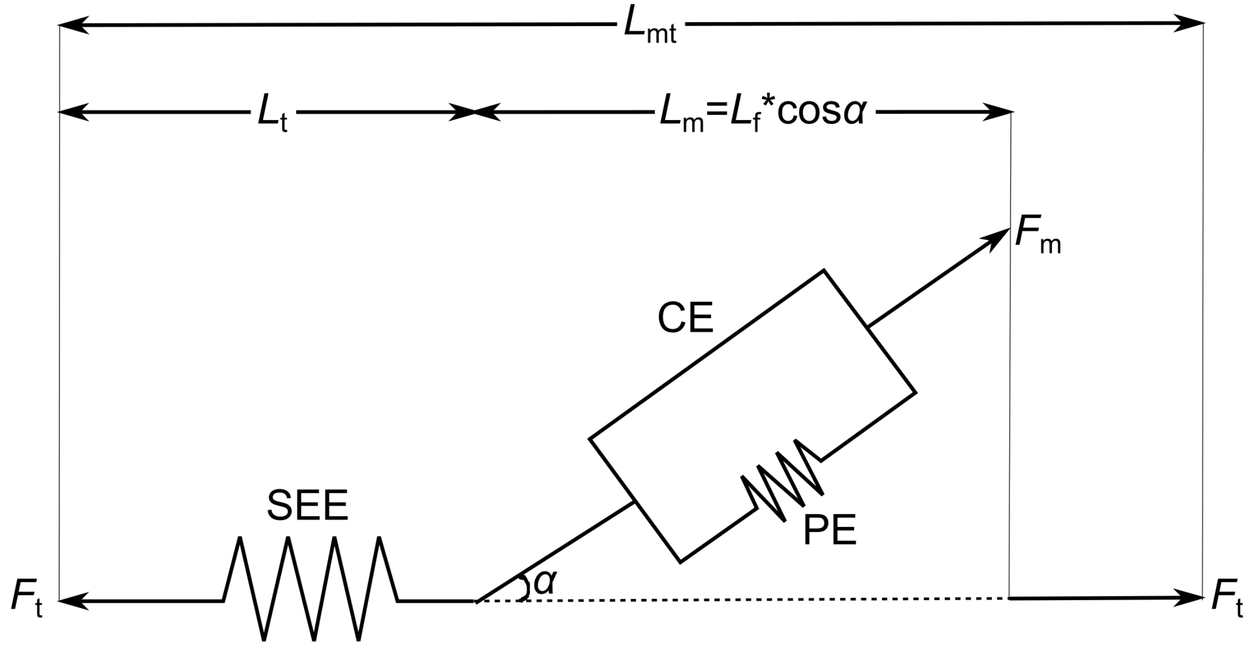

Supplement: Supplementary file 13 [file Image_2.PDF]
