# Supplementary material for: Building a Bird: Musculoskeletal Modeling and Simulation of Wing-Assisted Incline Running During Avian Ontogeny
Source: Front Bioeng Biotechnol. 2018 Oct 23;6:140. doi: 10.3389/fbioe.2018.00140 (PMC6205952; doi:10.3389/fbioe.2018.00140)

Figure S3. Timing of aerodynamic force production. The timing of aerodynamic force production used in simulations (A) is consistent with the timing of force production measured *in vivo* (i.e., peaks in mid-downstroke) (B). **(A)**: aerodynamic force produced by the manual ("hand") segment of the adult chukar wing, during the downstroke; **(B)** modified from (Lentink et al., 2015), shaded areas represent downstrokes.

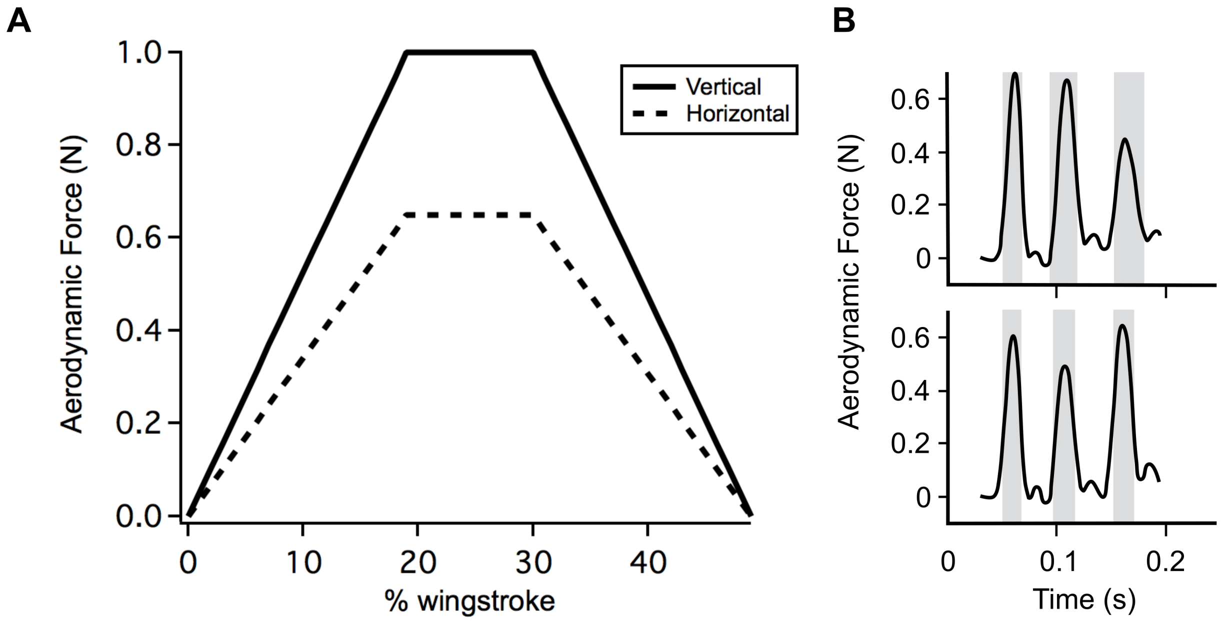

Supplement: Supplementary file 14 [file Image_3.pdf]
