# Supplementary material for: Building a Bird: Musculoskeletal Modeling and Simulation of Wing-Assisted Incline Running During Avian Ontogeny
Source: Front Bioeng Biotechnol. 2018 Oct 23;6:140. doi: 10.3389/fbioe.2018.00140 (PMC6205952; doi:10.3389/fbioe.2018.00140)

Figure S4. Limitations of Static Optimization. Shoulder joint moments (black lines) during three flaps of wing-assisted incline running, in the adult model. Simulations assuming rigid tendons result in overly stretched muscle fibers less capable of generating force, and high contributions from reserve actuators (red lines; pectoralis and supracoracoideus stretched to 1.5 fiber lengths at upstroke-downstroke and downstroke-upstroke transition, respectively). Ignoring force-length relationships reduces, but does not eliminate, contributions from reserve actuators (blue lines; similar patterns in baby and juvenile models; peak reserve moments, as a percentage of total moment, are generally less in baby and juvenile than adult simulations) (Table S5). The remaining moment contributed by reserve actuators (blue lines) likely represents contributions by elastic tendons / ligaments, which cannot be accurately captured by static optimization.

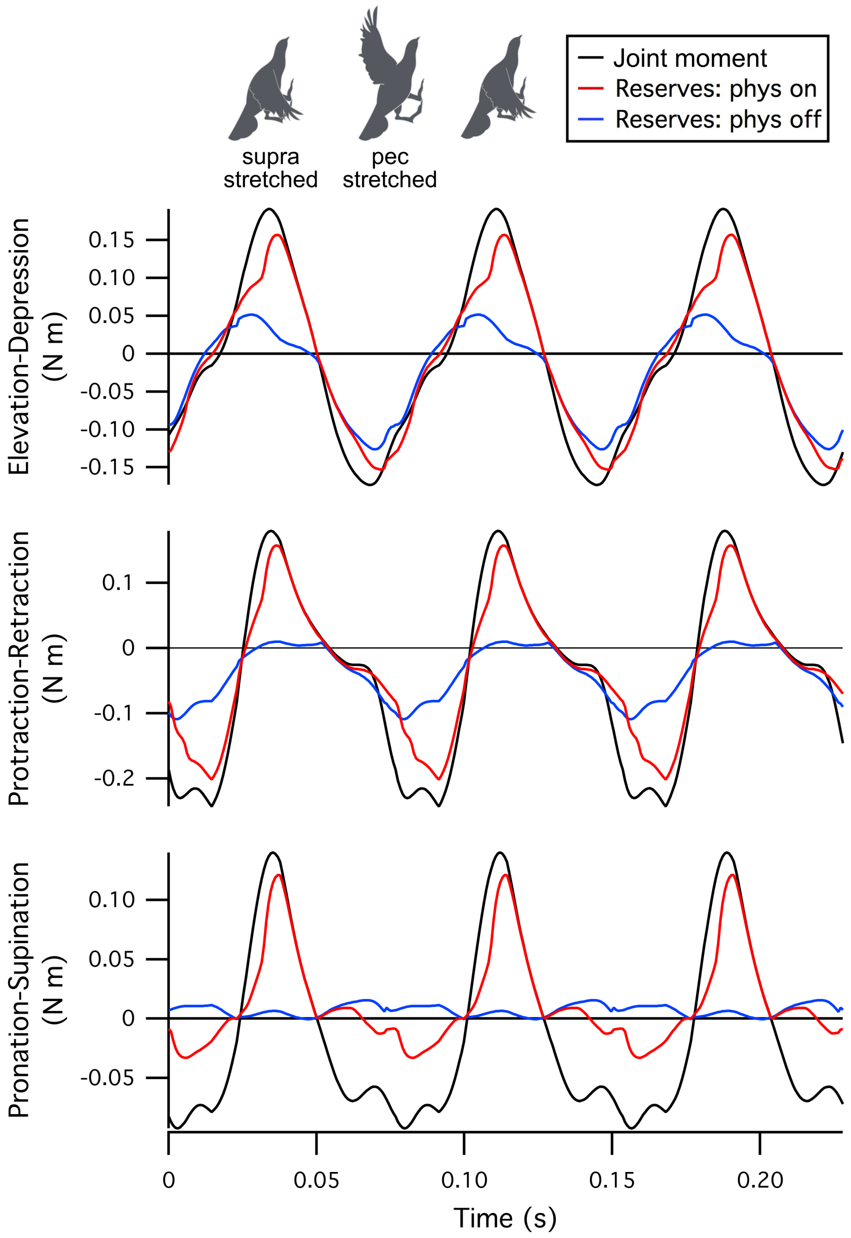

Supplement: Supplementary file 15 [file Image_4.pdf]
