# Supplementary material for: Building a Bird: Musculoskeletal Modeling and Simulation of Wing-Assisted Incline Running During Avian Ontogeny
Source: Front Bioeng Biotechnol. 2018 Oct 23;6:140. doi: 10.3389/fbioe.2018.00140 (PMC6205952; doi:10.3389/fbioe.2018.00140)

Figure S5. Modeling the pectoralis muscle. (A) The pectoralis is the largest flight muscle, with a complex fiber arrangement and a very broad origin spanning across the sternum and keel, ribs, and furcula (Table S1). **(B)** We modeled the pectoralis as a single pennate muscle following a path through the center of the volume of the muscle. An alternative method is to model the pectoralis as three separate muscles to account for the broad origin. Modeling the pectoralis as three muscles (light purple dashed lines; "1", "2", "3" in (A)) instead of a single muscle (dark purple solid line, equivalent to "2" in (A)) does not alter the timing of pectoralis activation but does reduce its average level of activation (light purple solid line), most likely due to the greater range of moment arm orientations. Modeling the pectoralis as three muscles additionally reduces activation of the Coracobrachialis posterior and Scapulohumeralis Caudalis muscles. Data for adult chukar.

A. Left lateral view of adult pectoralis muscle

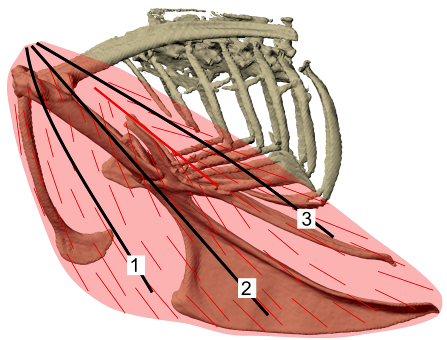

B. Muscle activations during wing-assisted incline running: pectoralis modeled as one versus three muscles

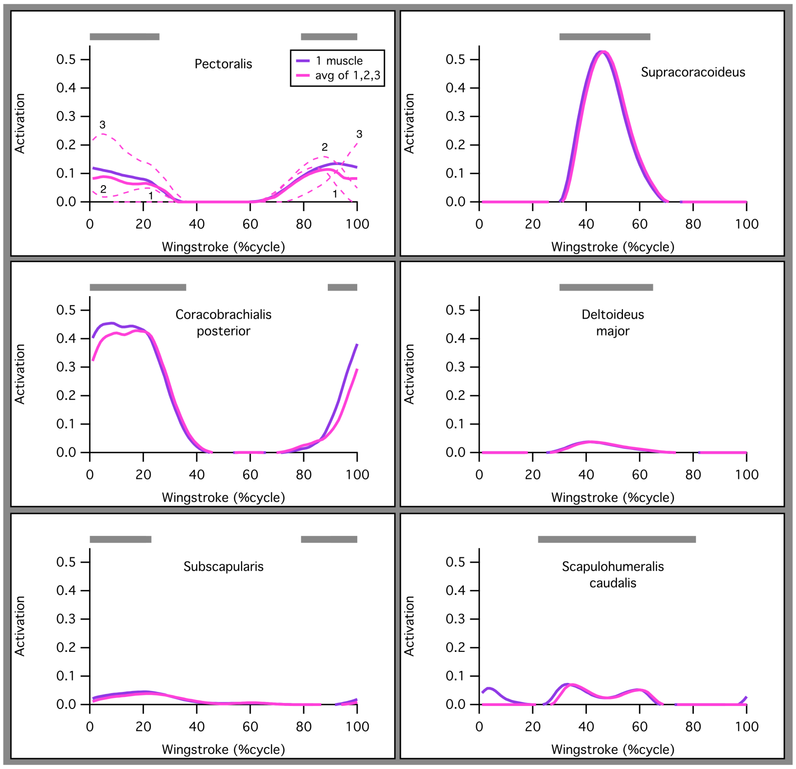

Supplement: Supplementary file 16 [file Image_5.pdf]
