# Supplementary material for: Building a Bird: Musculoskeletal Modeling and Simulation of Wing-Assisted Incline Running During Avian Ontogeny
Source: Front Bioeng Biotechnol. 2018 Oct 23;6:140. doi: 10.3389/fbioe.2018.00140 (PMC6205952; doi:10.3389/fbioe.2018.00140)

Figure S6. Normalized muscle mass. Muscle mass – in terms of percent body mass – increases proportionally through ontogeny (data from (Heers and Dial, 2015)), with muscles acting at the elbow and wrist reaching adult size more quickly than muscles acting at the shoulder.

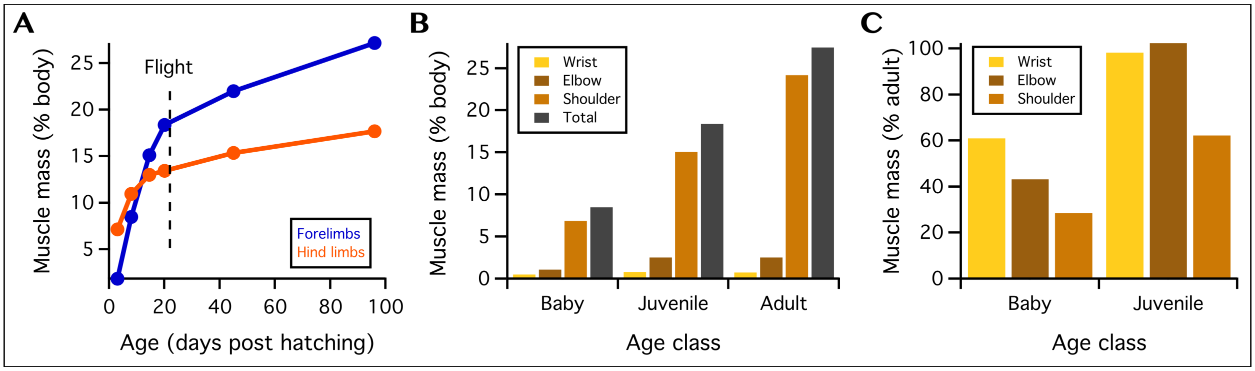

Supplement: Supplementary file 17 [file Image_6.PDF]
