# Supplementary material for: Building a Bird: Musculoskeletal Modeling and Simulation of Wing-Assisted Incline Running During Avian Ontogeny
Source: Front Bioeng Biotechnol. 2018 Oct 23;6:140. doi: 10.3389/fbioe.2018.00140 (PMC6205952; doi:10.3389/fbioe.2018.00140)

Figure S7. Muscle force versus activation. Simulations with no aerodynamic force and adult kinematics suggest that baby, juvenile, and adult chukar muscles produce relatively similar magnitudes of force (overlapping loops in A, B) for their body size and level of activation. Force is expressed in multiples of body weight; L, muscle lengthening, S, muscle shortening.

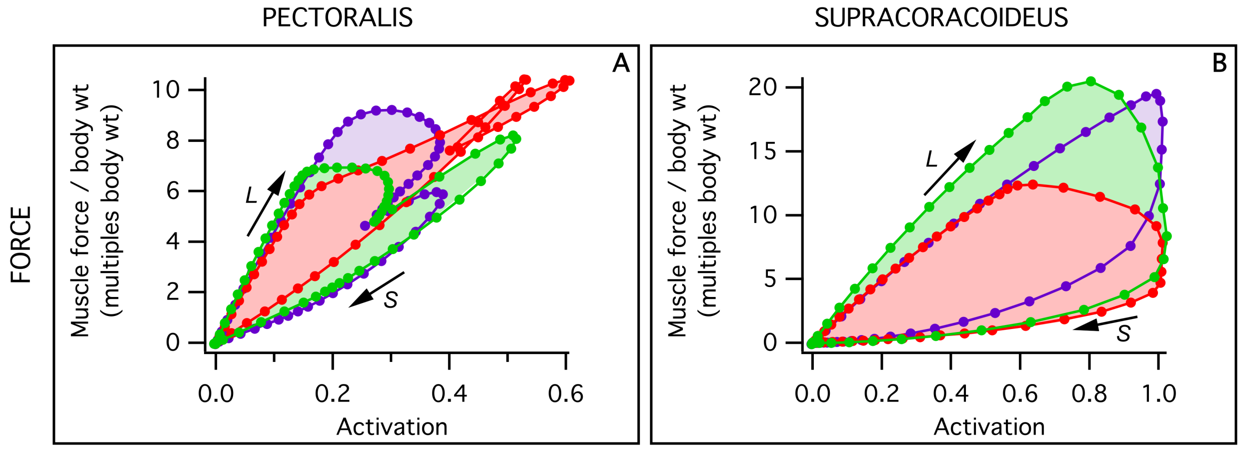

Supplement: Supplementary file 18 [file Image_7.PDF]
