# Supplementary material for: Building a Bird: Musculoskeletal Modeling and Simulation of Wing-Assisted Incline Running During Avian Ontogeny
Source: Front Bioeng Biotechnol. 2018 Oct 23;6:140. doi: 10.3389/fbioe.2018.00140 (PMC6205952; doi:10.3389/fbioe.2018.00140)

Figure S8. Feather lengths. Juvenile birds have proportionally long feathers compared to adults and babies, except for feathers that are just beginning to emerge from the sheath (P8, P9, S1, S12, S13, S14; absent or very small in babies).

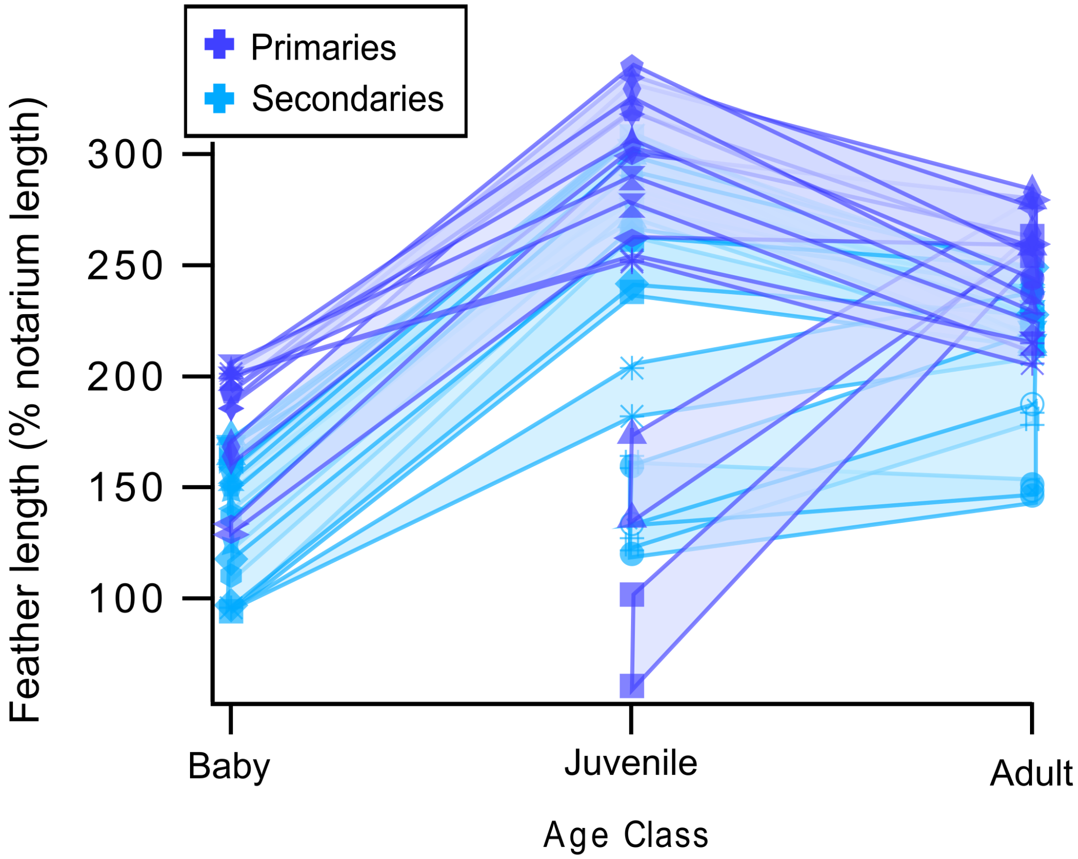

Supplement: Supplementary file 19 [file Image_8.PDF]
